# Pembrolizumab 400 mg every 6 weeks as first-line therapy for advanced melanoma (KEYNOTE-555): Results from cohort B of an open-label, phase 1 study

**DOI:** 10.1371/journal.pone.0309778

**Published:** 2024-11-12

**Authors:** Graham Cohen, Bernardo Rapoport, Sze W. Chan, Paul Ruff, Ana Arance, Karmele Mujika Eizmendi, Baerin Houghton, Michael P. Brown, Robert M. Zielinski, Eva Muñoz Couselo, Megan Lyle, James R. Anderson, Lokesh Jain, Dinesh de Alwis, Mallika Lala, Omobolaji Akala, Elliot Chartash, Conrad Jacobs

**Affiliations:** 1 Mary Potter Oncology Centre, Pretoria, South Africa; 2 The Medical Oncology Centre of Rosebank, Johannesburg, South Africa; 3 Department of Immunology, Faculty of Health Sciences, University of Pretoria, Pretoria, South Africa; 4 Sandton Oncology, Johannesburg, South Africa; 5 University of Witwatersrand Faculty of Health Sciences, Johannesburg, South Africa; 6 Hospital Clínic de Barcelona, Barcelona, Spain; 7 Onkologikoa Donostia, San Sebastian, Spain; 8 Port Macquarie Base Hospital, Port Macquarie, New South Wales, Australia; 9 Cancer Clinical Trials Unit, Royal Adelaide Hospital, and School of Medicine, The University of Adelaide, Adelaide, South Australia, Australia; 10 Central West Cancer Care Centre, Orange Hospital, Orange, New South Wales, Australia; 11 Western Sydney University, Sydney, New South Wales, Australia; 12 Department of Medical Oncology, Melanoma and Other Skin Cancers Unit, Vall d’Hebron University Hospital, Barcelona, Spain; 13 Liz Plummer Cancer Care Centre, Cairns, Queensland, Australia; 14 Merck & Co., Inc., Rahway, New Jersey, United States of America; 15 Cancer Care: Clinical & Radiation Oncology, Cape Town, South Africa; Rutgers University, UNITED STATES OF AMERICA

## Abstract

Intravenous pembrolizumab 400 mg every 6 weeks was approved across tumor types based on pharmacokinetic modeling, which showed exposures consistent with previous standard dosing of 200 mg or 2 mg/kg every 3 weeks, and early results of cohort B of the phase 1 KEYNOTE-555 study. Results after ≥1 year of potential follow-up for all patients in cohort B of KEYNOTE-555 are presented. Patients aged ≥18 years with previously untreated stage III/IV melanoma received pembrolizumab 400 mg every 6 weeks for ≤18 cycles. The primary endpoint was objective response rate per RECIST v1.1 by blinded independent central review. Secondary endpoints included duration of response, progression-free survival, pharmacokinetics, and safety. Overall, 101 patients received pembrolizumab. Median projected follow-up was 21.9 months (range, 17.0–25.7). The objective response rate was 50.5% (95% CI: 40.4–60.6; 19 complete responses, 32 partial responses). Median duration of response was not reached (NR; range, 2.4+ to 21.0+ months). Median progression-free survival was 13.8 months (95% CI: 4.1–NR). Observed pharmacokinetic exposures were consistent with model predictions for pembrolizumab 400 mg every 6 weeks and other approved and tested schedules (2 mg/kg or 200 mg every 3 weeks). Grade 3–4 treatment-related adverse events occurred in 13 patients (12.9%). No deaths were considered treatment related. These results support the pharmacokinetic modeling and demonstrate that the benefit-risk profile of pembrolizumab 400 mg Q6W is consistent with that of 200 mg or 2 mg/kg every 3 weeks. Clinically meaningful objective response rate and durable progression-free survival within the expected range for first-line pembrolizumab were observed.

**Clinical trial registry:** ClinicalTrials.gov, NCT03665597.

## Introduction

Immune checkpoint inhibitors have a proven role in the treatment of cancer; however, until recently, intravenous infusion was required every 2–4 weeks. Extending the interval between administrations has the potential to provide numerous benefits, including improving patient experience, lessening the burden on infusion services, and reducing healthcare costs [1]. The option to extend the dosing interval became particularly relevant during the coronavirus disease 2019 pandemic, when fewer visits to infusion centers reduced the risk of vulnerable patients being exposed to infection with severe acute respiratory syndrome coronavirus 2.

Pembrolizumab is a monoclonal antibody directed against programmed cell death protein 1 (PD-1) with a well-established safety and efficacy profile across multiple tumor types [2]. Pembrolizumab was initially approved by various regulatory agencies for use at a dose of 2 mg/kg every 3 weeks, and subsequently at a fixed dose of 200 mg every 3 weeks based on pharmacokinetic analysis that showed similar exposure distributions for these schedules [3]. More recently, pembrolizumab has been approved at a dose of 400 mg every 6 weeks across all currently approved adult indications based on pharmacokinetic modeling and exposure–response analyses [2, 4, 5]. The model-based analysis simulated concentration-time profiles using an established pharmacokinetic population of 2993 patients with melanoma or non–small cell lung cancer from five clinical trials [6]. The results showed that pembrolizumab 400 mg administered every 6 weeks led to exposures similar to those of the approved dosing schedules of 200 mg or 2 mg/kg administered every 3 weeks and below the exposure of the highest clinically evaluated dose of 10 mg/kg administered every 2 weeks. These findings were consistent with the known exposure–response relationship for pembrolizumab, in which similar efficacy and safety have been observed with an ≥5-fold range of dosing [7, 8]. The approval was also supported by an early analysis of cohort B of the KEYNOTE-555 study (data cut-off, February 6, 2020), which showed no loss of efficacy and a similar safety profile between the dosing schedules of pembrolizumab 400 mg every 6 weeks and 200 mg or 2 mg/kg every 3 weeks in patients with advanced melanoma [9]. Results from a later analysis of cohort B (data cut-off, August 6, 2020) showed that observed exposures with pembrolizumab 400 mg every 6 weeks were consistent with model-based simulations, and that the schedule yielded a clinically meaningful objective response rate of 50.5% (95% CI: 40.4–60.6) and a median progression-free survival of 13.8 months (95% CI: 3.0 to not reached) [10]. Here, we present further updated results after ≥1 year of potential follow-up for all patients in cohort B of the KEYNOTE-555 study.

## Materials and methods

### Study design and participants

KEYNOTE-555 (ClinicalTrials.gov, NCT03665597 https://clinicaltrials.gov/study/NCT03665597) is an open-label multicohort clinical trial investigating pembrolizumab in patients with advanced melanoma. Cohort A was a randomized crossover study of the bioavailability of subcutaneous versus intravenous pembrolizumab [11]. Cohort B investigated the pharmacokinetics, efficacy, and safety of intravenous pembrolizumab at a dose of 400 mg every 6 weeks. Cohort B is the focus of the current report.

Eligible patients were aged ≥18 years, had unresectable stage III or IV melanoma (as per the *American Joint Committee on Cancer [AJCC] Cancer Staging Manual*, 7th edition [12]) that was not amenable to local therapy, had measurable disease as per Response Evaluation Criteria in Solid Tumors version 1.1 (RECIST v1.1), and had an Eastern Cooperative Oncology Group (ECOG) performance status of 0 or 1. Patients were excluded if they had received prior treatment for advanced or metastatic disease, with the exception of patients with *BRAF*^V600^-mutant disease who could have received prior standard-of-care targeted therapy (e.g. BRAF/MEK inhibitors). Full eligibility criteria are included in the trial protocol.

The study protocol and all amendments were approved by the appropriate ethics committee at each center (Pharma-Ethics (Pty) Ltd on February 25, 2019; reference number: 181121924). The study was conducted in accordance with the protocol, its amendments, standards of Good Clinical Practice, and the Declaration of Helsinki. All patients provided written informed consent prior to enrollment.

### Procedures and outcomes

Patients received pembrolizumab 400 mg intravenously every 6 weeks for up to 18 cycles or until disease progression, unacceptable toxicity, or investigator or patient decision to withdraw, whichever comes first. Computed tomography scan or magnetic resonance imaging was performed at week 12, every 9 weeks until week 52, and every 12 weeks thereafter until disease progression, start of new anticancer therapy, withdrawal of consent, or death, whichever occurs first. Response and disease progression were assessed as per RECIST v1.1 by blinded independent central review (BICR). Patients who experienced disease progression or started new anticancer therapy were followed for survival every 12 weeks or to withdrawal of consent. Blood samples for pharmacokinetic analysis were taken 0–4 hours before pembrolizumab administration on day 1 of cycles 1, 2, 4, and 5; at the end of the infusion on day 1 of cycles 1 and 4; and at any time on day 22 (±6 days) of cycles 1 and 4. Adverse events, vital signs, and laboratory abnormalities were monitored throughout the study and for 30 days after treatment cessation. Serious adverse events were monitored for 90 days after treatment cessation or for 30 days if new anticancer therapy was initiated. Adverse events were graded according to the National Cancer Institute Common Terminology Criteria for Adverse Events, version 4.0.

The primary endpoint for cohort B was objective response rate as per RECIST v1.1 by BICR. Secondary endpoints included duration of response and progression-free survival as per RECIST v1.1 by BICR, pharmacokinetics, and safety.

### Statistical analysis

A sample size of approximately 100 was planned based on a precision estimation of a 95% CI half-width of 10%. Efficacy and safety were assessed in all patients who received ≥1 dose of study treatment. The 95% CI for the objective response rate was calculated using the exact binomial method. Duration of response and progression-free survival were estimated using the Kaplan-Meier method. Statistical analyses were conducted using SAS version 9.4 (SAS Institute, Cary, NC, USA).

Pharmacokinetics were assessed in the per-protocol population, which comprised all patients who had data available from ≥1 dose of study treatment. Concentration-time profiles and pharmacokinetic exposures (maximum serum concentration [C_max_] and trough serum concentration [C_trough_]) observed during initial treatment (cycle 1; weeks 1–6) and at steady state (cycle 4; weeks 19–24) were compared against model-based simulations of a schedule of 400 mg every 6 weeks from a reference population of 2993 patients with melanoma or non–small cell lung cancer from KEYNOTE-001, KEYNOTE-002, KEYNOTE-006, KEYNOTE-010, and KEYNOTE-024 [6]. The observed pharmacokinetic exposures (C_max_ and C_trough_) from patients in KEYNOTE-555 were also compared with simulated pharmacokinetic exposures from the reference population for pembrolizumab 2 mg/kg every 3 weeks, 200 mg every 3 weeks, and 10 mg/kg every 2 weeks. Geometric means for C_max_ were calculated for each patient based on observed concentration values at the end of the pembrolizumab infusion (30 minutes after the dose on day 1 of cycles 1 and 4), and geometric means for C_trough_ were calculated based on observed concentration values at the end of the dosing interval (day 42 of cycles 1 and 4).

## Results

**Fig 1:**
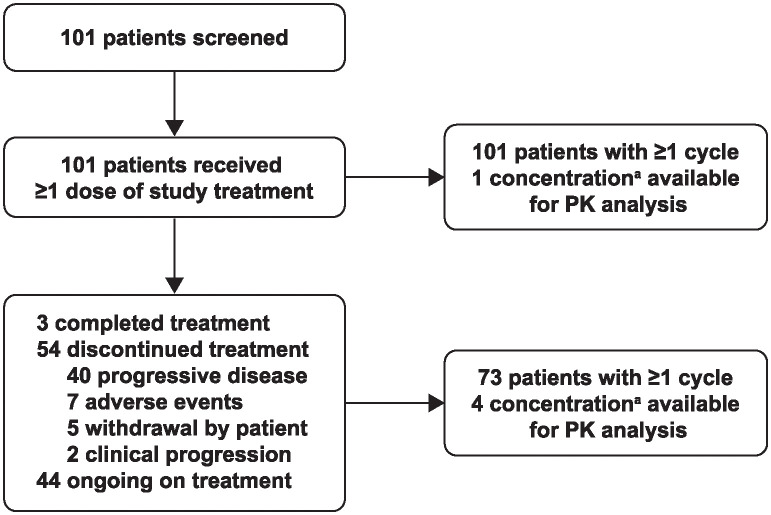
KEYNOTE-555 cohort B CONSORT flow diagram. PK, pharmacokinetics. ^a^Maximum serum concentration (C_max_) or trough serum concentration (C_trough_) or both.

Between May 2019 and January 2020, 101 patients were screened; all 101 patients were enrolled in the study and received treatment with pembrolizumab (Fig 1). At the time of the data cut-off (July 6, 2021), 3 patients had completed treatment; 54 had discontinued treatment, primarily due to progressive disease (40 of 54); 44 remained on treatment; and none had died. The median age was 64.0 years, 65 of 101 patients (64.4%) were male, 66 patients (65.3%) had an Eastern Cooperative Oncology Group performance status of 0, and 96 patients (95.0%) had stage IV disease (Table 1). The majority of patients (94 of 101 [93.1%]) had received no prior therapy; 5 patients (5.0%) had received prior adjuvant therapy, and 2 patients (2.0%) with *BRAF*-mutated disease had received prior BRAF/MEK inhibitor therapy. At data cut-off, the median projected follow-up, defined as the time from first dose to data cut-off, was 21.9 months (range, 17.0–25.7); the median duration of treatment was 15.5 months (range, 1 day to 23.8 months), and patients had received a median of 12 administrations of pembrolizumab (range, 1–18).

**Table 1:** Baseline demographics and disease characteristics.

	N = 101
**Age, median (range), years**	64 (27–88)
**Sex**	
Male	65 (64.4)
Female	36 (35.6)
**ECOG performance status**	
0	66 (65.3)
1	35 (34.7)
**LDH concentration**	
≤ULN	60 (59.4)
>ULN but <2 × ULN	32 (31.7)
≥2 × ULN	8 (7.9)
Missing	1 (1.0)
**Disease stage** ^a^	
III	5 (5.0)
IV	96 (95.0)
**Metastatic stage**	
M0	3 (3.0)
M1a	29 (28.7)
M1b	29 (28.7)
M1c	39 (38.6)
Missing	1 (1.0)
**Prior therapy**	
One prior therapy for advanced *BRAF*-mutated disease	2 (2.0)
Prior adjuvant therapy	5 (5.0)
No prior therapy	94 (93.1)
***BRAF* status and prior BRAF/MEK inhibitor treatment**	
*BRAF* wild type	36 (35.6)
*BRAF* mutated, no prior BRAF/MEK inhibitor therapy	27 (26.7)
*BRAF* mutated, prior BRAF/MEK inhibitor therapy	2 (2.0)
Undetermined	36 (35.6)

AJCC, American Joint Committee on Cancer; ECOG, Eastern Cooperative Oncology Group; LDH, lactate dehydrogenase; ULN, upper limit of normal.

Data are n (%) unless otherwise stated.

^a^As per the *AJCC Cancer Staging Manual*, 7th edition.

### Efficacy

**Fig 2:**
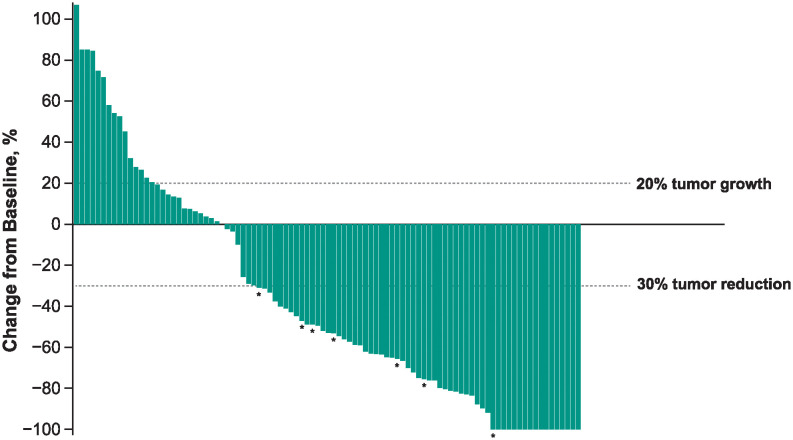
Best percentage change from baseline in target lesions as per RECIST v1.1 by BICR^a^. BICR, blinded independent central review; RECIST v1.1, Response Evaluation Criteria in Solid Tumors version 1.1. ^a^Includes patients with baseline and postbaseline target lesion assessment. *Indicates patient had progressive disease in non-target lesions.

**Fig 3:**
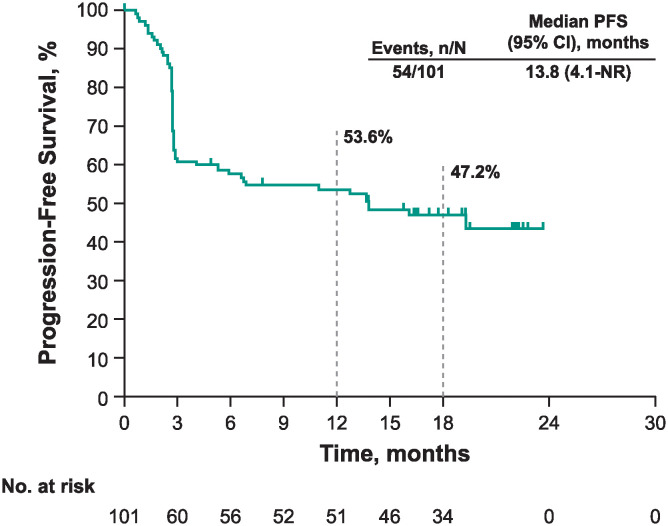
Kaplan-Meier estimate of progression-free survival as per RECIST v1.1 by BICR. BICR, blinded independent central review; NR, not reached; PFS, progression-free survival; RECIST v1.1, Response Evaluation Criteria in Solid Tumors version 1.1.

The confirmed objective response rate was 50.5% (95% CI: 40.4–60.6) (Table 2); 19 of 101 patients (18.8%) had a complete response, and 32 patients (31.7%) had a partial response. Eleven of 101 patients (10.9%) had stable disease. Among patients with a baseline and postbaseline target lesion assessment, most had a reduction in target lesion burden of ≥30% from baseline (Fig 2); response status was not confirmed among these patients. The median time to response was 2.8 months (range, 2.5–9.0), and the median duration of response was not reached (range, 2.4+ to 21.0+) (Table 2). The estimated percent of responders continuing in response was 87.8% at 12 months and 79.6% at 18 months. Median progression-free survival was 13.8 months (95% CI: 4.1 to not reached), and the 12- and 18-month progression-free survival rates were 53.6% and 47.2%, respectively (Fig 3).

**Table 2:** Objective response as per RECIST v1.1 by BICR.

	N = 101
**Objective response rate, % (95% CI)** ^a^	50.5 (40.4–60.6)
**Best response, n (%)**	
Complete response	19 (18.8)
Partial response	32 (31.7)
Stable disease	11 (10.9)
Progressive disease	34 (33.7)
No assessment	5 (5.0)
**Time to response, median (range), months**	2.8 (2.5–9.0)
**Duration of response,**^b^ **median (range), months**	NR (2.4+ to 21.0+)
≥12 months, %	87.8
≥18 months, %	79.6

BICR, blinded independent central review; CI, confidence interval; NR, not reached; RECIST v1.1, Response Evaluation Criteria in Solid Tumors version 1.1.

“^+^” indicates that there was no progressive disease at the time of the last disease assessment.

^a^Based on binomial exact CI method.

^b^From Kaplan-Meier method for censored data.

### Pharmacokinetics

**Fig 4:**
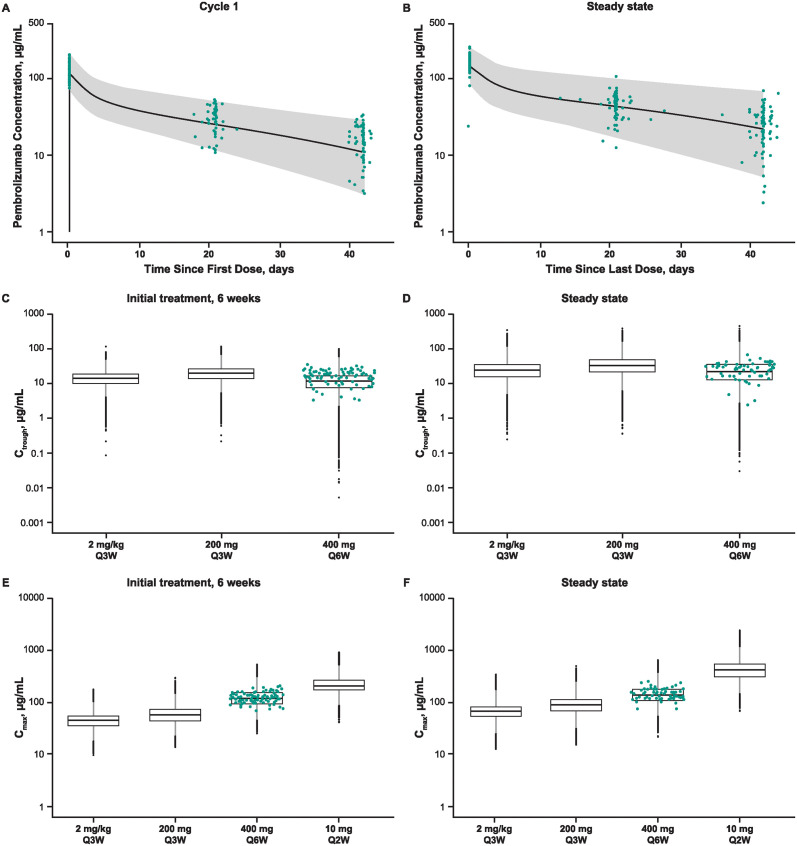
Observed pharmacokinetic data from patients treated with pembrolizumab 400 mg Q6W in cohort B of KEYNOTE-555 compared with (A, B) the model-predicted pharmacokinetic profile for pembrolizumab 400 mg Q6W (median and 90% prediction interval); (C, D) the model-predicted C_trough_ for pembrolizumab 200 mg Q3W, 2 mg/kg Q3W, and 400 mg Q6W; (E, F) and the model predicted C_max_ for pembrolizumab 2 mg/kg Q3W, 200 mg Q3W, 400 mg Q6W, and 10 mg/kg Q2W. The green dots are the observed pharmacokinetic data from patients treated with pembrolizumab 400 mg Q6W in cohort B of KEYNOTE-555. C_max_, maximum serum concentration; C_trough_, trough serum concentration; Q3W, every 3 weeks; Q6W, every 6 weeks.

C_max_, C_trough_, or both were available for 101 patients at initial treatment (cycle 1) and for 73 patients at steady state (cycle 4) (Fig 1). The observed pharmacokinetic profile and exposures of pembrolizumab 400 mg every 6 weeks during initial treatment and at steady state were consistent with the model predictions for this schedule, with observed concentrations within the 90% prediction intervals of simulated concentrations (Fig 4). The observed C_max_ and C_trough_ for pembrolizumab 400 mg every 6 weeks were also consistent with the other approved or tested dosing schedules (Fig 4). The geometric mean of the observed C_max_ at 400 mg every 6 weeks was approximately 42% lower at cycle 1 and approximately 65% lower at steady state than the geometric mean of the observed C_max_ at 10 mg/kg every 2 week at cycle 1 and steady state, respectively. The geometric mean of the observed C_trough_ at 400 mg every 6 weeks was approximately 17% lower at cycle 1 and approximately 22% lower at steady state than the geometric mean of the observed C_trough_ at 200 mg every 3 weeks and was approximately 12% higher at cycle 1 and approximately 4% higher at steady state than the geometric mean of the observed C_trough_ at 2 mg/kg every 3 weeks.

### Safety

All 101 patients (100%) experienced ≥1 adverse event (Table 3). Grade 3–5 adverse events were reported by 43 of 101 patients (42.6%). All-cause adverse events led to treatment discontinuation in 6 of 101 patients (5.9%), and 31 patients (30.7%) experienced a serious adverse event. Three patients died due to an adverse event (one each from pneumonia, sepsis, and respiratory disorder); none of the deaths were considered treatment related as determined by the investigator. Treatment-related adverse events of any grade occurred in 83 of 101 patients (82.2%), the most common of which (≥15%) were vitiligo (n = 24 [23.8%]), arthralgia (n = 22 [21.8%]), pruritus (n = 19 [18.8%]), and fatigue (n = 18 [17.8%]) (Table 4). Grade 3–4 treatment-related adverse events occurred in 13 patients (12.9%); no deaths occurred due to treatment-related adverse events (Table 3). Three patients (3.0%) discontinued treatment due to a treatment-related adverse event. Immune-mediated adverse events or infusion-related reactions occurred in 25 patients (24.8%) (Table 3). No deaths occurred due to an immune-mediated adverse event or infusion-related reaction.

**Table 3:** Summary of adverse events.

	N = 101
**All-cause adverse events**	
With ≥1 event, any grade	101 (100.0)
With ≥1 event, grade 3–5	43 (42.6)
With ≥1 serious event	31 (30.7)
With ≥1 event that lead to death^a^	3 (3.0)
With ≥1 event that led to treatment discontinuation	6 (5.9)
**Treatment-related adverse events**	
With ≥1 event, any grade	83 (82.2)
With ≥1 event, grade 3–4	13 (12.9)
With ≥1 serious event	4 (4.0)
With ≥1 event that lead to death	0 (0.0)
With ≥1 event that led to treatment discontinuation	3 (3.0)
**Immune-mediated adverse events or infusion-related reactions**	
With ≥1 event, any grade^b^	25 (24.8)
With ≥1 event, grade 3–5^c^	3 (3.0)

^a^One occurrence each of grade 5 pneumonia, sepsis, and respiratory disorder; none were considered treatment related.

^b^Three patients (3.0%) experienced an infusion-related reaction (all grade 2).

^c^No deaths occurred due to an immune-mediated adverse event or infusion-related reaction.

**Table 4:** Treatment-related adverse events occurring in ≥5% of patients.

Event	N = 101	
	Any grade	Grade 3–5
**Vitiligo**	24 (23.8)	0 (0)
**Arthralgia**	22 (21.8)	3 (3.0)
**Pruritus**	19 (18.8)	0 (0)
**Fatigue**	18 (17.8)	0 (0)
**Rash**	14 (13.9)	0 (0)
**Diarrhea**	12 (11.9)	1 (1.0)
**Anemia**	9 (8.9)	5 (5.0)
**ALT increased**	8 (7.9)	2 (2.0)
**Asthenia**	8 (7.9)	1 (1.0)
**Hypothyroidism**	8 (7.9)	0 (0)
**AST increased**	7 (6.9)	1 (1.0)
**Hyperthyroidism**	7 (6.9)	0 (0)
**Lymphopenia**	7 (6.9)	0 (0)
**Blood thyroid-stimulating hormone increased**	5 (5.0)	0 (0)
**Dry mouth**	5 (5.0)	0 (0)
**Nausea**	5 (5.0)	0 (0)
**Proteinuria**	5 (5.0)	0 (0)

ALT, alanine aminotransferase; AST, aspartate aminotransferase.

## Discussion

The results of this analysis after a median projected follow-up of 21.9 months indicate that pembrolizumab 400 mg every 6 weeks is effective and safe as first-line therapy for advanced melanoma, with pharmacokinetic exposures consistent with the dosing schedules of 200 mg or 2 mg/kg every 3 weeks. These results support those of earlier analyses [9, 10]. In the current study, pembrolizumab 400 mg every 6 weeks demonstrated a clinically meaningful objective response rate and a durable progression-free survival within the expected range for first-line PD-1 inhibitor monotherapy in advanced melanoma. The observed pharmacokinetic exposures of pembrolizumab at 400 mg every 6 weeks were consistent with the model predictions for this dosing schedule, and with other approved and tested dosing schedules at initial treatment (cycle 1) and at steady state (cycle 4). Pembrolizumab 400 mg every 6 weeks had manageable safety, with an adverse event profile consistent with the known profile of pembrolizumab.

Extending the period between treatment cycles for cancer therapy is associated with several benefits that ultimately improve the patient experience. Monoclonal antibodies are particularly good candidates for altering dosing schedules due to their wide therapeutic index [7, 8, 13]. Investigating an alternative dosing schedule for pembrolizumab is not without precedent, because the approved dosing schedules for both nivolumab and atezolizumab have been extended based on modeling studies. Nivolumab, initially approved for use at 3 mg/kg every 2 weeks and replaced by 240 mg every 2 weeks, was later approved for use at 480 mg every 4 weeks based on pharmacokinetic modeling and simulation [14–16]. Similarly, atezolizumab, which was initially approved for use at a dose of 1200 mg every 3 weeks, is now also approved for use at a dose of 840 mg every 2 weeks or 1680 mg every 4 weeks based on a pharmacokinetic model showing comparable efficacy and safety to the 3-weekly schedule [17, 18]. To the best of our knowledge, pembrolizumab is currently the only agent with clinical data supporting the findings of modeling and simulation and also the only immune checkpoint inhibitor with an approved dosing interval as long as 6 weeks. Several clinical trials investigating pembrolizumab 400 mg every 6 weeks are underway in various malignancies.

The objective response rate in the current single-arm study (50.5%) was numerically higher than that observed historically for pembrolizumab monotherapy in advanced melanoma. In analyses of patients with advanced melanoma without prior therapy, objective response rates ranging from 39.4% to 45% were reported [19]. These differences might, in part, be explained by better prognostic factors of the KEYNOTE-555 study population, most notably a substantially lower proportion of patients having M1c disease (KEYNOTE-555 cohort B, 38.6% versus 63.6%–72%) [19, 20].

Safety in the current analysis was comparable to that of the known profile of pembrolizumab. Results of a large pooled analysis of the long-term safety of pembrolizumab monotherapy in melanoma reported that 17.7% of patients experienced grade 3–4 treatment-related adverse events, 23.0% experienced immune-mediated adverse events, and only 2 patients (0.1%) died due to treatment-related adverse events [21].

Limitations of this study include its single-arm design and lack of a comparator group. This study was not designed to compare pembrolizumab 400 mg Q6W with 200 mg or 2 mg/kg Q3W, but to evaluate the efficacy and safety of pembrolizumab 400 mg Q6W.

## Conclusion

The results of this analysis from cohort B of KEYNOTE-555 support the pharmacokinetic modeling and the known exposure–response relationship for pembrolizumab. The observed pharmacokinetic exposures were consistent with the model-based predictions and within the range of previous clinical experience for 3-weekly schedules, and treatment with pembrolizumab 400 mg every 6 weeks resulted in clinically meaningful efficacy and manageable safety. Based on the totality of efficacy, safety, and PK results, the benefit-risk profile for pembrolizumab 400 mg every 6 weeks is consistent with that of other approved dosing schedules, supporting the use of the 6-weekly dosing schedule in adult indications.

## Supporting information

S1 Protocol (PDF)

S1 Checklist CONSORT 2010 checklist of information to include when reporting a randomised trial*. (DOCX)
