# Supplementary material for: Pembrolizumab 400 mg every 6 weeks as first-line therapy for advanced melanoma (KEYNOTE-555): Results from cohort B of an open-label, phase 1 study
Source: PLoS One. 2024 Nov 12;19(11):e0309778. doi: 10.1371/journal.pone.0309778 (PMC11556718; doi:10.1371/journal.pone.0309778)
Supplement: S1 Protocol — (PDF) [file pone.0309778.s001.pdf]

Title Page

THIS PROTOCOL AMENDMENT AND ALL OF THE INFORMATION RELATING TO IT ARE CONFIDENTIAL AND PROPRIETARY PROPERTY OF MERCK SHARP & DOHME CORP., A SUBSIDIARY OF MERCK & CO., INC., NJ, U.S.A. (MSD).

Protocol Title: A Phase 1 Randomized Clinical Study of Pembrolizumab (MK-3475) to Evaluate the Relative Bioavailability of Subcutaneous Injection Versus Intravenous Infusion in Participants with Advanced Melanoma (KEYNOTE-555)

Protocol Number: 555-02

Compound Number: MK-3475

Sponsor Name:

Merck Sharp & Dohme Corp., a subsidiary of Merck & Co., Inc.
(hereafter referred to as the Sponsor or MSD)

Legal Registered Address:

One Merck Drive

P.O. Box 100

Whitehouse Station, New Jersey, 08889-0100, U.S.A.

Regulatory Agency Identifying Number(s):

	NA

Approval Date: 19 April 2021

Sponsor Signatory

Typed Name: Date
Title:

Protocol-specific Sponsor contact information can be found in the Investigator Study File Binder (or equivalent).

Investigator Signatory

I agree to conduct this clinical study in accordance with the design outlined in this protocol and to abide by all provisions of this protocol.

Typed Name: Date
Title:

DOCUMENT HISTORY

Document	Date of Issue	Overall Rationale
3475-555-02	19-APR-2021	To update the dose modification and toxicity management guidelines for irAEs.
3475-555-01	28-SEP-2018	To add an additional cohort of 100 participants (Cohort B) who will receive 400 mg IV pembrolizumab Q6W from C1D1.
3475-555-00	06-JUN-2018	To estimate the relative bioavailability of an SC versus an IV dose of pembrolizumab, and to characterize the PK profile of pembrolizumab SC.

PROTOCOL AMENDMENT SUMMARY OF CHANGES

Amendment: 02

Overall Rationale for the Amendments:

To harmonize the presentation of safety information across all FDA-approved PD-1/L1 antibody prescribing information.

Summary of Changes Table:

Section # and Name	Description of Change	Brief Rationale
6.6.1 Immune-Related Events and Dose Modification (Withhold, Treat, Discontinue)	The dose modification and toxicity management guidelines for irAEs and table were updated.	The dose modification and toxicity management guidelines for irAEs and table were updated as requested by the U.S. FDA in an effort to harmonize the presentation of safety information across all FDA-approved PD-1/L1 antibody prescribing information.

Table of Contents

DOCUMENT HISTORY 3
PROTOCOL AMENDMENT SUMMARY OF CHANGES 4
1 PROTOCOL SUMMARY 13
1.1 Synopsis 13
1.2 Schema 19
1.3 Schedule of Activities (SoA) 20
1.3.1 Schedule of Activities in the Screening, Treatment, and Follow-up Periods 20
1.3.2 Pembrolizumab PK and ADA Sampling in the Treatment Period 31
2 INTRODUCTION 33
2.1 Study Rationale 33
2.2 Background 33
2.2.1 Pharmaceutical and Therapeutic Background 34
2.2.2 Pre-clinical and Clinical Studies 34
2.2.3 Ongoing Clinical Studies 35
2.2.4 Information on Other Study-related Therapy 35
2.3 Benefit/Risk Assessment 35
3 HYPOTHESIS, OBJECTIVES, AND ENDPOINTS 35
4 STUDY DESIGN 38
4.1 Overall Design 38
4.2 Scientific Rationale for Study Design 39
4.2.1 Rationale for Endpoints 39
4.2.1.1 Safety Endpoints 39
4.2.1.2 Pharmacokinetic Endpoints 39
4.2.1.3 Antidrug Antibodies (ADA) 40
4.2.1.4 Efficacy Endpoints 40
4.3 Justification for Dose 42
4.4 Beginning and End of Study Definition 43
4.4.1 Clinical Criteria for Early Study Termination 44
5 STUDY POPULATION 44
5.1 Inclusion Criteria 44
5.2 Exclusion Criteria 47
5.3 Lifestyle Considerations 49
5.3.1 Meals and Dietary Restrictions 49
5.3.2 Caffeine, Alcohol, and Tobacco Restrictions 49

5.3.3 Activity Restrictions ... 49
5.3.4 Contraception ... 49
5.3.5 Pregnancy ... 50
5.3.6 Use in Nursing Women ... 50
5.4 Screen Failures ... 50
5.5 Participant Replacement Strategy ... 50
6 STUDY INTERVENTION ... 51
6.1 Study Intervention(s) Administered ... 52
6.1.1 Medical Devices ... 53
6.2 Preparation/Handling/Storage/Accountability ... 53
6.2.1 Dose Preparation ... 53
6.2.2 Handling, Storage, and Accountability ... 53
6.3 Measures to Minimize Bias: Randomization and Blinding ... 54
6.3.1 Intervention Assignment ... 54
6.3.2 Stratification ... 55
6.3.3 Blinding ... 55
6.4 Study Intervention Compliance ... 55
6.5 Concomitant Therapy ... 55
6.5.1 Rescue Medications and Supportive Care ... 56
6.6 Dose Modification (Escalation/Titration/Other) ... 57
6.6.1 Immune-Related Events and Dose Modification (Withhold, Treat, Discontinue) ... 57
6.7 Intervention After the End of the Study ... 63
6.8 Clinical Supplies Disclosure ... 63
6.9 Standard Policies ... 63
7 DISCONTINUATION OF STUDY INTERVENTION AND PARTICIPANT WITHDRAWAL ... 64
7.1 Discontinuation of Study Intervention ... 64
7.2 Participant Withdrawal From the Study ... 65
7.3 Lost to Follow-up ... 65
8 STUDY ASSESSMENTS AND PROCEDURES ... 66
8.1 Administrative and General Procedures ... 66
8.1.1 Informed Consent ... 66
8.1.1.1 General Informed Consent ... 67
8.1.2 Inclusion/Exclusion Criteria ... 67
8.1.3 Participant Identification Card ... 67
8.1.4 Medical History ... 67
8.1.5 Prior and Concomitant Medications Review ... 68

8.1.5.1 Prior Medications 68
8.1.5.2 Concomitant Medications 68
8.1.6 Assignment of Screening Number 69
8.1.7 Assignment of Treatment/Randomization Number 69
8.1.8 Study Intervention Administration 69
8.1.8.1 Timing of Dose Administration 69
8.1.9 Discontinuation and Withdrawal 70
8.1.10 Participant Blinding/Unblinding 70
8.1.11 Domiciling 70
8.1.12 Calibration of Equipment 70
8.2 Efficacy/ Assessments 71
8.2.1 Tumor Imaging and Assessment of Disease 71
8.2.1.1 Initial Tumor Imaging 72
8.2.1.2 Tumor Imaging During the Study 72
8.2.1.3 End-of-Treatment and Follow-up Tumor Imaging 73
8.2.1.4 RECIST 1.1 Assessment of Disease 73
8.2.1.5 iRECIST Assessment of Disease 74
8.3 Safety Assessments 76
8.3.1 Adverse Events 77
8.3.2 Physical Examinations 77
8.3.2.1 Full Physical Examination 77
8.3.2.2 Directed Physical Examination 77
8.3.3 Vital Signs 77
8.3.4 Electrocardiograms 77
8.3.5 Clinical Safety Laboratory Assessments 78
8.3.5.1 Laboratory Safety Evaluations (Hematology, Chemistry and Urinalysis) 78
8.3.5.2 Pregnancy Test 79
8.3.6 Performance Assessments 79
8.3.6.1 Eastern Cooperative Oncology Group Performance Scale 79
8.3.7 Injection Site Signs and Symptoms Questionnaire 79
8.4 Adverse Events (AEs), Serious Adverse Events (SAEs), and Other Reportable Safety Events 79
8.4.1 Time Period and Frequency for Collecting AE, SAE, and Other Reportable Safety Event Information 80
8.4.2 Method of Detecting AEs, SAEs, and Other Reportable Safety Events 82
8.4.3 Follow-up of AE, SAE, and Other Reportable Safety Event Information 82
8.4.4 Regulatory Reporting Requirements for SAE 82

8.4.5 Pregnancy and Exposure During Breastfeeding 82
8.4.6 Disease-related Events and/or Disease-related Outcomes Not Qualifying as AEs or SAEs 83
8.4.7 Events of Clinical Interest (ECIs) 83
8.4.8 Medical Device Incidents (Including Malfunctions) 83
8.5 Treatment of Overdose 83
8.6 Pharmacokinetics 83
8.6.1 Blood Collection for Plasma for Pembrolizumab Pharmacokinetics and Immunogenicity 84
8.6.1.1 Blood Collection for Pharmacokinetics 84
8.6.1.2 Blood Collection for Anti-pembrolizumab Antibodies 84
8.7 Pharmacodynamics 84
8.8 Future Biomedical Research Sample Collection 84
8.9 Planned Genetic Analysis Sample Collection 84
8.10 Biomarkers 84
8.10.1 RNA Transcriptome Research 84
8.10.2 RNA Expression Research of a Subset of RNA Species 84
8.10.3 Proteome Research 85
8.10.4 Metabolomic Research 85
8.11 Visit Requirements 85
8.11.1 Screening 85
8.11.2 Treatment Period 85
8.11.3 Discontinued Participants Continuing to be Monitored in the Study 86
8.11.3.1 Follow-up Visits 86
8.11.3.2 Safety Follow-up Visit 86
8.11.3.3 Survival Follow-up 87
8.11.4 Survival Status 87
9 STATISTICAL ANALYSIS PLAN 87
9.1 Statistical Analysis Plan Summary 87
9.2 Responsibility for Analyses/In-house Blinding 90
9.3 Hypotheses/Estimation 90
9.4 Analysis Endpoints 90
9.4.1 Pharmacokinetics Endpoints (Cohorts A and B) 90
9.4.2 Safety Endpoints (Cohorts A and B) 90
9.4.3 Efficacy Endpoints (Cohort B) 91
9.4.3.1 Primary 91
9.4.3.2 Secondary 91
9.5 Analysis Populations 91

9.5.1 Pharmacokinetic Analysis Population (Cohorts A and B) ... 91
9.5.2 Safety Analysis Population (Cohorts A and B) ... 92
9.5.3 Efficacy Analysis Population (Cohort B) ... 92
9.6 Statistical Methods ... 92
9.6.1 Statistical Methods for Pharmacokinetic and Antidrug Antibodies Data (Cohorts A and B) ... 92
9.6.2 Statistical Methods for Efficacy Analyses (Cohort B) ... 93
9.6.2.1 Objective Response Rate (ORR) ... 93
9.6.2.2 Progression-free Survival (PFS) ... 94
9.6.2.3 Overall Survival (OS) ... 94
9.6.2.4 Duration of Response (DOR) ... 95
9.6.2.5 Analysis Strategy for Key Efficacy Endpoint ... 95
9.6.3 Statistical Methods for Safety Analysis (Cohort A) ... 96
9.6.4 Statistical Methods for Safety Analyses (Cohort B) ... 97
9.6.5 Demographics and Baseline Characteristics (Cohort B) ... 97
9.7 Interim Analyses ... 97
9.8 Multiplicity ... 98
9.9 Sample Size and Power Calculations ... 98
9.10 Subgroup Analyses ... 98
9.11 Compliance (Medication Adherence) ... 99
9.12 Extent of Exposure ... 99
10 SUPPORTING DOCUMENTATION AND OPERATIONAL CONSIDERATIONS ... 100
10.1 Appendix 1: Regulatory, Ethical, and Study Oversight Considerations ... 100
10.1.1 Code of Conduct for Clinical Trials ... 100
10.1.2 Financial Disclosure ... 102
10.1.3 Data Protection ... 102
10.1.3.1 Confidentiality of Data ... 103
10.1.3.2 Confidentiality of Participant Records ... 103
10.1.3.3 Confidentiality of IRB/IEC Information ... 103
10.1.4 Committees Structure ... 103
10.1.5 Publication Policy ... 103
10.1.6 Compliance with Study Registration and Results Posting Requirements ... 104
10.1.7 Compliance with Law, Audit, and Debarment ... 104
10.1.8 Data Quality Assurance ... 105
10.1.9 Source Documents ... 106
10.1.10 Study and Site Closure ... 106
10.2 Appendix 2: Clinical Laboratory Tests ... 107

10.3 Appendix 3: Adverse Events: Definitions and Procedures for Recording, Evaluating, Follow-up, and Reporting......109
10.3.1 Definition of AE......109
10.3.2 Definition of SAE......110
10.3.3 Additional Events Reported in the Same Manner as SAE......111
10.3.4 Recording AE and SAE......112
10.3.5 Reporting of AE, SAE, and Other Reportable Safety Events to the Sponsor......114
10.4 Appendix 4: Medical Device Incidents: Definition and Procedures for Recording, Evaluating, Follow-up, and Reporting......116
10.5 Appendix 5: Contraceptive Guidance and Pregnancy Testing......117
10.5.1 Definitions......117
10.5.2 Contraception Requirements......118
10.5.3 Pregnancy Testing......119
10.6 Appendix 6: Collection and Management of Specimens for Future Biomedical Research......120
10.7 Appendix 7: Country-specific Requirements......121
10.8 Appendix 8: Description of the iRECIST Process for Assessment of Disease Progression......122
10.9 Appendix 9: Abbreviations......126
11 REFERENCES......129

LIST OF TABLES

Table 1 Schedule of Activities Cohort A 20
Table 2 Schedule of Activities Cohort B 25
Table 3 Adequate Organ Function Laboratory Values 46
Table 4 Study Interventions 52
Table 5 Sample Allocation Schedule (Cohort A) 54
Table 6 Dose Modification and Toxicity Management Guidelines for Immune-related Adverse Events Associated with Pembrolizumab Monotherapy, Coformulations or IO Combinations 58
Table 7 Pembrolizumab Infusion/Injection Reaction Dose Modification and Treatment Guidelines 62
Table 8 Imaging and Treatment after First Radiologic Evidence of Progressive Disease 75
Table 9 Reporting Time Periods and Time Frames for Adverse Events and Other Reportable Safety Events 81
Table 10 Censoring Rules for Analysis of Progression-free Survival 94
Table 11 Censoring Rules for Duration of Response 95
Table 12 Analysis Strategy for Key Efficacy Endpoints 96
Table 13 Analysis Strategy for Safety Parameters 97
Table 14 Protocol-required Safety Laboratory Assessments 108
Table 15 Highly Effective Contraception Methods 118

LIST OF FIGURES

Figure 1 Trial Design Diagram 19
Figure 2 Imaging and Treatment for Clinically Stable Participants Treated With Pembrolizumab After First Radiologic Evidence of PD Assessed by the Investigators 76

1 PROTOCOL SUMMARY

1.1 Synopsis

Protocol Title: A Phase 1 Randomized Clinical Study of Pembrolizumab (MK-3475) to Evaluate the Relative Bioavailability of Subcutaneous Injection Versus Intravenous Infusion in Participants with Advanced Melanoma (KEYNOTE-555)

Short Title: Relative Bioavailability Study of Subcutaneous Injection Versus Intravenous Infusion of MK-3475/Pembrolizumab in Participants with Advanced Melanoma

Acronym: Not applicable.

Hypotheses, Objectives, and Endpoints:

Male/female participants of at least 18 years of age with advanced melanoma will be enrolled in this study.

Primary Objectives	Primary Endpoints
Cohort A<br><br>- To characterize the pharmacokinetic (PK) profile, including the absorption phase, of pembrolizumab following subcutaneous (SC) injection [redacted]<br><br>- To estimate the relative bioavailability of pembrolizumab via SC injection [redacted] versus intravenous (IV) infusion (200 mg: 25 mg/mL)	- Pharmacokinetic parameters of pembrolizumab, including area under the curve (AUC), maximum concentration (Cmax), bioavailability (F), absorption rate (Ka), time of maximum concentration (Tmax), clearance (CL), and central volume of distribution (Vc)
Primary Objectives	Primary Endpoints
Cohort B<br>- To evaluate objective response rate (ORR) per RECIST 1.1 as assessed by blinded independent central review (BICR)	- Participants who have a confirmed CR or PR

Secondary Objectives	Secondary Endpoints
Cohort A<br>- To evaluate the development of circulating anti-pembrolizumab antibodies (if applicable), following SC pembrolizumab	- Antidrug antibody levels
- To determine the safety and tolerability of SC pembrolizumab during the first 3 treatment cycles based on the proportion of adverse events (AEs)	- Adverse events (AEs)<br>- Study drug discontinuations due to AEs
- To monitor the safety and tolerability of IV pembrolizumab during the first 3 treatment cycles and after Cycle 3 based on the proportion of AEs	- Adverse events (AEs)<br>- Study drug discontinuations due to AEs
- To determine the tolerability of SC pembrolizumab at the injection site during the first 3 treatment cycles based on the proportion of injection site signs and symptoms	- Responses to an injection site signs and symptoms questionnaire
Secondary Objectives	Secondary Endpoints
Cohort B<br>- To evaluate duration of response (DOR) per RECIST 1.1 as assessed by BICR	- For participants who demonstrate CR or PR, DOR is defined as the time from first documented evidence of CR or PR until disease progression or death due to any cause, whichever occurs first
- To evaluate progression-free survival (PFS) as assessed by BICR according to RECIST v1.1, modified to follow a maximum of 10 target lesions and a maximum of 5 target lesions per organ	- PFS is the time from the first dose of study intervention to the first documented disease progression or death due to any cause, whichever occurs first
- To evaluate overall survival (OS)	- OS is defined as the time from the first day of study treatment to death due to any cause
- To characterize the PK profile of pembrolizumab following IV infusion Q6W	- Pharmacokinetic parameters of pembrolizumab, including AUC, Cmax, and Cmin
- To monitor the safety and tolerability of IV pembrolizumab Q6W based on the proportion of AEs	- Adverse events (AEs)<br>- Study drug discontinuations due to AEs

Exploratory Objectives	Exploratory Endpoints
Cohort B<br>- To evaluate the development of circulating anti-pembrolizumab antibodies (if applicable), following IV pembrolizumab Q6W	- Antidrug antibody levels

Overall Design:

Study Phase	Phase 1
Primary Purpose	Treatment
Indication	Treatment of participants with advanced melanoma
Population	Male/female participants with advanced melanoma who are at least 18 years of age on the day of signing consent.
Study Type	Interventional
Intervention Model	Cross-over<br>This is a multi-site study.
Type of Control	Active Controlled Study
Study Blinding	Unblinded Open-label
Masking	No Masking
Estimated Duration of Study	The Sponsor estimates that the study will require approximately 141 weeks from the time the first participant signs the informed consent until the last participant's last study-related telephone call or visit.

Number of Participants:

Approximately 36 participants will be allocated/randomized in Cohort A such that 30 evaluable participants complete the study, and approximately 100 participants will be allocated in Cohort B as described in Section 9.9.

Enrollment into Cohort A takes priority over Cohort B.

Intervention Groups and Duration:

Intervention Groups

Intervention Arm	Drug	Concentration/ Total mg	Dose Frequency	Route of Admin.	Regimen/ Treatment Period	Use
**Cohort A**						
Arm 1	Pembrolizumab	(B) CCI [redacted]	Single administration	SC	Cycle 1	Experimental
	Pembrolizumab	(C) 25 mg/mL/ 200 mg	Single administration	IV	Cycle 2	Experimental
	Pembrolizumab	(A) CCI [redacted]	Single administration	SC	Cycle 3	Experimental
	Pembrolizumab	(D) 25 mg/mL/ 200 mg	Q3W	IV	Day 1 of each subsequent 21-day cycle	Experimental
Arm 2	Pembrolizumab	(B) CCI [redacted]	Single administration	SC	Cycle 1	Experimental
	Pembrolizumab	(A) CCI [redacted]	Single administration	SC	Cycle 2	Experimental
	Pembrolizumab	(C) 25 mg/mL/ 200 mg	Single administration	IV	Cycle 3	Experimental
	Pembrolizumab	(D) 25 mg/mL/ 200 mg	Q3W	IV	Day 1 of each subsequent 21-day cycle	Experimental
Arm 3	Pembrolizumab	(A) CCI [redacted]	Single administration	SC	Cycle 1	Experimental
	Pembrolizumab	(B) CCI [redacted]	Single administration	SC	Cycle 2	Experimental
	Pembrolizumab	(C) 25 mg/mL/ 200 mg	Single administration	IV	Cycle 3	Experimental
	Pembrolizumab	(D) 25 mg/mL/ 200 mg	Q3W	IV	Day 1 of each subsequent 21-day cycle	Experimental

Arm 4	Pembrolizumab	(A) CCI [redacted]	Single administration	SC	Cycle 1	Experimental
	Pembrolizumab	(C) 25 mg/mL/ 200 mg	Single administration	IV	Cycle 2	Experimental
	Pembrolizumab	(B) CCI [redacted]	Single administration	SC	Cycle 3	Experimental
	Pembrolizumab	(D) 25 mg/mL/ 200 mg	Q3W	IV	Day 1 of each subsequent 21-day cycle	Experimental
Arm 5	Pembrolizumab	(C) 25 mg/mL/ 200 mg	Single administration	IV	Cycle 1	Experimental
	Pembrolizumab	(A) CCI [redacted]	Single administration	SC	Cycle 2	Experimental
	Pembrolizumab	(B) CCI [redacted]	Single administration	SC	Cycle 3	Experimental
	Pembrolizumab	(D) 25 mg/mL/ 200 mg	Q3W	IV	Day 1 of each subsequent 21-day cycle	Experimental
Arm 6	Pembrolizumab	(C) 25 mg/mL/ 200 mg	Single administration	IV	Cycle 1	Experimental
	Pembrolizumab	(B) CCI [redacted]	Single administration	SC	Cycle 2	Experimental
	Pembrolizumab	(A) CCI [redacted]	Single administration	SC	Cycle 3	Experimental
	Pembrolizumab	(D) 25 mg/mL/ 200 mg	Q3W	IV	Day 1 of each subsequent 21-day cycle	Experimental
**Cohort B**						
Arm 7	Pembrolizumab	25 mg/mL/ 400 mg	Q6W	IV	Day 1 of each 42-day cycle	Experimental
IV=intravenous; Q3W=every 3 weeks; Q6W=every 6 weeks; SC=subcutaneous.						

**Total Number**	Cohort A: 6 Arms<br>Cohort B: 1 Arm
Duration of Participation	Each participant will participate in the study from the time the participant signs the Informed Consent Form through the final protocol-specified contact.<br><br>After a screening phase of up to 28 days, each participant will be assigned to receive study treatment until disease progression is radiographically documented, confirmed by the site per modified Response Evaluation Criteria in Solid Tumors 1.1 (iRECIST) when clinically appropriate, unacceptable AEs, intercurrent illness that prevents further administration of treatment, investigator's decision to discontinue the participant, noncompliance with study treatment or procedure requirements or administrative reasons requiring cessation of treatment, or until the participant has received 35 administrations (Cohort A) or 18 administrations (Cohort B) of pembrolizumab (approximately 2 years).<br><br>After the end of treatment, each participant will be followed for the occurrence of AEs and spontaneously reported pregnancy as described under Section 8.4.<br><br>In Cohort B participants who discontinue for reasons other than radiographic disease progression will have post-treatment follow-up imaging for disease status until disease progression is documented radiographically per RECIST 1.1, and, when clinically appropriate, confirmed by the site per iRECIST, the start of a new anti-cancer treatment, withdrawal of consent, pregnancy, death, or lost to follow-up. All participants will be followed by telephone for overall survival until death, withdrawal of consent, or the end of the study.<br><br>Upon study completion, participants may be enrolled in a pembrolizumab extension study if available.

Study Governance Committees:

Steering Committee	No
Executive Oversight Committee	No
Data Monitoring Committee	No
Clinical Adjudication Committee	No
Study governance considerations are outlined in Appendix 1.	

Study Accepts Healthy Volunteers: No

A list of abbreviations used in this document can be found in Appendix 9.

1.2 Schema

The study design is depicted in Figure 1.

Cohort A

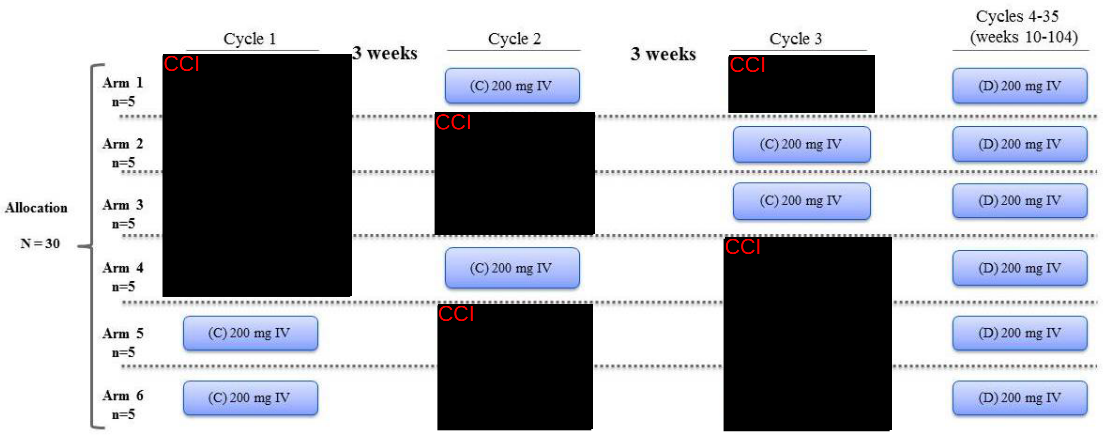

Cohort B

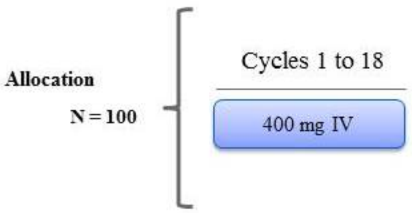

Figure 1 Trial Design Diagram

Note: Cohort A: A [redacted] SC; B [redacted] SC; C=200 mg IV in Cycle 1 to Cycle 3; and D=200 mg IV in Cycle 4 to Cycle 35. Cohort B: participants will receive pembrolizumab 400 mg IV Q6W.

Abbreviations: IV=intravenous; SC=subcutaneous.

1.3 Schedule of Activities (SoA)

1.3.1 Schedule of Activities in the Screening, Treatment, and Follow-up Periods

Table 1 Schedule of Activities Cohort A

**Study Period**	**Screening**	**Cohort A Treatment Period (Cycle = 21 days)**				**End of Treatment / Discon-tinuation**	**Posttreatment Period**		**Notes**
**Treatment Cycle/Title**	**Screening**	**Cycle 1**	**Cycle 2**	**Cycle 3**	**Cycles ≥4 (up to 35 cycles)**	**At the time of treatment discon-tinuation**	**Safety Follow-up (90-day follow-up by phone contact only)**		
**Treatment Day per Cycle**		**Day 1**	**Day 1**	**Day 1**	**Day 1**		**30 days after the last dose**	**90 days after the last dose**	
**Visit Window (Days)**	**-28 to -1**	**± 0[a]**	**± 0[a]**	**± 0[a]**	**± 3[a]**	**+ 7**	**+ 7**		Individual procedure windows may vary
**Administrative Procedures**									
Informed Consent	X								Written informed consent must be obtained prior to performing any protocol-specific procedures. Tests performed prior to consent as part of routine clinical management are acceptable if performed within the specified timeframe.
Inclusion/Exclusion Criteria	X								
Participant Identification Card	X								
Demographics and Medical History	X								
Oncology Disease Status and Prior Oncology Treatment History	X								
Concomitant Medication	X	X	X	X	X	X	X	X	Concomitant medications will be collected during the 90-day safety follow-up if the medications are related to an SAE.
Treatment Randomization		X							Dose within 3 days of randomization
Pembrolizumab Administration		X	X	X	X				

Study Period	Screening	Cohort A Treatment Period (Cycle = 21 days)				End of Treatment / Discon-tinuation	Posttreatment Period		Notes
**Treatment Cycle/Title**	**Screening**	**Cycle 1**	**Cycle 2**	**Cycle 3**	**Cycles ≥4 (up to 35 cycles)**	**At the time of treatment discon-tinuation**	**Safety Follow-up (90-day follow-up by phone contact only)**		
**Treatment Day per Cycle**		**Day 1**	**Day 1**	**Day 1**	**Day 1**		**30 days after the last dose**	**90 days after the last dose**	
**Visit Window (Days)**	**-28 to -1**	**± 0[a]**	**± 0[a]**	**± 0[a]**	**± 3[a]**	**+ 7**	**+ 7**		Individual procedure windows may vary
**Clinical Procedures/Assessments**									
Tumor Imaging, RECIST 1.1, and iRECIST Response Assessment	X	←					→		Baseline tumor imaging should be performed within 28 days of enrollment. First post-baseline assessment should be performed at approximately Week 12, and then per institutional standard of care. Response status will be assessed by the study site. Continue imaging until disease progression, discontinuation, or End of Treatment.
Full Physical Examination	X		X			X	X		For Cycles 4 to 35, directed physical examinations may be performed unless a full examination is deemed necessary.
Directed Physical Examination		X		X	X				
Height	X								Perform at screening only.
Weight	X	X	X	X	X	X	X		
Vital Signs	X	X	X	X	X	X	X		To be measured at screening, and within 1 h (± 30 min) prior to dosing (SC and IV), and approximately 0.5 hours after pembrolizumab administration (SC only). Measurements include temperature, pulse, respiratory rate, and blood pressure.
ECOG Performance Status	X								ECOG will be obtained at screening only (within 7 days of the first dose). Additional ECOG assessments may be performed as clinically indicated.

Study Period	Screening	Cohort A Treatment Period (Cycle = 21 days)				End of Treatment / Discon-tinuation	Posttreatment Period		Notes
Treatment Cycle/Title	Screening	Cycle 1	Cycle 2	Cycle 3	Cycles ≥4 (up to 35 cycles)	At the time of treatment discon-tinuation	Safety Follow-up (90-day follow-up by phone contact only)		
Treatment Day per Cycle		Day 1	Day 1	Day 1	Day 1		30 days after the last dose	90 days after the last dose	
Visit Window (Days)	-28 to -1	± 0[a]	± 0[a]	± 0[a]	± 3[a]	+ 7	+ 7		Individual procedure windows may vary
12-Lead Electrocardiogram	X								ECG will be performed at screening only. Additional ECG assessments may be performed as clinically indicated.
Adverse Event Monitoring	X	←——————————————————————→							All AEs that occur after the consent form is signed but before treatment allocation must be reported by the investigator if the event causes the participant to be excluded from the study or is the result of a protocol-specified intervention. There is to be continuous AE reporting from the time of treatment allocation. After treatment discontinuation, participants will be monitored for AEs for 30 days and SAEs for 90 days (30 days if the participant initiates new anticancer therapy). Participants with an ongoing AE at the time of treatment discontinuation will be followed until resolution, stabilization, the event is otherwise explained, or the participant is lost to follow-up.
**Laboratory Procedures/Assessments**									
CBC with Differential	X	X	X	X	X	X	X		Perform all screening clinical laboratory tests within 72 hours of treatment initiation. Perform all subsequent scheduled clinical laboratory tests within 72 hours
Chemistry Panel	X	X	X	X	X	X	X		
LDH	X								
Urinalysis	X	X	X	X	X	X	X		

Study Period	Screening	Cohort A Treatment Period (Cycle = 21 days)				End of Treatment / Discon-tinuation	Posttreatment Period		Notes
Treatment Cycle/Title	Screening	Cycle 1	Cycle 2	Cycle 3	Cycles ≥4 (up to 35 cycles)	At the time of treatment discon-tinuation	Safety Follow-up (90-day follow-up by phone contact only)		
Treatment Day per Cycle		Day 1	Day 1	Day 1	Day 1		30 days after the last dose	90 days after the last dose	
Visit Window (Days)	-28 to -1	± 0[a]	± 0[a]	± 0[a]	± 3[a]	+ 7	+ 7		Individual procedure windows may vary
									prior to treatment.
Thyroid Function Testing (T4 or FT4, T3 or FT3, TSH)	X		X		X				Thyroid function: To be collected at screening (within 28 days prior to treatment) and then at every other cycle (ie. Cycle 2, 4, 6, etc.). Total T4 and T3 are preferred over FT4 and FT3.
Pregnancy test for WOCBP only (urine or serum β-hCG)	X	X							Perform within 72 hours prior to C1D1 (if collection is done within this timeframe do not need to repeat for the C1D1). Urine pregnancy test to be performed as indicated; if test is positive or cannot be confirmed as negative, a serum pregnancy test is required. Additional pregnancy testing may be conducted if required by local regulations or if clinically indicated.
HIV/Hepatitis B and C Screen (per site SOP)	X								To be collected at screening (within 28 days prior to treatment). Include HCV antibody or HCV RNA (qualitative) and HBsAg. HIV, Hepatitis B and C by history are acceptable for exclusion, unless testing is required by local regulations.
PK for IV and SC pembrolizumab		X[a]	X[a]	X[a]	X[a]				Cycles 1 to 4 only; refer to Section 1.3.2 for full details.
Anti-pembrolizumab Antibody		X[a]	X[a]	X[a]	X[a]				Refer to Section 1.3.2 for full details.

**Study Period**	**Screening**	**Cohort A Treatment Period (Cycle = 21 days)**				**End of Treatment / Discon-tinuation**	**Posttreatment Period**		**Notes**
**Treatment Cycle/Title**	**Screening**	**Cycle 1**	**Cycle 2**	**Cycle 3**	**Cycles ≥4 (up to 35 cycles)**	**At the time of treatment discon-tinuation**	**Safety Follow-up (90-day follow-up by phone contact only)**		
**Treatment Day per Cycle**		**Day 1**	**Day 1**	**Day 1**	**Day 1**		**30 days after the last dose**	**90 days after the last dose**	
**Visit Window (Days)**	**-28 to -1**	**± 0[a]**	**± 0[a]**	**± 0[a]**	**± 3[a]**	**+ 7**	**+ 7**		Individual procedure windows may vary
Injection Site Signs and Symptoms Questionnaire		←	—	→					Tolerability and injection difficulty will be assessed at Day 1 of each cycle of pembrolizumab administration (SC only), 60 minutes postdose.

[a]Note that visit windows do not apply to the PK and ADA sampling.

Abbreviations: β-hCG=beta-human chorionic gonadotropin; AE=adverse event; C=cycle; CBC=complete blood count; D=day; ECG=electrocardiogram; ECOG=Eastern Cooperative Oncology Group; HCV=hepatitis c virus; HIV=human immunodeficiency virus; iRECIST= modified Response Evaluation Criteria in Solid Tumors; IV=intravenous; LDH=lactase dehydrogenase; PK=pharmacokinetics; RECIST=Response Evaluation Criteria in Solid Tumors; RNA=ribonucleic acid; SAE=serious adverse event; SC=subcutaneous; SOP=standard operating procedure; TSH=thyroid stimulating hormone; WOCBP=woman/women of childbearing potential.

Table 2 Schedule of Activities Cohort B

**Study Period**	**Screening**	**Cohort B Treatment Period (Cycle=42 days)**				**Cohort B Treatment Period (Cycle= 42 days)**	**End of Treatment / Discon-tinuation**	**Posttreatment Period**			**Notes**
**Treatment Cycle/Title**	**Screening**	**Cycle 1**		**Cycle 4**		**Cycles 2 to 3 and Cycles 5 to 18**	**At the time of treatment discon-tinuation**	**Safety Follow-up**	**Imaging Follow-up**	**Survival Follow-up**	
**Treatment Day per Cycle**		**Day 1**	**Day 22**	**Day 1**	**Day 22**	**Day 1 + Day 22**		**30 days after the last dose**	**Every 12 weeks**	**Every 12 weeks**	
**Visit Window (Days)**	**-28 to -1**	**± 0[a]**	**± 3[a]**	**± 3[a]**	**± 3[a]**	**± 3[a]**	**+ 7**	**+ 7**	**± 7**	**± 7**	Individual procedure windows may vary
**Administrative Procedures**											
Informed Consent	X										Written informed consent must be obtained prior to performing any protocol-specific procedures. Tests performed prior to consent as part of routine clinical management are acceptable if performed within the specified timeframe.
Inclusion/Exclusion Criteria	X										
Participant Identification Card	X										
Demographics and Medical History	X										
Oncology Disease Status and Prior Oncology Treatment History	X										
Concomitant Medication	X	X	X	X	X	X	X	X			Concomitant medications will be collected for 30 days after the last dose then only those related to an SAE/ECI.
Treatment Randomization		X									Dose within 3 days of randomization
Pembrolizumab Administration		X		X		X					

Study Period	Screening	Cohort B Treatment Period (Cycle=42 days)				Cohort B Treatment Period (Cycle= 42 days)	End of Treatment / Discon-tinuation	Posttreatment Period			Notes
**Treatment Cycle/Title**	**Screening**	**Cycle 1**		**Cycle 4**		**Cycles 2 to 3 and Cycles 5 to 18**	**At the time of treatment discon-tinuation**	**Safety Follow-up**	**Imaging Follow-up**	**Survival Follow-up**	
**Treatment Day per Cycle**		**Day 1**	**Day 22**	**Day 1**	**Day 22**	**Day 1 + Day 22**		**30 days after the last dose**	**Every 12 weeks**	**Every 12 weeks**	
**Visit Window (Days)**	**-28 to -1**	**± 0[a]**	**± 3[a]**	**± 3[a]**	**± 3[a]**	**± 3[a]**	**+ 7**	**+ 7**	**± 7**	**± 7**	Individual procedure windows may vary
**Clinical Procedures/Assessments**											
Tumor Imaging, RECIST 1.1, and iRECIST Response Assessment	X	←――――――――――――――――――――→							X		Baseline tumor imaging should be performed within 28 days of first dose. First post-baseline assessment should be performed at approximately Week 12. Assessments should then be performed every 9 weeks (± 7 days) in Year 1 and 12 weeks after Year 1 calculated from day of first dose. Imaging should be performed at the time of treatment discontinuation (ie, date of discontinuation ± 4 weeks). If previous scan was obtained within 4 weeks prior to the date of discontinuation, then a scan at treatment discontinuation does not need to be performed. Response status will be assessed by the study site. Continue imaging until disease progression, the start of new anti-cancer treatment, withdrawal of consent, or death, whichever occurs first.

**Study Period**	**Screening**	**Cohort B Treatment Period (Cycle=42 days)**				**Cohort B Treatment Period (Cycle= 42 days)**	**End of Treatment / Discon-tinuation**	**Posttreatment Period**			**Notes**
**Treatment Cycle/Title**	**Screening**	**Cycle 1**		**Cycle 4**		**Cycles 2 to 3 and Cycles 5 to 18**	**At the time of treatment discon-tinuation**	**Safety Follow-up**	**Imaging Follow-up**	**Survival Follow-up**	
**Treatment Day per Cycle**		**Day 1**	**Day 22**	**Day 1**	**Day 22**	**Day 1 + Day 22**		**30 days after the last dose**	**Every 12 weeks**	**Every 12 weeks**	
**Visit Window (Days)**	**-28 to -1**	**± 0[a]**	**± 3[a]**	**± 3[a]**	**± 3[a]**	**± 3[a]**	**+ 7**	**+ 7**	**± 7**	**± 7**	Individual procedure windows may vary
Survival Status		←→								X	After investigator determined PD or start of new anticancer treatment. In addition, upon Sponsor request, participants may be contacted for survival status at any time during the course of the study.
Full Physical Examination	X	X					X	X			For Cycles 2 to 18, directed physical examinations may be performed unless a full examination is deemed necessary.
Directed Physical Examination			X	X	X	X					
Height	X										Perform at screening only.
Weight	X	X	X	X	X	X	X	X			
Vital Signs	X	X	X	X	X	X	X	X			Measurements include temperature, pulse, respiratory rate, and blood pressure.
ECOG Performance Status	X	X	X	X	X	X	X	X			ECOG will be obtained within 7 days of the first dose.
12-Lead Electrocardiogram	X										ECG will be performed at screening only. Additional ECG assessments may be performed as clinically indicated.

**Study Period**	**Screening**	**Cohort B Treatment Period (Cycle=42 days)**				**Cohort B Treatment Period (Cycle= 42 days)**	**End of Treatment / Discon-tinuation**	**Posttreatment Period**			**Notes**
**Treatment Cycle/Title**	**Screening**	**Cycle 1**		**Cycle 4**		**Cycles 2 to 3 and Cycles 5 to 18**	**At the time of treatment discon-tinuation**	**Safety Follow-up**	**Imaging Follow-up**	**Survival Follow-up**	
**Treatment Day per Cycle**		**Day 1**	**Day 22**	**Day 1**	**Day 22**	**Day 1 + Day 22**		**30 days after the last dose**	**Every 12 weeks**	**Every 12 weeks**	
**Visit Window (Days)**	**-28 to -1**	**± 0[a]**	**± 3[a]**	**± 3[a]**	**± 3[a]**	**± 3[a]**	**+ 7**	**+ 7**	**± 7**	**± 7**	Individual procedure windows may vary
Adverse Event Monitoring	X	←→	←→	←→	←→	←→	←→	←→	←→		All AEs that occur after the consent form is signed but before treatment allocation must be reported by the investigator if the event causes the participant to be excluded from the study or is the result of a protocol-specified intervention. There is to be continuous AE reporting from the time of treatment allocation. After treatment discontinuation, participants will be monitored for AEs for 30 days and SAEs for 90 days (30 days if the participant initiates new anticancer therapy). Participants with an ongoing AE at the time of treatment discontinuation will be followed until resolution, stabilization, the event is otherwise explained, or the participant is lost to follow-up.
**Laboratory Procedures/Assessments**											
CBC with Differential	X	X	X	X	X	X	X	X			Perform screening clinical laboratory tests within 72 hours of treatment initiation. Perform all subsequent scheduled
Chemistry Panel	X	X	X	X	X	X	X	X			
LDH	X										
Urinalysis	X	X	X	X	X	X	X	X			

**Study Period**	**Screening**	**Cohort B Treatment Period (Cycle=42 days)**				**Cohort B Treatment Period (Cycle= 42 days)**	**End of Treatment / Discon-tinuation**	**Posttreatment Period**			**Notes**
**Treatment Cycle/Title**	**Screening**	**Cycle 1**		**Cycle 4**		**Cycles 2 to 3 and Cycles 5 to 18**	**At the time of treatment discon-tinuation**	**Safety Follow-up**	**Imaging Follow-up**	**Survival Follow-up**	
**Treatment Day per Cycle**		**Day 1**	**Day 22**	**Day 1**	**Day 22**	**Day 1 + Day 22**		**30 days after the last dose**	**Every 12 weeks**	**Every 12 weeks**	
**Visit Window (Days)**	**-28 to -1**	**± 0[a]**	**± 3[a]**	**± 3[a]**	**± 3[a]**	**± 3[a]**	**+ 7**	**+ 7**	**± 7**	**± 7**	Individual procedure windows may vary
Thyroid Function Testing (T4 or FT4, T3 or FT3, TSH)	X			X		X	X				clinical laboratory tests within 72 hours prior to treatment.<br><br>Thyroid function: To be collected at screening (within 28 days prior to treatment) and then at every other cycle (ie. Cycle 2, 4, 6, etc.). Total T4 and T3 are preferred over FT4 and FT3.
Pregnancy test for WOCBP only (urine or serum β-hCG)	X	X									Perform within 72 hours prior to C1D1 (if collection is done within this timeframe do not need to repeat for the C1D1). Urine pregnancy test to be performed as indicated; if test is positive or cannot be confirmed as negative, a serum pregnancy test is required. Additional pregnancy testing may be conducted if required by local regulations or if clinically indicated.
HIV/Hepatitis B and C Screen (per site SOP)	X										To be collected at screening (within 28 days prior to treatment). Include HCV antibody or HCV RNA (qualitative) and HBsAg. HIV, Hepatitis B and C by history are acceptable for exclusion, unless testing is required by local regulations.

**Study Period**	**Screening**	**Cohort B Treatment Period (Cycle=42 days)**				**Cohort B Treatment Period (Cycle= 42 days)**	**End of Treatment / Discon-tinuation**	**Posttreatment Period**			**Notes**
**Treatment Cycle/Title**	**Screening**	**Cycle 1**		**Cycle 4**		**Cycles 2 to 3 and Cycles 5 to 18**	**At the time of treatment discon-tinuation**	**Safety Follow-up**	**Imaging Follow-up**	**Survival Follow-up**	
**Treatment Day per Cycle**		**Day 1**	**Day 22**	**Day 1**	**Day 22**	**Day 1 + Day 22**		**30 days after the last dose**	**Every 12 weeks**	**Every 12 weeks**	
**Visit Window (Days)**	**-28 to -1**	**± 0[a]**	**± 3[a]**	**± 3[a]**	**± 3[a]**	**± 3[a]**	**+ 7**	**+ 7**	**± 7**	**± 7**	Individual procedure windows may vary
PK for IV pembrolizumab		X[a]	X[a]	X[a]	X[a]						Refer to Section 1.3.2 for full details.
Anti-pembrolizumab Antibody		X[a]	X[a]	X[a]	X[a]						Refer to Section 1.3.2 for full details.

[a]Note that visit windows do not apply to the PK and ADA sampling.
Abbreviations: β-hCG=beta-human chorionic gonadotropin; AE=adverse event; C=cycle; CBC=complete blood count; D=day; ECG=electrocardiogram; ECOG=Eastern Cooperative Oncology Group; HCV=hepatitis c virus; HIV=human immunodeficiency virus; iRECIST= modified Response Evaluation Criteria in Solid Tumors; IV=intravenous; LDH=lactase dehydrogenase; PK=pharmacokinetics; RECIST=Response Evaluation Criteria in Solid Tumors; RNA=ribonucleic acid; SAE=serious adverse event; SOP=standard operating procedure; TSH=thyroid stimulating hormone; WOCBP=woman/women of childbearing potential.

1.3.2 Pembrolizumab PK and ADA Sampling in the Treatment Period

Study Period	Cohort A<br>Treatment Period<br>Cycle = 21 days											
**Treatment Cycle/Title**	**Cycles 1, 2, and/or 3, According to Arm**										**Cycle 4**	
**Treatment Day per Cycle**	**1**	**1 (post-infusion)**	**2**	**3**	**4**	**5**	**6**	**7**	**10**	**15**	**1**	**Notes**
Anti-pembrolizumab Antibody (Arms 1 to 6)	X										X	Collect samples **predose** 0-4 h before pembrolizumab on C1D1, C2D1, C3D1, and C4D1. Collect with plasma samples for pembrolizumab PK when feasible.
**Pembrolizumab (SC Dosing)**												
Pharmacokinetics for SC Pembrolizumab	X		X	X	X	X	X	X	X	X	X	Collect **predose** samples 0-4 h before pembrolizumab administration on C1D1, C2D1, C3D1, and C4D1. Samples collected on D2, D3, D4, D5, D6, D7, D10, and D15 can be collected anytime that day.
**Pembrolizumab (First Cycle of IV Dosing)**												
Pharmacokinetics for IV Pembrolizumab	X	X	X			X			X	X	X	Collect **predose** samples 0-4 h before pembrolizumab administration on C1D1, C2D1, C3D1, and C4D1 and at the end of infusion (as soon as possible, but within 10 minutes) on C1D1, C2D1, and C3D1. Samples collected on D2, D5, D10, and D15 can be collected anytime that day.

Note: If the sample day falls on a weekend or holiday and the sample cannot be drawn, an alternate sampling day will not occur.
Abbreviations: C=Cycle; D=Day; IV=intravenous; PK=pharmacokinetics; SC=subcutaneous.

**Study Period**	**Cohort B Treatment Period Cycle= 42 days**												**Notes**
**Treatment Cycle/Title**	**1**			**2**			**4**			**5**			
**Treatment Day per Cycle**	**1**	**1 (post-infusion)**	**22**	**1**	**1 (post-infusion)**	**22**	**1**	**1 (post-infusion)**	**22**	**1**	**1 (post-infusion)**	**22**	
Anti-pembrolizumab Antibody	**X**	**X**	**X**	**X**			**X**	**X**	**X**	**X**			Collect **predose** samples 0-4 h before pembrolizumab administration on C1D1, C2D1, C4D1, and C5D1, and at the end of infusion (as soon as possible, but within 5 minutes) on C1D1 and C4D1. Samples collected on C1D22 and C4D22 (± 6 days) can be collected anytime that day.
**Pembrolizumab**													
Pharmacokinetics for IV Pembrolizumab	**X**	**X**	**X**	**X**			**X**	**X**	**X**	**X**			Collect **predose** samples 0-4 h before pembrolizumab administration on C1D1, C2D1, C4D1, and C5D1, and at the end of infusion (as soon as possible, but within 5 minutes) on C1D1 and C4D1. Samples collected on C1D22 and C4D22 (± 6 days) can be collected anytime that day.

Note: If the sample day falls on a weekend or holiday and the sample cannot be drawn, an alternate sampling day will not occur.
Abbreviations: C=Cycle; D=Day; IV=intravenous; PK=pharmacokinetics.

2 INTRODUCTION

Pembrolizumab is a potent humanized immunoglobulin G4 (IgG4) monoclonal antibody (mAb) with high specificity of binding to the programmed cell death 1 (PD-1) receptor, thus inhibiting its interaction with programmed cell death ligand 1 (PD-L1) and programmed cell death ligand 2 (PD-L2). Based on preclinical in vitro data, pembrolizumab has high affinity and potent receptor blocking activity for PD-1. Pembrolizumab has an acceptable preclinical safety profile and is in clinical development as an intravenous (IV) immunotherapy for advanced malignancies. KEYTRUDA® (pembrolizumab) is indicated for the treatment of patients across a number of indications. This study will assess the pharmacokinetics (PK), safety and tolerability of pembrolizumab when administered as a subcutaneous (SC) injection and when administered every 6 week (Q6W). Refer to the pembrolizumab Investigator's Brochures (IBs) for further information.

2.1 Study Rationale

Pembrolizumab is currently indicated for the treatment of patients with melanoma, non-small cell lung carcinoma (NSCLC), head and neck squamous cell carcinoma, classical Hodgkin lymphoma, urothelial carcinoma, microsatellite instability-high or mismatch repair deficient solid tumors and gastric cancer. Adult patients are currently to be treated with pembrolizumab at an IV dose of 200 mg every 3 weeks (Q3W). For participants in Cohort A, the present study is designed to estimate the relative bioavailability of a SC versus an IV dose of pembrolizumab, and to characterize the PK profile of pembrolizumab administered SC. During the first 3 treatment cycles, each participant will receive an IV infusion of 200 mg pembrolizumab and 2 SC injections of [redacted] pembrolizumab [redacted] Participants are then to receive an IV infusion of 200 mg pembrolizumab from Cycles 4 to 35. In Cohort B, participants will receive IV infusion of 400 mg pembrolizumab Q6W from Cycles 1 to 18. PK, efficacy, and safety data will be collected from these participants. Enrollment into Cohort A takes priority over Cohort B.

Participants in the study will be limited to those with a diagnosis of advanced melanoma. The cross-over design has been chosen to eliminate effects of interindividual variability inherent in a parallel-group design. The relative bioavailability of pembrolizumab will be estimated in this study using the SC dose [redacted] given at 2 different concentrations/volumes) of pembrolizumab compared with the IV dose of pembrolizumab (200 mg). The results of this study will contribute to an understanding of the PK characteristics of pembrolizumab when administered SC. For Cohort B, results will provide preliminary PK, efficacy, and safety data of pembrolizumab when administered Q6W.

Details regarding specific benefits and risks for participants in this clinical trial may be found in the accompanying pembrolizumab IBs and Informed Consent Form (ICF) document.

2.2 Background

Refer to the IBs/approved labeling for detailed background information on pembrolizumab.

2.2.1 Pharmaceutical and Therapeutic Background

The importance of intact immune surveillance function in controlling outgrowth of neoplastic transformations has been known for decades [Disis, M. L. 2010]. Accumulating evidence shows a correlation between tumor-infiltrating lymphocytes in cancer tissue and favorable prognosis in various malignancies. In particular, the presence of cluster of differentiation (CD)8+ T-cells and the ratio of CD8+ effector T-cells/FoxP3+ regulatory T-cells correlates with improved prognosis and long-term survival in solid malignancies, such as ovarian, colorectal, and pancreatic cancer; hepatocellular carcinoma; malignant melanoma; and renal cell carcinoma. Tumor-infiltrating lymphocytes can be expanded ex vivo and reinfused, inducing durable objective tumor responses in cancers such as melanoma [Dudley, M. E., et al 2005] [Hunder, N. N., et al 2008].

The PD-1 receptor-ligand interaction is a major pathway hijacked by tumors to suppress immune control. The normal function of PD-1, expressed on the cell surface of activated T-cells under healthy conditions, is to down-modulate unwanted or excessive immune responses, including autoimmune reactions. PD-1 (encoded by the gene *Pdcd1*) is an immunoglobulin (Ig) superfamily member related to CD28 and cytotoxic T-lymphocyte-associated protein 4 (CTLA-4) that has been shown to negatively regulate antigen receptor signaling upon engagement of its ligands (PD-L1 and/or PD-L2) [Greenwald, R. J., et al 2005] [Okazaki, T., et al 2001].

The structure of murine PD-1 has been resolved [Zhang, X., et al 2004]. PD-1 and its family members are type I transmembrane glycoproteins containing an Ig-variable–type domain responsible for ligand binding and a cytoplasmic tail responsible for the binding of signaling molecules. The cytoplasmic tail of PD-1 contains 2 tyrosine-based signaling motifs, an immunoreceptor tyrosine-based inhibition motif, and an immunoreceptor tyrosine-based switch motif. Following T-cell stimulation, PD-1 recruits the tyrosine phosphatases, SHP-1 and SHP-2, to the immunoreceptor tyrosine-based switch motif within its cytoplasmic tail, leading to the dephosphorylation of effector molecules such as CD3 zeta, protein kinase C-theta, and zeta-chain-associated protein kinase, which are involved in the CD3 T-cell signaling cascade [Okazaki, T., et al 2001] [Chemnitz, J. M., et al 2004] [Sheppard, K-A, et al 2004] [Riley, J. L. 2009]. The mechanism by which PD-1 down-modulates T-cell responses is similar to, but distinct from, that of CTLA-4, because both molecules regulate an overlapping set of signaling proteins [Parry, R. V., et al 2005] [Francisco, L. M., et al 2010]. As a consequence, the PD-1/PD-L1 pathway is an attractive target for therapeutic intervention in melanoma.

2.2.2 Pre-clinical and Clinical Studies

Pembrolizumab is approved for treatment of patients with melanoma in several countries; in the United States (US), European Union (EU), Australia, and New Zealand it is approved for the treatment of patients with advanced (unresectable or metastatic) melanoma in adults. The Sponsor is studying pembrolizumab for various oncology indications. Pharmacokinetic, efficacy, and safety data from the pembrolizumab clinical development program are presented in the IBs from participants with advanced melanoma, NSCLC, advanced solid

tumors, and hematological cancers. The observed PK profile of pembrolizumab was typical when compared with other IgG mAbs with a half-life of approximately 3 weeks. The overall response rates for pembrolizumab compared favorably to historical response rates for available treatments, particularly in participants who have progressed after multiple prior therapies. Adverse events (AEs) were generally manageable and infrequently required discontinuation of pembrolizumab treatment.

For more details on completed preclinical and clinical studies refer to the IBs.

2.2.3 Ongoing Clinical Studies

The Sponsor is advancing a broad and fast-growing clinical development program for pembrolizumab, both as a monotherapy and in combination with other therapies across more than 30 tumor types and enrolling more than 16,000 participants.

For more details on ongoing clinical studies refer to the IBs.

2.2.4 Information on Other Study-related Therapy

Not applicable.

2.3 Benefit/Risk Assessment

It cannot be guaranteed that participants in clinical studies will directly benefit from treatment during participation, as clinical studies are designed to provide information about the safety and effectiveness of an investigational medicine.

Additional details regarding specific benefits and risks for participants participating in this clinical study may be found in the accompanying IB and ICF documents.

3 HYPOTHESIS, OBJECTIVES, AND ENDPOINTS

Male/female participants of at least 18 years of age with advanced melanoma will be enrolled in this study.

Objectives	Endpoints
Primary	
Cohort A<br>• To characterize the pharmacokinetic (PK) profile, including the absorption phase, of pembrolizumab following subcutaneous (SC) injection CCI [redacted]<br>• To estimate the relative bioavailability of pembrolizumab via SC injection CCI [redacted] versus intravenous (IV) infusion (200 mg: 25 mg/mL)	• Pharmacokinetic parameters of pembrolizumab, including area under the curve (AUC), maximum concentration (C_{max}), bioavailability (F), absorption rate (Ka), time of maximum concentration (T_{max}), clearance (CL), and central volume of distribution (Vc)
Primary	
Cohort B<br>• To evaluate objective response rate (ORR) per RECIST 1.1 as assessed by blinded independent central review (BICR)	• Participants who have a confirmed CR or PR
Secondary	
Cohort A<br>• To evaluate the development of circulating anti-pembrolizumab antibodies (if applicable), following SC pembrolizumab	• Antidrug antibody levels
• To determine the safety and tolerability of SC pembrolizumab during the first 3 treatment cycles based on the proportion of adverse events (AEs)	• Adverse events (AEs)<br>• Study drug discontinuations due to AEs
• To monitor the safety and tolerability of IV pembrolizumab during the first 3 treatment cycles and after Cycle 3 based on the proportion of AEs	• Adverse events (AEs)<br>• Study drug discontinuations due to AEs

Objectives	Endpoints
• To determine the tolerability of SC pembrolizumab at the injection site during the first 3 treatment cycles based on the proportion of injection site signs and symptoms	• Responses to an injection site signs and symptoms questionnaire
Secondary	
Cohort B<br>• To evaluate duration of response (DOR) per RECIST 1.1 as assessed by BICR	• For participants who demonstrate CR or PR, DOR is defined as the time from first documented evidence of CR or PR until disease progression or death due to any cause, whichever occurs first
• To evaluate progression-free survival (PFS) as assessed by BICR according to RECIST v1.1, modified to follow a maximum of 10 target lesions and a maximum of 5 target lesions per organ	• PFS is the time from the first dose of study intervention to the first documented disease progression or death due to any cause, whichever occurs first
• To evaluate overall survival (OS)	• OS is defined as the time from the first day of study treatment to death due to any cause
• To characterize the PK profile of pembrolizumab following IV infusion Q6W	• Pharmacokinetic parameters of pembrolizumab, including AUC, C_{max}, and C_{min}
• To monitor the safety and tolerability of IV pembrolizumab Q6W based on the proportion of AEs	• Adverse events (AEs)<br>• Study drug discontinuations due to AEs
Exploratory	
Cohort B<br>• To evaluate the development of circulating anti-pembrolizumab antibodies (if applicable), following IV pembrolizumab Q6W	• Antidrug antibody levels

4 STUDY DESIGN

4.1 Overall Design

This is a randomized, cross-over, multicenter, open-label, bioavailability, and safety study of pembrolizumab in participants with advanced melanoma to be conducted in conformance with Good Clinical Practices (GCP). This Phase 1 study will be conducted in participants with unresectable or metastatic melanoma.

In Cohort A, the treatment period will continue every 21 days for up to 35 cycles (approximately 2 years). In Cohort B, the treatment period will continue every 42 days for up to 18 cycles (approximately 2 years). Treatment will continue as long as participants are receiving benefit from treatment and have not had disease progression or met any criteria for study withdrawal.

In greater detail, the study consists of:

- A screening period of up to a 28-day duration to ensure that the participant is eligible for the study.

- An intervention period of approximately 104 weeks of treatment with pembrolizumab. In Cohort A, this period will include 2 cycles of pembrolizumab administered via SC injection and 1 cycle of IV infusion over 30 minutes in a 6-sequence cross-over design. Participants will then continue with up to 32 additional cycles of pembrolizumab treatment administered by IV infusion over 30 minutes every 3 weeks. In Cohort B, participants will receive pembrolizumab via IV infusion over 30 minutes Q6W for up to 18 cycles.

- A follow-up period during which participants will be monitored for AEs for 30 days and serious adverse events (SAEs) for 90 days (30 days if the participant initiates new anticancer therapy). Participants with an ongoing AE at the time of treatment discontinuation will be followed until resolution, stabilization, the event is otherwise explained, or the participant is lost to follow-up.

- In Cohort B, participants who discontinue for reasons other than radiographic disease progression will have post-treatment follow-up imaging for disease status until disease progression is documented radiographically per RECIST 1.1 and, when clinically appropriate, confirmed by the site per iRECIST, initiating a non-study cancer treatment, withdrawing consent, becoming lost to follow-up or the end of the study. All participants will be followed by telephone for overall survival in the Survival follow-Up period until death, participant withdrawal of consent, becoming lost to follow-up or the end of the study.

Upon study completion, participants may be enrolled in a pembrolizumab extension study if available.

Specific procedures to be performed during the study, as well as their prescribed times and associated visit windows, are outlined in the SoA in Section 1.3. Details of each procedure are provided in Section 8.

4.2 Scientific Rationale for Study Design

In more than 80 countries including the US, EU, Japan, Australia, and New Zealand, pembrolizumab is indicated for the treatment of patients with unresectable or metastatic melanoma. All participants enrolled into this study will have a diagnosis of advanced melanoma. The cross-over design has been chosen to eliminate effects of interindividual variability inherent in a parallel-group design. The results of this study will contribute to an understanding of the PK characteristics of pembrolizumab administered SC, and when administered in a Q6W dosing regimen.

4.2.1 Rationale for Endpoints

4.2.1.1 Safety Endpoints

Cohort A

A secondary objective of this study is to characterize the safety and tolerability of pembrolizumab when administered SC as monotherapy. The primary safety analysis will be based on participants who experience toxicities. Safety will be assessed by quantifying the toxicities and grades of toxicities experienced by participants who have received pembrolizumab via SC injection and IV infusion. An additional secondary objective is to determine the tolerability of SC pembrolizumab at the injection site during the first 3 treatment cycles based on the proportion of injection site signs and symptoms.

Cohort B

In Cohort B, a secondary objective of this study is to characterize the safety and tolerability of 400 mg pembrolizumab when administered IV Q6W.

Safety parameters commonly used for evaluating investigational systemic anticancer treatments are included as safety endpoints including, but not limited to, the incidence of, causality, and outcome of adverse events (AEs)/serious adverse events (SAEs); and changes in vital signs and laboratory values. AEs will be assessed as defined by CTCAE, Version [4.0].

4.2.1.2 Pharmacokinetic Endpoints

Cohort A

A primary objective of this trial is to characterize the PK profile and relative bioavailability of pembrolizumab following administration as a SC injection versus an IV infusion. The serum concentrations of these agents will serve as the primary readout for the PK, and these data will be used to derive PK parameters. Furthermore, the results of these analyses will be

used in conjunction with the safety and exploratory endpoint data to help assess future dosing strategies for administering pembrolizumab SC.

To evaluate pembrolizumab immunogenicity and pembrolizumab exposure after SC administration, sample collections for analysis of antidrug antibodies (ADA) and PK are currently planned as shown in the SoA (Section 1.3). Blood samples will be obtained to measure PK of serum pembrolizumab. The pembrolizumab serum concentrations at planned visits and times will be summarized.

Pharmacokinetic data will also be analyzed using nonlinear mixed effects modeling. Based on PK data obtained in this study as well as PK data obtained from previous studies, an integrated population PK analysis will be performed to characterize PK parameters (absorption rate [Ka], bioavailability [F], clearance [CL], central volume of distribution [Vc]) of pembrolizumab following SC and IV administration.

Pharmacokinetic endpoints in this study include:

- Pharmacokinetic parameters of pembrolizumab , including area under the curve (AUC), maximum concentration (C_{max}), bioavailability (F), absorption rate (Ka), time of maximum concentration (T_{max}), clearance (CL), and central volume of distribution (Vc).

Cohort B

In Cohort B, an objective of this trial is to characterize the PK profile of pembrolizumab following administration as an IV infusion Q6W. PK data will be analyzed after all participants complete Cycle 5. PK parameters will include AUC, C_{max}, and C_{min}.

4.2.1.3 Antidrug Antibodies (ADA)

Formation of ADAs can potentially confound drug exposures at therapeutic doses and prime for subsequent infusion-related toxicity. Antidrug antibody response to pembrolizumab at the beginning of each of the first 4 cycles (Cohort A: Q3W pembrolizumab dosing), as well as Cycles 1, 2, 4, and 5 (Cohort B: Q6W pembrolizumab dosing) will be determined. Any impact of presence of ADAs on exposure of pembrolizumab will be explored.

4.2.1.4 Efficacy Endpoints

Cohort B

This study will use ORR based on RECIST 1.1 criteria as assessed by blinded independent central review (BICR) as the primary endpoint. Objective response rate is an acceptable measure of clinical benefit for a late stage study that demonstrates superiority of a new antineoplastic therapy, especially if the magnitude of the effect is large and the therapy has an acceptable risk/benefit profile. The use of BICR and RECIST 1.1 to assess ORR is typically considered acceptable by regulatory authorities. Images will be submitted to an

imaging CRO (iCRO) and read by independent central review blinded to treatment assignment to minimize bias in the response assessments.

Overall survival (OS) is a secondary endpoint for Cohort B and has been recognized as the gold standard for the demonstration of superiority of a new antineoplastic therapy in randomized clinical studies.

RECIST 1.1 will be used by the BICR when assessing images for efficacy measures and by the local site when determining eligibility (Section 8.2.1.4). Although traditional RECIST 1.1 references a maximum of 5 target lesions in total and 2 per organ, this protocol has implemented a modification to RECIST 1.1 to allow a maximum of 10 target lesions in total and 5 per organ. RECIST 1.1 will be adapted to account for the unique tumor response characteristics seen following treatment with pembrolizumab (Section 8.2.1.5). Immunotherapeutic agents such as pembrolizumab may produce antitumor effects by potentiating endogenous cancer-specific immune responses. The response patterns seen with such an approach may extend beyond the typical time course of responses seen with cytotoxic agents, and patients treated with pembrolizumab may manifest a clinical response after an initial increase in tumor burden or even the appearance of new lesions. Thus, standard RECIST 1.1 may not provide an accurate response assessment of immunotherapeutic agents such as pembrolizumab. Based on an analysis of participants with melanoma enrolled in KEYNOTE-001 (KN001), 7% of evaluable participants experienced delayed or early tumor pseudo-progression. Of note, participants who had progressive disease (PD) by RECIST 1.1 but not by the immune-related response criteria [Wolchok, J. D., et al 2009] had longer overall survival than participants with PD by both criteria [Hodi, F. S., et al 2014]. Additionally, the data suggest that RECIST 1.1 may underestimate the benefit of pembrolizumab in approximately 15% of participants. These findings support the need to apply a modification to RECIST 1.1 that takes into account the unique patterns of atypical responses in immunotherapy and enables treatment beyond initial radiographic progression, if the participant is clinically stable.

Modified RECIST 1.1 for immune-based therapeutics (iRECIST) assessment has been developed and published by the RECIST Working Group, with input from leading experts from industry and academia, along with participation from the US Food and Drug Administration and the European Medicines Agency [Seymour, L., et al 2017]. The unidimensional measurement of target lesions, qualitative assessment of nontarget lesions, and response categories are identical to RECIST 1.1, until progression is seen by RECIST 1.1. However, if a participant is clinically stable, additional imaging may be performed to confirm radiographic progression. iRECIST will be used by investigators to assess tumor response and progression and make treatment decisions as well as for exploratory efficacy analyses where specified.

4.3 Justification for Dose

The primary purpose of Cohort A in this study is to establish the SC dose and volume for further clinical development. Two SC solutions have been manufactured [redacted] and will be used in Cycles 1 to 3 of this study, corresponding to a dose of [redacted] that assumes 70% bioavailability for the SC formulation relative to IV (dose = 200 mg infusion given over 30 minutes in Cycles 1 to 3) [Zhao, L., et al 2013].

The planned dose of pembrolizumab for Cycle 4 onwards in this study is 200 mg IV infusion Q3W. Based on the totality of data generated in the Keytruda development program, 200 mg Q3W is the appropriate dose of pembrolizumab for adults across all indications and regardless of tumor type. As outlined below, this dose is justified by:

- Clinical data from 8 randomized studies demonstrating flat dose- and exposure-efficacy relationships from 2 mg/kg Q3W to 10 mg/kg every 2 weeks (Q2W),
- Clinical data showing meaningful improvement in benefit-risk including overall survival at 200 mg Q3W across multiple indications, and
- Pharmacology data showing full target saturation in both systemic circulation (inferred from pharmacokinetic [PK] data) and tumor (inferred from physiologically-based PK [PBPK] analysis) at 200 mg Q3W

Among the 8 randomized dose-comparison studies, a total of 2262 participants were enrolled with melanoma and NSCLC, covering different disease settings (treatment-naïve, previously treated, PD-L1 enriched, and all-comers) and different treatment settings (monotherapy and in combination with chemotherapy). Five studies compared 2 mg/kg Q3W versus 10 mg/kg Q3W (KN001 Cohort B2, KN001 Cohort D, KN002, KN010, and KN021), and 3 studies compared 10 mg/kg Q3W versus 10 mg/kg Q2W (KN001 Cohort B3, KN001 Cohort F2 and KN006). All of these studies demonstrated flat dose- and exposure-response relationships across the doses studied representing an approximate 5- to 7.5-fold difference in exposure. The 2 mg/kg (or 200 mg fixed-dose) Q3W provided similar responses to the highest doses studied. Subsequently, flat dose-exposure-response relationships were also observed in other tumor types including head and neck cancer, bladder cancer, gastric cancer and classical Hodgkin lymphoma, confirming 200 mg Q3W as the appropriate dose independent of the tumor type. These findings are consistent with the mechanism of action of pembrolizumab, which acts by interaction with immune cells, and not via direct binding to cancer cells.

Additionally, pharmacology data clearly show target saturation at 200 mg Q3W. First, PK data in KN001 evaluating target-mediated drug disposition (TMDD) conclusively demonstrated saturation of PD-1 in systemic circulation at doses much lower than 200 mg Q3W. Second, a PBPK analysis was conducted to predict tumor PD-1 saturation over a wide range of tumor penetration and PD-1 expression. This evaluation concluded that pembrolizumab at 200 mg Q3W achieves full PD-1 saturation in both blood and tumor.

Finally, population PK analysis of pembrolizumab, which characterized the influence of body weight and other participant covariates on exposure, has shown that the fixed-dosing provides similar control of PK variability as weight based dosing, with considerable overlap in the distribution of exposures from the 200 mg Q3W fixed dose and 2 mg/kg Q3W dose. Supported by these PK characteristics, and given that fixed-dose has advantages of reduced dosing complexity and reduced potential of dosing errors, the 200 mg Q3W fixed-dose was selected for evaluation across all pembrolizumab protocols.

In Cohort B, participants will receive 400 mg IV pembrolizumab Q6W. A 400 mg Q6W dosing regimen of pembrolizumab is expected to have a similar benefit-risk profile as 200 mg Q3W, in all treatment settings in which 200 mg Q3W pembrolizumab is currently approved. Specifically, the dosing regimen of 400 mg Q6W for pembrolizumab is considered adequate based on modeling and simulation (M&S) analyses, given the following rationale:

- PK simulations demonstrating that in terms of pembrolizumab exposures –
  - Average concentration over the dosing interval (C_{avg}) (or area under the curve [AUC]) at 400 mg Q6W is similar to that at the approved 200 mg Q3W dose, thus bridging efficacy between dosing regimens.
  - Trough concentrations (C_{min}) at 400 mg Q6W are generally within the range of those achieved with 2 mg/kg or 200 mg Q3W in the majority (>99%) of patients.
  - Peak concentrations (C_{max}) at 400 mg Q6W are well below the C_{max} for the highest clinically tested dose of 10 mg/kg Q2W, supporting that the safety profile for 400 mg Q6W should be comparable to the established safety profile of pembrolizumab.
- Exposure-response (E-R) for pembrolizumab has been demonstrated to be flat across indications, and OS predictions in melanoma and NSCLC demonstrate that efficacy at 400 mg Q6W is expected to be similar to that at 200 mg or 2 mg/kg Q3W, given the similar exposures; thus 400 mg Q6W is expected to be efficacious across indications.

4.4 Beginning and End of Study Definition

The overall study begins when the first participant signs the ICF. The overall study ends when the last participant completes the last study-related telephone-call or visit, withdraws from the study, or is lost to follow-up (ie, the participant is unable to be contacted by the investigator).

Upon study completion, participants may be enrolled in a pembrolizumab extension study if available.

4.4.1 Clinical Criteria for Early Study Termination

Early study termination will be the result of the criteria specified below:

- Incidence or severity of adverse drug reactions in this or other studies suggest a potential health hazard to participants
- Plans to modify or discontinue the development of the study drug
- Poor adherence to protocol and regulatory requirements
- Quality or quantity of data recording is inaccurate or incomplete
- Ample notification will be provided in the event of Sponsor decision to no longer supply pembrolizumab.

5 STUDY POPULATION

Male/female participants with advanced melanoma of at least 18 years of age on the day of signing informed consent will be enrolled in this study.

Prospective approval of protocol deviations to recruitment and enrollment criteria, also known as protocol waivers or exemptions, is not permitted.

5.1 Inclusion Criteria

Participants are eligible to be included in the study only if all of the following criteria apply:

Type of Participant and Disease Characteristics

1. Have histologically or cytologically confirmed diagnosis of advanced melanoma
2. Have unresectable Stage III or Stage IV melanoma, as per American Joint Committee on Cancer (AJCC) staging system not amenable to local therapy.
3. Have been untreated for advanced or metastatic disease except as follows:
    a. BRAF V600 mutant melanoma may have received standard of care targeted therapy (eg, BRAF/MEK inhibitor, alone or in combination) and be eligible for this study

       Note: Targeted therapy is not required for eligibility.

b. Prior adjuvant or neoadjuvant melanoma therapy is permitted if it was completed at least 4 weeks before randomization and all related AEs have either returned to baseline or stabilized (resolution of toxic effect(s) of the most recent prior therapy to Grade 1 or less [except alopecia]). If subject received major surgery or radiation therapy of >30 Gy, they must have recovered from the toxicity and/or complications from the intervention.

Demographics

Female Participants

4. A female participant is eligible to participate if she is not pregnant (see Appendix 5), not breastfeeding, and at least 1 of the following conditions applies: A WOCBP who agrees to follow the contraceptive guidance in Appendix 5 during the treatment period and for at least 120 days.

   Note: Abstinence is acceptable if this is the usual lifestyle and preferred contraception for the subject.

Informed Consent

5. The participant (or legally acceptable representative if applicable) provides written informed consent for the study.

6. Have measurable disease per RECIST 1.1 as assessed by BICR. Lesions situated in a previously irradiated area are considered measurable if progression has been demonstrated in such lesions.

Additional Categories

7. Have an Eastern Cooperative Oncology Group (ECOG) performance status 0 to 1.

8. Have adequate organ function as defined in the following table (Table 3). Specimens must be collected within 72 hours prior to the start of study intervention.

Table 3 Adequate Organ Function Laboratory Values

**System**	**Laboratory Value**
Hematological	
Absolute neutrophil count (ANC)	≥1500/µL
Platelets	≥100 000/µL
Hemoglobin	≥9.0 g/dL or ≥5.6 mmol/L[1]
Renal	
Creatinine OR<br>Measured or calculated[2] creatinine clearance (GFR can also be used in place of creatinine or CrCl)	≤1.5 × ULN OR<br>≥30 mL/min for participant with creatinine levels >1.5 × institutional ULN
Hepatic	
Total bilirubin	≤1.5 ×ULN OR direct bilirubin ≤ULN for participants with total bilirubin levels >1.5 × ULN
AST (SGOT) and ALT (SGPT)	≤2.5 × ULN (≤5 × ULN for participants with liver metastases)
Coagulation	
International normalized ratio (INR) OR prothrombin time (PT)<br>Activated partial thromboplastin time (aPTT)	≤1.5 × ULN unless participant is receiving anticoagulant therapy as long as PT or PTT is within therapeutic range of intended use of anticoagulants

[1]Criteria must be met without erythropoietin dependency and without packed red blood cell (pRBC) transfusion within last 2 weeks.

[2]Creatinine clearance (CrCl) should be calculated per institutional standard.

Note: This table includes eligibility-defining laboratory value requirements for treatment; laboratory value requirements should be adapted according to local regulations and guidelines for the administration of specific chemotherapies.

ALT (SGPT)=alanine aminotransferase (serum glutamic pyruvic transaminase); AST (SGOT)=aspartate aminotransferase (serum glutamic oxaloacetic transaminase); GFR=glomerular filtration rate; ULN=upper limit of normal.

5.2 Exclusion Criteria

Participants are excluded from the study if any of the following criteria apply:

Medical Conditions

1. A WOCBP who has a positive urine pregnancy test within 72 hours prior to randomization or treatment allocation (see Appendix 5). If the urine test is positive or cannot be confirmed as negative, a serum pregnancy test will be required.

   Note: In the event that 72 hours have elapsed between the screening pregnancy test and the first dose of study treatment, another pregnancy test (urine or serum) must be performed and must be negative in order for subject to start receiving study medication.

Prior/Concomitant Therapy

2. Has received prior systemic treatment for unresectable or metastatic melanoma (except as noted in inclusion criteria #3).

3. Has received prior therapy with an anti-PD-1, anti-PD-L1, or anti-PD-L2 or with an agent directed to another stimulatory or co-inhibitory T-cell receptor (eg, OX-40 and CD137) or any other antibody or drug specifically targeting checkpoint pathways other than anti-CTLA-4 which is permitted in the adjuvant setting.

4. Has received prior radiotherapy within 2 weeks of start of study treatment. Participants must have recovered from all radiation-related toxicities, not require corticosteroids, and not have had radiation pneumonitis. A 1-week washout is permitted for palliative radiation (≤2 weeks of radiotherapy) to non-central nervous system (CNS) disease.

5. Has received a live vaccine within 30 days prior to the first dose of study drug. Examples of live vaccines include, but are not limited to, the following: measles, mumps, rubella, varicella/zoster (chicken pox), yellow fever, rabies, Bacillus Calmette-Guérin (BCG), and typhoid vaccine. Seasonal influenza vaccines for injection are generally killed virus vaccines and are allowed; however, intranasal influenza vaccines (eg, FluMist®) are live attenuated vaccines and are not allowed.

Prior/Concurrent Clinical Study Experience

6. Is currently participating in or has participated in a study of an investigational agent or has used an investigational device within 4 weeks prior to the first dose of study intervention.

   Note: Participants who have entered the follow-up phase of an investigational study may participate as long as it has been 4 weeks after the last dose of the previous investigational agent.

Diagnostic Assessments

7. Has a diagnosis of immunodeficiency or is receiving chronic systemic steroid therapy (in dosing exceeding 10 mg daily of prednisone equivalent) or any other form of immunosuppressive therapy within 7 days prior the first dose of study drug.

8. Has a known additional malignancy that is progressing or has required active treatment within the past 2 years

   Note: Participants with basal cell carcinoma of the skin, squamous cell carcinoma of the skin, or carcinoma in situ (eg, breast carcinoma, cervical cancer in situ) that have undergone potentially curative therapy are not excluded.

9. Has known active CNS metastases and/or carcinomatous meningitis. Participants with previously treated brain metastases may participate provided they are radiologically stable, (ie, without evidence of progression) for at least 4 weeks by repeat imaging (note that the repeat imaging should be performed during study screening), clinically stable and without requirement of steroid treatment for at least 14 days prior to first dose of study intervention.

10. Has severe hypersensitivity (≥Grade 3) to pembrolizumab and/or any of its excipients.

11. Has ocular melanoma.

12. Has an active autoimmune disease that has required systemic treatment in past 2 years (ie, with use of disease modifying agents, corticosteroids or immunosuppressive drugs). Replacement therapy (eg, thyroxine, insulin, or physiologic corticosteroid replacement therapy for adrenal or pituitary insufficiency) is not considered a form of systemic treatment and is allowed.

13. Has a history of (non-infectious) pneumonitis that required steroids or has current pneumonitis.

14. Has an active infection requiring systemic therapy.

15. Has a known history of human immunodeficiency virus (HIV) infection. No HIV testing is required unless mandated by local health authority.

16. Has a known history of Hepatitis B (defined as Hepatitis B surface antigen [HBsAg] reactive) or known active Hepatitis C virus (defined as HCV RNA [qualitative] is detected) infection.

Note: No testing for Hepatitis B and Hepatitis C is required unless mandated by local health authority.

17. Has a history or current evidence of any condition, therapy, or laboratory abnormality that might confound the results of the study, interfere with the participant's participation for the full duration of the study, or is not in the best interest of the participant to participate, in the opinion of the treating investigator.

18. Has a known psychiatric or substance abuse disorder that would interfere with cooperating with the requirements of the study.

Other Exclusions

19. Is pregnant or breastfeeding or expecting to conceive or father children within the projected duration of the study, starting with the screening visit through 120 days after the last dose of study intervention.

20. Has had an allogenic tissue/solid organ transplant.

5.3 Lifestyle Considerations

5.3.1 Meals and Dietary Restrictions

Participants should maintain a normal diet unless modifications are required to manage an AE such as diarrhea, nausea, or vomiting.

5.3.2 Caffeine, Alcohol, and Tobacco Restrictions

There are no restrictions on caffeine, alcohol, or tobacco.

5.3.3 Activity Restrictions

There are no restrictions.

5.3.4 Contraception

Pembrolizumab may have adverse effects on a fetus in utero. Refer to Appendix 5 for approved methods of contraception.

Participants should be informed that taking the study medication may involve unknown risks to the fetus (unborn baby) if pregnancy were to occur during the study. In order to participate in the study, participants of childbearing potential must adhere to the contraception requirement (Appendix 5) from the day of study medication initiation (or 14 days prior to the initiation of study medication for oral contraception) throughout the study period up to 120 days after the last dose of study medication. If there is any question that a participant of childbearing potential will not reliably comply with the requirements for contraception, that participant should not be entered into the study.

5.3.5 Pregnancy

If a participant inadvertently becomes pregnant while on treatment with pembrolizumab, the participant will be immediately discontinued from study intervention. The site will contact the participant at least monthly and document the participant's status until the pregnancy has been completed or terminated. The outcome of the pregnancy will be reported to the Sponsor without delay and within 24 hours if the outcome is a serious adverse experience (eg, death, abortion, congenital anomaly, or other disabling or life-threatening complication to the mother or newborn). The study Investigator will make every effort to obtain permission to follow the outcome of the pregnancy and report the condition of the fetus or newborn to the Sponsor. If a male participant impregnates his female partner, the study personnel at the site must be informed immediately and the pregnancy must be reported to the Sponsor and followed as described in Section 8.4.

5.3.6 Use in Nursing Women

It is unknown whether pembrolizumab is excreted in human milk. Since many drugs are excreted in human milk, and because of the potential for serious adverse reactions in the nursing infant, participants who are breastfeeding are not eligible for enrollment.

5.4 Screen Failures

Screen failures are defined as participants who consent to participate in the clinical study, but are not subsequently randomized in the study. A minimal set of screen failure information is required to ensure transparent reporting of screen failure participants to meet the Consolidated Standards of Reporting Trials (CONSORT) publishing requirements and to respond to queries from regulatory authorities. Minimal information includes demography, screen failure details, eligibility criteria, and any AEs or SAEs meeting reporting requirements as outlined in the data entry guidelines.

5.5 Participant Replacement Strategy

Cohort A

If a participant discontinues from study intervention OR withdraws from the study prior to completing Cycle 3, a replacement participant may be enrolled if deemed appropriate by the investigator and Sponsor. The replacement participant will be assigned a unique treatment/randomization number. The replacement participant will be allocated to the same treatment arm as the participant who is being replaced. The study site should contact the Sponsor for the replacement participant's treatment/randomization number.

Cohort B

In Cohort B, if a participant discontinues from study intervention OR withdraws from the study, they will not be replaced.

6 STUDY INTERVENTION

Study intervention is defined as any investigational intervention(s), marketed product(s), placebo, or medical device(s) intended to be administered to a study participant according to the study protocol.

Clinical supplies (study intervention[s] provided by the Sponsor) will be packaged to support enrollment and replacement participants as required. When a replacement participant is required, the Sponsor or designee needs to be contacted prior to dosing the replacement supplies. Clinical supplies will be affixed with a clinical label in accordance with regulatory requirements.

6.1 Study Intervention(s) Administered

The study intervention(s) to be used in this study are outlined in Table 4.

Table 4 Study Interventions

Arm Name	Arm Type	Interven-tion Name	Type	Dose Formulation	Unit Dose Strength(s)	Dosage Level(s)	Route of Administration	Regimen/ Treatment Period/ Vaccination Regimen	Use	IMP/ NIMP	Sourcing
All Arms in Cohort A	Experimental	MK-3475	Biological	Sterile Solution for IV Infusion	25 mg/mL	200 mg	IV Infusion	Every 3 weeks	Experi mental	IMP	Provided centrally by Sponsor
All Arms in Cohort A	Experimental	MK-3475	Biological	Sterile Solution for SC Injection	CCI [redacted]	[redacted]	SC Injection	Every 3 weeks	Experi mental	IMP	Provided centrally by Sponsor
All Arms in Cohort A	Experimental	MK-3475	Biological	Sterile Solution for SC Injection	[redacted]	[redacted]	SC Injection	Every 3 weeks	Experi mental	IMP	Provided centrally by Sponsor
Cohort B	Experimental	MK-3475	Biological	Sterile Solution for IV Infusion	25 mg/mL	400 mg	IV Infusion	Every 6 weeks	Experi mental	IMP	Provided centrally by Sponsor
Definition Investigational Medicinal Product (IMP) and Non-Investigational Medicinal Product (NIMP) are based on guidance issued by the European Commission. Regional and/or Country differences of the definition of IMP/NIMP may exist. In these circumstances, local legislation is followed.<br><br>Abbreviations: IV=intravenous; SC=subcutaneous.											

All study interventions will be administered on an outpatient basis.

All products indicated in Table 4 will be provided centrally by the Sponsor or locally by the study site, subsidiary or designee, depending on local country operational or regulatory requirements.

For any commercially available product that is provided by the study site, subsidiary, or designee, every attempt will be made to source these supplies from a single lot/batch number. The study site is responsible for recording the lot number, manufacturer, and expiry date for any locally purchased product as per local guidelines unless otherwise instructed by the Sponsor.

Refer to Section 8.1.8 for details regarding administration of the study intervention.

6.1.1 Medical Devices

Not applicable.

6.2 Preparation/Handling/Storage/Accountability

6.2.1 Dose Preparation

Details on preparation and administration of pembrolizumab are provided in the Pharmacy Manual.

There are no specific calculations or evaluations required to be performed in order to administer the proper dose to each participant. The rationale for selection of doses to be used in this study is provided in Section 4.3.

6.2.2 Handling, Storage, and Accountability

The investigator or designee must confirm appropriate temperature conditions have been maintained during transit for all study intervention received, and any discrepancies are reported and resolved before use of the study intervention.

Only participants enrolled in the study may receive study intervention, and only authorized site staff may supply or administer study intervention. All study interventions must be stored in a secure, environmentally controlled, and monitored (manual or automated) area in accordance with the labeled storage conditions with access limited to the investigator and authorized site staff.

The investigator, institution, or the head of the medical institution (where applicable) is responsible for study intervention accountability, reconciliation, and record maintenance (ie, receipt, reconciliation, and final disposition records).

For all study sites, the local country Sponsor personnel or designee will provide appropriate documentation that must be completed for drug accountability and return, or local discard

and destruction if appropriate. Where local discard and destruction is appropriate, the investigator is responsible for ensuring that a local discard/destruction procedure is documented.

The study site is responsible for recording the lot number, manufacturer, and expiry date for any locally purchased product (if applicable) as per local guidelines unless otherwise instructed by the Sponsor.

The investigator shall take responsibility for and shall take all steps to maintain appropriate records and ensure appropriate supply, storage, handling, distribution, and usage of study interventions in accordance with the protocol and any applicable laws and regulations.

6.3 Measures to Minimize Bias: Randomization and Blinding

6.3.1 Intervention Assignment

Enrollment into Cohort A takes priority over Cohort B.

Cohort A

No Interactive Voice Recognition System (IVRS) will be used in Cohort A. Participants will be assigned randomly according to a computer-generated allocation schedule, and will be assigned uniformly to 1 of 6 treatment arms (Table 5).

A sample allocation schedule for the treatment plan of this study in Cohort A is provided in Table 5.

Table 5 Sample Allocation Schedule (Cohort A)

Treatment Arm	Cycle 1	Cycle 2	Cycle 3	Cycles 4-35
1 (N=5)	B	C	A	D
2 (N=5)	B	A	C	D
3 (N=5)	A	B	C	D
4 (N=5)	A	C	B	D
5 (N=5)	C	A	B	D
6 (N=5)	C	B	A	D

Note: A [redacted] SC; B [redacted] SC; C=200 mg IV; D=200 mg IV.
Abbreviations: IV=intravenous; SC=subcutaneous.

Cohort B

In Cohort B, all participants will receive 400 mg IV pembrolizumab Q6W. Study treatment will be allocated via IVRS.

6.3.2 Stratification

No stratification based on age, sex, or other characteristics will be used in this study.

6.3.3 Blinding

This is an open-label study; therefore, the Sponsor, investigator, and participant will know the intervention administered.

6.4 Study Intervention Compliance

Administration of study medication will be witnessed by the investigator and/or study staff. The total volume of study medication infused or injected will be compared with the total volume prepared to determine compliance with each dose administered.

Interruptions from the protocol-specified treatment plan for more than 12 weeks between pembrolizumab doses for nondrug-related or administrative reasons require consultation between the investigator and the Sponsor and written documentation of the collaborative decision on participant management.

6.5 Concomitant Therapy

Medications or vaccinations specifically prohibited in the exclusion criteria are not allowed. If there is a clinical indication for any medication or vaccination specifically prohibited, discontinuation from study therapy or vaccination may be required. The investigator should discuss any questions regarding this with the Sponsor's Clinical Director. The final decision on any supportive therapy or vaccination rests with the investigator and/or the participant's primary physician. However, the decision to continue the participant on study intervention requires the mutual agreement of the investigator, the Sponsor, and the participant.

Participants are prohibited from receiving the following therapies during the screening and treatment periods of this study:

- Immunotherapy not specified in this protocol
- Antineoplastic systemic chemotherapy or biological therapy not specified in this protocol
- Investigational agents not specified in this protocol. Radiation therapy (radiotherapy for symptom management is allowed)

- Live vaccines within 30 days prior to the first dose of study intervention and while participating in the study. Examples of live vaccines include, but are not limited to, the following: measles, mumps, rubella, varicella/zoster, yellow fever, rabies, BCG, and typhoid vaccine. Seasonal influenza vaccines for injection are generally killed virus vaccines and are allowed; however, intranasal influenza vaccines (eg, FluMist®) are live attenuated vaccines and are not allowed.

- Glucocorticoids other than to modulate symptoms from an immune-mediated AE. Chronic systemic replacement doses of steroids and non-systemic steroids including inhaled steroids, topical steroids, intra-nasal steroids, intra-articular, and ophthalmic steroids are allowed that is suspected to have an immunologic etiology. The use of physiologic doses of corticosteroids may be approved after consultation with the Sponsor.

There are no prohibited therapies after Post-Treatment Safety follow-up visit.

Participants who, in the assessment of the Investigator, require the use of any of the aforementioned treatments for clinical management should be removed from the treatment period.

All treatments that the investigator considers necessary for a participant's welfare may be administered at the discretion of the investigator in keeping with the community standards of medical care. All concomitant medication will be recorded on the eCRF including all prescription, over-the-counter (OTC) products, herbal supplements, and IV medications and fluids.

All concomitant medications received within 28 days prior to the first dose of study intervention and up to 30 days after the last dose of study intervention should be recorded. Concomitant medications administered 30 days after the last dose of study intervention should be recorded for SAEs and events of clinical interest (ECIs) as defined in Section 8.4.7.

6.5.1 Rescue Medications and Supportive Care

Participants should receive appropriate supportive care measures as deemed necessary by the treating investigator. Suggested supportive care measures for the management of AEs with potential immunologic etiology are outlined along with the dose modification guidelines in Section 6.6, Table 6. Where appropriate, these guidelines include the use of oral or IV treatment with corticosteroids, as well as additional anti-inflammatory agents if symptoms do not improve with administration of corticosteroids. Note that several courses of steroid tapering may be necessary as symptoms may worsen when the steroid dose is decreased. For each disorder, attempts should be made to rule out other causes such as metastatic disease or bacterial or viral infection, which might require additional supportive care. The treatment guidelines are intended to be applied when the investigator determines the events to be related to pembrolizumab.

Note: If after the evaluation of the event, it is determined not to be related to pembrolizumab, the investigator does not need to follow the treatment guidance. Refer to Table 6 in Section 6.6.1 for guidelines regarding dose modification and supportive care.

It may be necessary to perform conditional procedures such as bronchoscopy, endoscopy, or skin photography as part of evaluation of the event.

6.6 Dose Modification (Escalation/Titration/Other)

6.6.1 Immune-Related Events and Dose Modification (Withhold, Treat, Discontinue)

Dose Modification and Toxicity Management for Immune-related AEs Associated with Pembrolizumab

AEs associated with pembrolizumab exposure may represent an immune-related response. These irAEs may occur shortly after the first dose or several months after the last dose of pembrolizumab treatment and may affect more than one body system simultaneously. Therefore, early recognition and initiation of treatment is critical to reduce complications. Based on existing clinical study data, most irAEs were reversible and could be managed with interruptions of pembrolizumab, administration of corticosteroids and/or other supportive care. For suspected irAEs, ensure adequate evaluation to confirm etiology or exclude other causes. Additional procedures or tests such as bronchoscopy, endoscopy, skin biopsy may be included as part of the evaluation. Based on the severity of irAEs, withhold or permanently discontinue pembrolizumab and administer corticosteroids.

Dose Modification and Toxicity Management Guidelines for irAEs associated with pembrolizumab monotherapy, coformulations, or IO combinations are provided in Table 6.

Table 6 Dose Modification and Toxicity Management Guidelines for Immune-related Adverse Events Associated with Pembrolizumab Monotherapy, Coformulations or IO Combinations

General instructions:

1. Severe and life-threatening irAEs should be treated with IV corticosteroids followed by oral steroids. Other immunosuppressive treatment should begin if the irAEs are not controlled by corticosteroids.
2. Pembrolizumab monotherapy, coformulations or IO combinations must be permanently discontinued if the irAE does not resolve or the corticosteroid dose is not ≤10 mg/day within 12 weeks of the last treatment.
3. The corticosteroid taper should begin when the irAE is ≤ Grade 1 and continue at least 4 weeks.
4. If pembrolizumab monotherapy, coformulations or IO combinations have been withheld, treatment may resume after the irAE decreased to ≤ Grade 1 after corticosteroid taper.

irAEs	Toxicity Grade (CTCAEv4.0)	Action With Pembrolizumab Monotherapy, Coformulations or IO Combinations	Corticosteroid and/or Other Therapies	Monitoring and Follow-up
Pneumonitis	Grade 2	Withhold	• Administer corticosteroids (initial dose of 1-2 mg/kg prednisone or equivalent) followed by taper	• Monitor participants for signs and symptoms of pneumonitis<br>• Evaluate participants with suspected pneumonitis with radiographic imaging and initiate corticosteroid treatment<br>• Add prophylactic antibiotics for opportunistic infections
	Recurrent Grade 2 or Grade 3 or 4	Permanently discontinue		

irAEs	Toxicity Grade (CTCAEv4.0)	Action With Pembrolizumab Monotherapy, Coformulations or IO Combinations	Corticosteroid and/or Other Therapies	Monitoring and Follow-up
Diarrhea / Colitis	Grade 2 or 3	Withhold	• Administer corticosteroids (initial dose of 1-2 mg/kg prednisone or equivalent) followed by taper	• Monitor participants for signs and symptoms of enterocolitis (ie, diarrhea, abdominal pain, blood or mucus in stool with or without fever) and of bowel perforation (ie, peritoneal signs and ileus)<br>• Participants with ≥Grade 2 diarrhea suspecting colitis should consider GI consultation and performing endoscopy to rule out colitis<br>• Participants with diarrhea/colitis should be advised to drink liberal quantities of clear fluids. If sufficient oral fluid intake is not feasible, fluid and electrolytes should be substituted via IV infusion.
	Recurrent Grade 3 or Grade 4	Permanently discontinue		
AST / ALT Elevation or Increased Bilirubin	Grade 2	Withhold	• Administer corticosteroids (initial dose of 0.5-1 mg/kg prednisone or equivalent) followed by taper	• Monitor with liver function tests (consider weekly or more frequently until liver enzyme value returned to baseline or is stable)
	Grade 3 or 4	Permanently discontinue	• Administer corticosteroids (initial dose of 1-2 mg/kg prednisone or equivalent) followed by taper	

irAEs	Toxicity Grade (CTCAEv4.0)	Action With Pembrolizumab Monotherapy, Coformulations or IO Combinations	Corticosteroid and/or Other Therapies	Monitoring and Follow-up
T1DM or Hyperglycemia	New onset T1DM or<br>Grade 3 or 4 hyperglycemia associated with evidence of β-cell failure	Withhold [a]	• Initiate insulin replacement therapy for participants with T1DM<br>• Administer anti-hyperglycemic in participants with hyperglycemia	• Monitor participants for hyperglycemia or other signs and symptoms of diabetes
Hypophysitis	Grade 2	Withhold	• Administer corticosteroids and initiate hormonal replacements as clinically indicated	• Monitor for signs and symptoms of hypophysitis (including hypopituitarism and adrenal insufficiency)
	Grade 3 or 4	Withhold or permanently discontinue [a]		
Hyperthyroidism	Grade 2	Continue	• Treat with non-selective beta-blockers (eg, propranolol) or thionamides as appropriate	• Monitor for signs and symptoms of thyroid disorders
	Grade 3 or 4	Withhold or<br>Permanently discontinue [a]		

irAEs	Toxicity Grade (CTCAEv4.0)	Action With Pembrolizumab Monotherapy, Coformulations or IO Combinations	Corticosteroid and/or Other Therapies	Monitoring and Follow-up
Hypothyroidism	Grade 2-4	Continue	• Initiate thyroid replacement hormones (eg, levothyroxine or liothyronine) per standard of care	• Monitor for signs and symptoms of thyroid disorders
Nephritis and renal dysfunction	Grade 2	Withhold	• Administer corticosteroids (prednisone 1-2 mg/kg or equivalent) followed by taper	• Monitor changes of renal function
	Grade 3 or 4	Permanently discontinue		
Myocarditis	Grade 1	Withhold	• Based on severity of AE administer corticosteroids	• Ensure adequate evaluation to confirm etiology and/or exclude other causes
	Grade 2, 3 or 4	Permanently discontinue		
All Other irAEs	Persistent Grade 2	Withhold	• Based on severity of AE administer corticosteroids	• Ensure adequate evaluation to confirm etiology or exclude other causes
	Grade 3	Withhold or discontinue [b]		
	Recurrent Grade 3 or Grade 4	Permanently discontinue		

AE(s)=adverse event(s); ALT= alanine aminotransferase; AST=aspartate aminotransferase; CTCAE=Common Terminology Criteria for Adverse Events; DRESS=Drug Rash with Eosinophilia and Systemic Symptom; GI=gastrointestinal; IO=immuno-oncology; ir=immune related; IV=intravenous; SJS=Stevens-Johnson Syndrome; T1DM=type 1 diabetes mellitus; TEN=Toxic Epidermal Necrolysis; ULN=upper limit of normal.

Note: Non-irAE will be managed as appropriate, following clinical practice recommendations.

[a] The decision to withhold or permanently discontinue pembrolizumab monotherapy, coformulations or IO combinations is at the discretion of the investigator or treating physician. If control achieved or ≤ Grade 2, pembrolizumab monotherapy, coformulations or IO combinations may be resumed.

[b] Events that require discontinuation include, but are not limited to: Guillain-Barre Syndrome, encephalitis, myelitis, DRESS, SJS, TEN and other clinically important irAEs (eg, vasculitis and sclerosing cholangitis).

Dose Modification and Toxicity Management of Infusion/Injection Reactions Related to Pembrolizumab

Pembrolizumab may cause severe or life-threatening infusion/injection-reactions including severe hypersensitivity or anaphylaxis. Signs and symptoms usually develop during or shortly after drug infusion/injection and generally resolve completely within 24 hours of completion of infusion/injection. Dose modification and toxicity management guidelines on pembrolizumab associated infusion/injection reaction are provided in Table 7.

Table 7 Pembrolizumab Infusion/Injection Reaction Dose Modification and Treatment Guidelines

NCI CTCAE Grade	Treatment	Premedication at Subsequent Dosing
Grade 1<br>Mild reaction; infusion interruption not indicated; intervention not indicated	Increase monitoring of vital signs as medically indicated until the participant is deemed medically stable in the opinion of the investigator	None
Grade 2<br>Requires therapy or infusion interruption but responds promptly to symptomatic treatment (eg, antihistamines, NSAIDs, narcotics, IV fluids); prophylactic medications indicated for ≤24 hrs	Stop Infusion<br>Additional appropriate medical therapy may include but is not limited to:<br>IV fluids<br>Antihistamines<br>NSAIDs<br>Acetaminophen<br>Narcotics<br>Increase monitoring of vital signs as medically indicated until the participant is deemed medically stable in the opinion of the investigator.<br>If symptoms resolve within 1 hour of stopping drug infusion, the infusion may be restarted at 50% of the original infusion rate (eg, from 100 mL/hr to 50 mL/hr). Otherwise dosing will be held until symptoms resolve and the participant should be premedicated for the next scheduled dose.<br>Participants who develop Grade 2 toxicity despite adequate premedication should be permanently discontinued from further study drug treatment.	Participant may be premedicated 1.5 h (±30 minutes) prior to infusion/injection of _____ with:<br>Diphenhydramine 50 mg PO (or equivalent dose of antihistamine).<br>Acetaminophen 500-1000 mg PO (or equivalent dose of analgesic).

NCI CTCAE Grade	Treatment	Premedication at Subsequent Dosing
Grades 3 or 4<br>Grade 3:<br>Prolonged (ie, not rapidly responsive to symptomatic medication and/or brief interruption of infusion); recurrence of symptoms following initial improvement; hospitalization indicated for other clinical sequelae (eg, renal impairment, pulmonary infiltrates)<br>Grade 4:<br>Life-threatening; pressor or ventilatory support indicated	Stop Infusion.<br>Additional appropriate medical therapy may include but is not limited to:<br>Epinephrine**<br>IV fluids<br>Antihistamines<br>NSAIDs<br>Acetaminophen<br>Narcotics<br>Oxygen<br>Pressors<br>Corticosteroids<br>Increase monitoring of vital signs as medically indicated until the participant is deemed medically stable in the opinion of the investigator.<br>Hospitalization may be indicated.<br>**In cases of anaphylaxis, epinephrine should be used immediately.<br>Participant is permanently discontinued from further study drug treatment.	No subsequent dosing
Appropriate resuscitation equipment should be available at the bedside and a physician readily available during the period of drug administration.<br>For further information, please refer to the Common Terminology Criteria for Adverse Events v4.0 (CTCAE) at http://ctep.cancer.gov		

<u>Other Allowed Dose Interruption for Pembrolizumab</u>

Pembrolizumab may be interrupted for situations other than treatment-related AEs such as medical / surgical events or logistical reasons not related to study therapy. Participants should be placed back on study therapy within 3 weeks of the scheduled interruption, unless otherwise discussed with the Sponsor. The reason for interruption should be documented in the participant's study record.

6.7 Intervention After the End of the Study

There is no study-specified intervention following the end of the study.

6.8 Clinical Supplies Disclosure

This study is open-label; therefore, the participant, the study site personnel, the Sponsor, and/or designee are not blinded. Study intervention (pembrolizumab, 200 mg IV, 400 mg IV, and [redacted] SC) is included on the dispensed study medication label text; random code/disclosure envelopes or lists are not provided.

6.9 Standard Policies

Not applicable.

7 DISCONTINUATION OF STUDY INTERVENTION AND PARTICIPANT WITHDRAWAL

7.1 Discontinuation of Study Intervention

Discontinuation of study intervention does not represent withdrawal from the study.

As certain data on clinical events beyond study intervention discontinuation may be important to the study, they must be collected through the participant's last scheduled follow-up, even if the participant has discontinued study intervention. Therefore, all participants who discontinue study intervention prior to completion of the protocol-specified treatment period will still continue to participate in the study as specified in Section 1.3 and Section 8.11.3.

Participants may discontinue study intervention at any time for any reason or be dropped from the study intervention at the discretion of the investigator should any untoward effect occur. In addition, a participant may be discontinued from study intervention by the investigator or the Sponsor if study intervention is inappropriate, the study plan is violated, or for administrative and/or other safety reasons. Specific details regarding procedures to be performed at study intervention discontinuation are provided in Section 8.1.9.

A participant must be discontinued from study intervention but continue to be monitored in the study for any of the following reasons:

- The participant or participant's legally acceptable representative requests to discontinue study intervention.
- The participant interrupts study intervention administration for more than 12 consecutive weeks or has 3 cumulative missed doses unless Sponsor approval to continue.
- The participant has a medical condition or personal circumstance which, in the opinion of the investigator and/or Sponsor, placed the participant at unnecessary risk from continued administration of study intervention.
- The participant has a confirmed positive serum pregnancy test.
- Radiographic disease progression outlined in Section 8.11 (exception if the Sponsor approves treatment continuation following confirmed PD per iRECIST)
- Any progression or recurrence of any malignancy, or any occurrence of another malignancy that requires active treatment
- Unacceptable adverse experiences as described in Section 8.4.

- Intercurrent illness other than another malignancy as noted above that prevents further administration of treatment.
- Investigator's decision to discontinue treatment.
- Recurrent Grade 2 pneumonitis
- Completion of 35 treatments (Cohort A) or 18 treatments (Cohort B) (approximately 2 years) with pembrolizumab

For participants who are discontinued from study intervention all applicable discontinuation activities will be performed according to Section 1.3 (Schedule of Activities) and Section 8.1.9.

7.2 Participant Withdrawal From the Study

A participant must be withdrawn from the study if the participant or participant's legally acceptable representative withdraws consent from the study.

If a participant withdraws from the study, they will no longer receive study treatment or be followed at scheduled protocol visits.

Specific details regarding procedures to be performed at the time of withdrawal from the study, are outlined in Section 8.1.9. The procedures to be performed should a participant repeatedly fail to return for scheduled visits and/or if the study site is unable to contact the participant are outlined in Section 7.3.

7.3 Lost to Follow-up

If a participant fails to return to the clinic for a required study visit and/or if the site is unable to contact the participant, the following procedures are to be performed:

- The site must attempt to contact the participant and reschedule the missed visit. If the participant is contacted, the participant should be counseled on the importance of maintaining the protocol-specified visit schedule.
- The investigator or designee must make every effort to regain contact with the participant at each missed visit (eg, telephone calls and/or a certified letter to the participant's last known mailing address or locally equivalent methods). These contact attempts should be documented in the participant's medical record.
- Note: A participant is not considered lost to follow-up until the last scheduled visit for the individual participant. The missing data for the participant will be managed via the prespecified statistical data handling and analysis guidelines.

8 STUDY ASSESSMENTS AND PROCEDURES

- Study procedures and their timing are summarized in the SoA.
- Adherence to the study design requirements, including those specified in the SoA, is essential and required for study conduct.
- The investigator is responsible for ensuring that procedures are conducted by appropriately qualified or trained staff. Delegation of study site personnel responsibilities will be documented in the Investigator Trial File Binder (or equivalent).
- All study-related medical decisions must be made by an investigator who is a qualified physician.
- All screening evaluations must be completed and reviewed to confirm that potential participants meet all eligibility criteria. The investigator will maintain a screening log to record details of all participants screened and to confirm eligibility or record reasons for screening failure, as applicable.
- Procedures conducted as part of the participant's routine clinical management (eg, blood count) and obtained before signing of ICF may be utilized for screening or baseline purposes provided the procedure met the protocol-specified criteria and were performed within the time frame defined in the SoA.
- Additional evaluations/testing may be deemed necessary by the investigator and or the Sponsor for reasons related to participant safety. In some cases, such evaluation/testing may be potentially sensitive in nature (eg, HIV, Hepatitis C), and thus local regulations may require that additional informed consent be obtained from the participant. In these cases, such evaluations/testing will be performed in accordance with those regulations.

The maximum amount of blood collected from each participant over the duration of the study is outlined in the Procedures Manual.

Repeat or unscheduled samples may be taken for safety reasons or for technical issues with the samples.

8.1 Administrative and General Procedures

8.1.1 Informed Consent

The investigator or medically qualified designee (consistent with local requirements) must obtain documented consent from each potential participant or each participant's legally acceptable representative prior to participating in a clinical study. If there are changes to the participant's status during the study (eg, health or age of majority requirements), the investigator or medically qualified designee must ensure the appropriate consent is in place.

8.1.1.1 General Informed Consent

Consent must be documented by the participant's dated signature or by the participant's legally acceptable representative's dated signature on a consent form along with the dated signature of the person conducting the consent discussion.

A copy of the signed and dated consent form should be given to the participant before participation in the study.

The initial ICF, any subsequent revised written ICF, and any written information provided to the participant must receive the Institutional Review Board/Independent Ethics Committee's (IRB/IEC's) approval/favorable opinion in advance of use. The participant or his/her legally acceptable representative should be informed in a timely manner if new information becomes available that may be relevant to the participant's willingness to continue participation in the study. The communication of this information will be provided and documented via a revised consent form or addendum to the original consent form that captures the participant's dated signature or by the participant's legally acceptable representative's dated signature.

Specifics about a study and the study population will be added to the consent form template at the protocol level.

The informed consent will adhere to IRB/IEC requirements, applicable laws and regulations, and Sponsor requirements.

8.1.2 Inclusion/Exclusion Criteria

All inclusion and exclusion criteria will be reviewed by the investigator who is a qualified physician to ensure that the participant qualifies for the study.

8.1.3 Participant Identification Card

All participants will be given a participant identification card identifying them as participants in a research study. The card will contain study site contact information (including direct telephone numbers) to be used in the event of an emergency. The investigator or qualified designee will provide the participant with a participant identification card immediately after the participant provides written informed consent. At the time of intervention allocation/randomization, site personnel will add the intervention/randomization number to the participant identification card.

The participant identification card also contains contact information for the emergency unblinding call center so that a healthcare provider can obtain information about study intervention in emergency situations where the investigator is not available.

8.1.4 Medical History

A medical history will be obtained by the investigator or qualified designee. The medical history will collect all active conditions and any condition diagnosed within the prior

10 years that the investigator considers to be clinically significant. Any cancer other than the cancer under study will be recorded as medical history even if diagnosed greater than 10 years prior to enrollment. Details regarding the disease for which the participant has enrolled in this study will be recorded separately and not listed as medical history.

The investigator or qualified designee will obtain historic and current details of the participant's cancer under study. This information will include, but is not limited to, date of diagnosis, stage, histology, locations of primary lesions and location of metastases if applicable.

8.1.5 Prior and Concomitant Medications Review

8.1.5.1 Prior Medications

The investigator or qualified designee will review prior medication use, including any protocol-specified washout requirement, and record prior medication taken by the participant within 28 days before starting study intervention. Treatment for the disease for which the participant has enrolled in this study will be recorded separately and not listed as a prior medication.

The investigator or qualified designee will review and record all treatments for the cancer under study including systemic and local treatment, vaccinations, radiation, and surgeries. Additional information collected on these treatments will include, but is not limited to, dates of administration, reason for discontinuation, best response, and date of progression after each treatment as applicable.

8.1.5.2 Concomitant Medications

The investigator or qualified designee will record medication, if any, taken by the participant during the study through the Safety follow-up visit.

The investigator or qualified designee will review and record all new anti-cancer treatments for the cancer under study initiated after the participant discontinued from study treatment. Additional information collected on these treatments will include, but is not limited to, dates of administration, reason for discontinuation, best response, and date of progression after each treatment as applicable.

8.1.6 Assignment of Screening Number

All consented participants will be given a unique screening number that will be used to identify the participant for all procedures that occur prior to randomization/allocation. Each participant will be assigned only 1 screening number. Screening numbers must not be re-used for different participants.

Any participant who is screened multiple times will retain the original screening number assigned at the initial screening visit. Specific details on the screening/rescreening visit requirements are provided in Section 8.11.1.

The procedures for obtaining a screening number from the Sponsor are outlined in the procedure manual.

8.1.7 Assignment of Treatment/Randomization Number

All eligible participants will be randomly allocated and will receive a treatment/randomization number. The treatment/randomization number identifies the participant for all procedures occurring after treatment allocation/randomization. Once a treatment/randomization number is assigned to a participant, it can never be re-assigned to another participant.

A single participant cannot be assigned more than 1 treatment/randomization number.

The procedures for obtaining a randomization number from the Sponsor are outlined in the procedure manual.

8.1.8 Study Intervention Administration

Administration of study medication will be witnessed by the investigator and/or study staff.

Study treatment should begin within 3 days of randomization/allocation.

8.1.8.1 Timing of Dose Administration

Study treatment with pembrolizumab should be administered on Day 1 of each cycle after all procedures/assessments have been completed as detailed in the SoA (Section 1.3.1). All study treatments will be administered on an outpatient basis.

The Pharmacy Manual contains specific instructions for pembrolizumab storage, reconstitution, preparation of the infusion fluid, and administration. Note that the SC dose of [redacted] is approximate (A: [redacted] B: [redacted]).

Cohort A

Study treatment with pembrolizumab will be administered on the scheduled treatment day for Day 1 of Cycles 1, 2, and 3 (note: Cycle 1 dose should be administered within 3 days of randomization). For Cycles 4 and beyond, study treatment with pembrolizumab may be administered up to 3 days before or after the scheduled Day 1 of each cycle (eg, due to administrative reasons).

Pembrolizumab will be administered as a dose of either 200 mg using a 30-minute IV infusion or [redacted] injected SC. Sites should make every effort to target infusion timing to be as close to 30 minutes as possible. However, given the variability of infusion pumps from site to site, a window of −5 minutes and +10 minutes is permitted (ie, infusion time is 30 minutes −5 min/+10 min).

Cohort B

Study treatment with pembrolizumab may be administered up to 3 days before or after the scheduled Day 1 of each cycle (eg, due to administrative reasons) after Cycle 1. Dosing for Cycle 1 should be within 3 days of randomization. Pembrolizumab will be administered as a 400 mg dose using a 30-minute IV infusion. Sites should make every effort to target infusion timing to be as close to 30 minutes as possible. However, given the variability of infusion pumps from site to site, a window of −5 minutes and +10 minutes is permitted (ie, infusion time is 30 minutes −5 min/+10 min).

8.1.9 Discontinuation and Withdrawal

Participants who discontinue study intervention prior to completion of the treatment period should be encouraged to continue to be followed for all remaining study visits.

When a participant withdraws from participation in the study, all applicable activities scheduled for the final study visit should be performed (at the time of withdrawal). Any AEs that are present at the time of withdrawal should be followed in accordance with the safety requirements outlined in Section 8.4.

8.1.10 Participant Blinding/Unblinding

This is an open-label study; there is no blinding for this study.

8.1.11 Domiciling

Not applicable.

8.1.12 Calibration of Equipment

The investigator or qualified designee has the responsibility to ensure that any device or instrument used for a clinical evaluation/test during a clinical study that provides information about inclusion/exclusion criteria and/or safety or efficacy parameters shall be suitably

calibrated and/or maintained to ensure that the data obtained is reliable and/or reproducible. Documentation of equipment calibration must be retained as source documentation at the study site.

8.2 Efficacy/ Assessments

8.2.1 Tumor Imaging and Assessment of Disease

Cohort A and B

Tumor assessments will include all known or suspected disease sites. Imaging may include chest, abdomen, and pelvis computed tomography (CT) or magnetic resonance imaging (MRI) at baseline and when disease progression or brain metastases are suspected. Tumor imaging is strongly preferred to be acquired by CT. For chest, abdomen and pelvis, contrast-enhanced MRI may be used when CT with iodinated contrast is contraindicated, or when mandated by local practice. For the brain, MRI is the strongly preferred imaging modality.

The same imaging modality technique (ideally the same scanner, and consistent use of contrast) should be used in a participant throughout the study. Consistent use of imaging techniques will help to optimize the reproducibility of the assessment of existing and new tumor burden, and to improve the accuracy of the assessment of response or progression. Note: for the purposes of assessing tumor imaging, the term “investigator” refers to the local investigator at the site and/or the radiological reviewer at the site or at an offsite facility.

All scheduled images for all study participants will be reviewed by the investigator for disease progression. In addition, images (including those obtained via other modalities) that are obtained at an unscheduled time point to determine disease progression (as well as imaging obtained for other reasons, but that capture radiologic progression based on investigator assessment), should also be filed at the study site. Note: for the purposes of assessing tumor imaging, the term “investigator” refers to the local investigator at the site and/or the radiological reviewer at the site or at an offsite facility.

Cohort B

Confirmation of measurable disease based on RECIST 1.1 by BICR at Screening will be used to determine participant eligibility. Confirmation by the BICR that the participant’s imaging shows at least 1 lesion that is appropriate for selection as a target lesion per RECIST 1.1 is required prior to participant allocation.

All scheduled imaging for all study participants will be submitted to the iCRO. In addition, imaging that is obtained at an unscheduled time point, for any reason (including suspicion of progression or other clinical reason), should also be submitted to the iCRO if it shows progression, or if it is used to support a response assessment. All imaging acquired within the protocol-specified window (± 7 days) of time around a scheduled imaging visit can be classified as pertaining to that visit.

8.2.1.1 Initial Tumor Imaging

Cohort A

Initial tumor imaging at Screening must be performed within 28 days prior to the date of randomization. Any imaging obtained after Cycle 1 Day 1 of treatment cannot be included in the screening assessment. The site study team must review screening images to confirm the participant has measurable disease per RECIST 1.1. Tumor imaging performed as part of routine clinical management is acceptable for use as screening tumor imaging if it is of diagnostic quality and performed within 28 days prior to the date of randomization.

If brain imaging is performed to document the stability of existing metastases, MRI should be used if possible. If MRI is medically contraindicated, CT with contrast is an acceptable alternative.

Cohort B

Initial tumor imaging at Screening must be performed within 28 days prior to the date of first dose. Any imaging obtained after Cycle 1 Day 1 of treatment cannot be included in the screening assessment. The site study team must review screening images to confirm the participant has measurable disease per RECIST 1.1. The screening images must be submitted to the iCRO for confirmation of measurable disease per RECIST 1.1 for eligibility prior to allocation.

If brain imaging is performed to document the stability of existing metastases, MRI should be used if possible. If MRI is medically contraindicated, CT with contrast is an acceptable alternative.

8.2.1.2 Tumor Imaging During the Study

Imaging timing should follow calendar days and should not be adjusted for delays in cycle starts. Imaging should continue to be performed until disease progression is identified by the investigator and (unless the investigator elects to continue treatment and follow iRECIST), the start of new anticancer treatment, withdrawal of consent, or death, or notification by the Sponsor, whichever occurs first.

Objective response should be confirmed by a repeat imaging assessment. Tumor imaging to confirm PR or CR should be performed at least 4 weeks after the first indication of a response is observed. Participants will then return to regular scheduled imaging, starting with the next scheduled imaging time point. Participants who receive additional imaging for confirmation do not need to undergo the next scheduled tumor imaging if it is less than 4 weeks later; tumor imaging may resume at the subsequent scheduled imaging time point.

Per modified iRECIST (Section 8.2.1.5), disease progression should be confirmed by the site 4 to 8 weeks after first radiologic evidence of progressive disease (PD) in clinically stable participants. Participants who have unconfirmed disease progression may continue on

treatment at the discretion of the investigator until progression is confirmed by the site, provided they have met the conditions detailed in Section 8.2.1.5. Participants who receive confirmatory imaging do not need to undergo the next scheduled tumor imaging if it is less than 4 weeks later; tumor imaging may resume at the subsequent scheduled imaging time point, if clinically stable. Participants who have confirmed disease progression by iRECIST, as assessed by the site, will discontinue study treatment. Exceptions are detailed in Section 8.2.1.5.

Cohort A

The first on-study imaging assessment should be performed at approximately 12 weeks from the date of randomization. Subsequent tumor imaging should be performed per the institutional standard of care.

Cohort B

The first on-study imaging assessment should be performed at 12 weeks (84 days ± 7 days]) from the date of first dose. Subsequent tumor imaging should be performed every 9 weeks (63 days ± 7 days) or more frequently if clinically indicated. After 52 weeks (365 days ±7 days), participants who remain on treatment will have imaging performed every 12 weeks (84 days ± 7 days). All supplemental imaging must be submitted to the iCRO.

8.2.1.3 End-of-Treatment and Follow-up Tumor Imaging

For participants who discontinue study intervention, tumor imaging should be performed at the time of treatment discontinuation (± 4 week window). If previous imaging was obtained within 4 weeks prior to the date of discontinuation, then imaging at treatment discontinuation is not mandatory. For participants who discontinue study intervention due to documented disease progression, this is the final required tumor imaging if the investigator elects not to implement iRECIST.

For participants in Cohort B who discontinue study intervention without documented disease progression, every effort should be made to continue monitoring disease status by tumor imaging every 12 weeks until the start of a new anticancer treatment, disease progression, pregnancy, death, withdrawal of consent, or the end of the study, whichever occurs first.

8.2.1.4 RECIST 1.1 Assessment of Disease

RECIST 1.1 will be used (by BICR for Cohort B) as the primary measure for assessment of tumor response, date of disease progression, and as a basis for all protocol guidelines related to disease status (eg, discontinuation of study intervention). Although RECIST 1.1 references a maximum of 5 target lesions in total and 2 per organ, this protocol allows a maximum of 10 target lesions in total and 5 per organ, if clinically relevant to enable a broader sampling of tumor burden.

8.2.1.5 iRECIST Assessment of Disease

iRECIST is based on RECIST 1.1, but adapted to account for the unique tumor response seen with immunotherapeutic drugs. iRECIST will be used by the investigator to assess tumor response and progression, and make treatment decisions. When clinically stable, participants should not be discontinued until progression is confirmed by the investigator, working with local radiology, according to the rules outlined in Appendix 8. This allowance to continue treatment despite initial radiologic PD takes into account the observation that some participants can have a transient tumor flare in the first few months after the start of immunotherapy, and then experience subsequent disease response. This data will be captured in the clinical database.

Clinical stability is defined as the following:

- Absence of symptoms and signs indicating clinically significant progression of disease
- No decline in ECOG performance status
- No requirements for intensified management, including increased analgesia, radiation, or other palliative care

Any participant deemed clinically unstable should be discontinued from study intervention at the time when site-assessed first radiologic evidence of PD, and is not required to have repeat tumor imaging for confirmation of PD by iRECIST.

If the investigator decides to continue treatment, the participant may continue to receive study intervention and the tumor assessment should be repeated 4 to 8 weeks later to confirm PD by iRECIST, per investigator assessment. Images should continue to be sent in to the iCRO for potential retrospective BICR (Cohort B).

If repeat imaging does not confirm PD per iRECIST, as assessed by the investigator, and the participant continues to be clinically stable, study intervention may continue and follow the regular imaging schedule. If PD is confirmed, participants will be discontinued from study intervention.

If a participant has confirmed radiographic progression (iCPD) as defined in Appendix 8, study intervention should be discontinued; however, if the participant is achieving a clinically meaningful benefit, an exception to continue study intervention may be considered following consultation with the Sponsor. In this case, if study intervention is continued, tumor imaging should continue to be performed following the intervals as outlined in Section 8.2.1.2 and submitted to the iCRO (Cohort B).

A description of the adaptations and iRECIST process is provided in Appendix 8, with additional details in the iRECIST publication [Seymour, L., et al 2017]. A summary of imaging and treatment requirements after first radiologic evidence of progression is provided in Table 8 and illustrated as a flowchart in Figure 2.

Table 8 Imaging and Treatment after First Radiologic Evidence of Progressive Disease

	**Clinically Stable**		**Clinically Unstable**	
	**Imaging**	**Treatment**	**Imaging**	**Treatment**
First radiologic evidence of PD by RECIST 1.1 per investigator assessment	Repeat imaging at 4 to 8 weeks to confirm PD	May continue study treatment at the assessment of the investigator and after the participant's consent	Repeat imaging at 4 to 8 weeks to confirm PD per investigator's discretion only.	Discontinue treatment
First radiologic evidence of PD by RECIST 1.1	Repeat imaging at 4 to 8 weeks to confirm PD.	May continue study intervention at the investigator's discretion while awaiting confirmatory tumor imaging by site by iRECIST.	Repeat imaging at 4 to 8 weeks to confirm PD per investigator's discretion only.	Discontinue treatment
Repeat tumor imaging confirms PD (iCPD) by iRECIST per investigator assessment.	No additional imaging required.	Discontinue treatment (exception is possible upon consultation with Sponsor).	No additional imaging required.	Not applicable
Repeat tumor imaging shows iUPD by iRECIST per investigator assessment.	Repeat imaging at 4 to 8 weeks to confirm PD. May occur at next regularly scheduled imaging visit.	Continue study intervention at the investigator's discretion.	Repeat imaging at 4 to 8 weeks to confirm PD per investigator's discretion only.	Discontinue treatment
Repeat tumor imaging shows iSD, iPR, or iCR by iRECIST per investigator assessment.	Continue regularly scheduled imaging assessments.	Continue study intervention at the investigator's discretion.	Continue regularly scheduled imaging assessments.	May restart study intervention if condition has improved and/or clinically stable per investigator's discretion. Next tumor imaging should occur according to the regular imaging schedule.

Abbreviations: iCPD=iRECIST confirmed progressive disease; iCR=iRECIST complete response; iPR=iRECIST confirmed partial response; iRECIST=modified Response Evaluation Criteria in Solid Tumors 1.1 for immune-based therapeutics; iSD=iRECIST stable disease; iUPD=iRECIST unconfirmed progressive disease; PD=progressive disease; RECIST 1.1=Response Evaluation Criteria in Solid Tumors 1.1; VOP=verification of progression

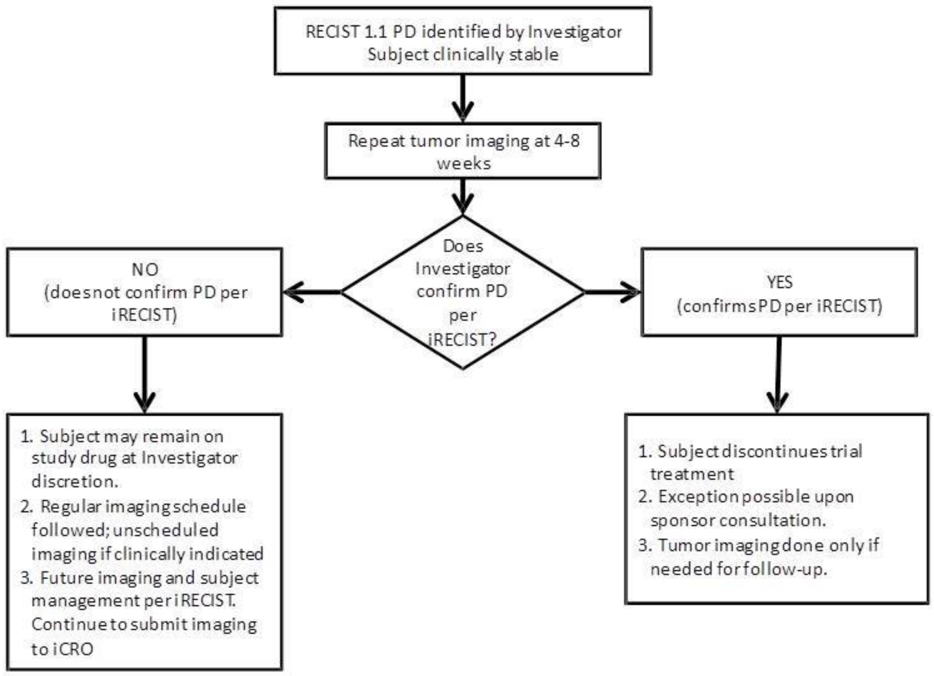

Figure 2 Imaging and Treatment for Clinically Stable Participants Treated With Pembrolizumab After First Radiologic Evidence of PD Assessed by the Investigators

Abbreviations: iRECIST=modified Response Evaluation Criteria in Solid Tumors 1.1 for immune-based therapeutics; PD=progressive disease; RECIST 1.1=Response Evaluation Criteria in Solid Tumors 1.1

Note: Imaging submission to iCRO is only applicable to Cohort B.

8.3 Safety Assessments

Safety assessments will include the collection of AEs and SAEs, monitoring of vital signs and laboratory assessments (including pregnancy tests), performance of electrocardiograms (ECGs) and physical examinations, and verification of concurrent medications.

Every effort should be made to ensure that the protocol required tests and procedures are completed as presented in the SoA.

Planned time points for all safety assessments are provided in the SoA.

8.3.1 Adverse Events

The investigator or qualified designee will assess each subject to evaluate for potential new or worsening AEs as specified in the SoA (Section 1.3) and more frequently if clinically indicated. Assessment of AEs will include, but is not limited to, the type, incidence, severity (graded by the National Cancer Institute Common Terminology Criteria for Adverse Events [NCI CTCAE] Version 4.0), timing, seriousness, and relatedness to study drug. Adverse events that occur during the study, including baseline signs and symptoms, will be recorded on the AE case report form (CRF) page.

8.3.2 Physical Examinations

8.3.2.1 Full Physical Examination

The investigator or qualified designee will perform a complete physical exam during the Screening period. Clinically significant abnormal findings should be recorded as medical history. The time points for full physical exams are described in Section 1.3. After the first dose of study intervention, new clinically significant abnormal findings should be recorded as AEs.

8.3.2.2 Directed Physical Examination

For cycles that do not require a full physical exam as defined in Section 1.3, the investigator or qualified designee will perform a directed physical exam as clinically indicated prior to the administration of the study intervention. New clinically significant abnormal findings should be recorded as AEs.

8.3.3 Vital Signs

The investigator or qualified designee will take vital signs at screening, prior to each pembrolizumab administration, after SC pembrolizumab administration, and during the follow-up period as specified in the SoA (Section 1.3).

Vital signs will be measured in a semi-supine position after 5 minutes rest and will include temperature, systolic and diastolic blood pressure, respiratory rate, pulse rate, and weight. Height will be collected at screening only.

8.3.4 Electrocardiograms

A standard 12-lead ECG will be performed using local standard procedures. The timing of ECGs is specified in the SoA in Section 1.3. Clinically significant abnormal findings at Screening should be recorded as medical history. Additional ECG(s) should be performed on study when clinically necessary. Clinically significant findings seen on the follow-up ECGs should be recorded as AEs.

8.3.5 Clinical Safety Laboratory Assessments

- Refer to Appendix 2 for the list of clinical laboratory tests to be performed and to the SoA for the timing and frequency.

- The investigator or medically qualified designee (consistent with local requirements) must review the laboratory report, document this review, and record any clinically relevant changes occurring during the study in the AE section of the case report form (CRF). The laboratory reports must be filed with the source documents. Clinically significant abnormal laboratory findings are those which are not associated with the underlying disease, unless judged by the investigator to be more severe than expected for the participant's condition.

- All protocol-required laboratory assessments, as defined in Appendix 2, must be conducted in accordance with the laboratory manual and the SoA.

- If laboratory values from nonprotocol specified laboratory assessments performed at the institution's local laboratory require a change in study participant management or are considered clinically significant by the investigator (eg, SAE or AE or dose modification), then the results must be recorded in the appropriate CRF (eg, SLAB).

- For any laboratory tests with values considered clinically significantly abnormal during participation in the study or within 30 days after the last dose of study intervention, every attempt should be made to perform repeat assessments until the values return to normal or baseline or if a new baseline is established as determined by the investigator.

Details regarding specific laboratory procedures/assessments to be performed in this study are provided below. The total amount of blood/tissue to be drawn/collected over the course of the study (from prestudy to poststudy visits), including approximate blood/tissue volumes drawn/collected by visit and by sample type per participant can be found in the Study Procedures Manual. Refer to the SoA (Section 1.3) for the timing of laboratory assessments.

8.3.5.1 Laboratory Safety Evaluations (Hematology, Chemistry and Urinalysis)

Laboratory tests for hematology, chemistry, and urinalysis are specified in Appendix 2.

Laboratory tests for screening should be performed within 72 hours prior to the first dose of study intervention. An exception is hepatitis and thyroid serologies, which may be performed within 28 days prior to first dose. After Cycle 1, predose laboratory safety tests can be conducted up to 72 hours prior to dosing unless otherwise noted on the SoA (Section 1.3).

Laboratory test results must be reviewed by the investigator or qualified designee and found to be acceptable prior to administration of each dose of study intervention. Unresolved abnormal laboratory values that are drug-related AEs should be followed until resolution.

Laboratory tests do not need to be repeated after the end of treatment if laboratory results are within normal range.

8.3.5.2 Pregnancy Test

All women who are being considered for participation in the study, and who are not surgically sterilized or postmenopausal, must be tested for pregnancy within 72 hours or the first dose of study intervention. If a urine test is positive or not evaluable, a serum test will be required. Participants must be excluded/discontinued from the study in the event of a positive test result. Repeated pregnancy test (such as monthly testing) may be conducted if required by local regulation.

8.3.6 Performance Assessments

8.3.6.1 Eastern Cooperative Oncology Group Performance Scale

The investigator or qualified designee will assess ECOG status (see Appendix 8) at screening (Cohort A and Cohort B). For Cohort A, repeat ECOG should be performed on-study only if clinically necessary. For Cohort B, ECOG should be performed every visit as specified in the SoA (Section 1.3).

8.3.7 Injection Site Signs and Symptoms Questionnaire

Participants will complete the injection site signs and symptoms questionnaire after SC injection of pembrolizumab during the first 3 treatment cycles in Cohort A as specified in the SoA (Section 1.3). The questionnaire should be completed 60 minutes after the SC injection.

8.4 Adverse Events (AEs), Serious Adverse Events (SAEs), and Other Reportable Safety Events

The definitions of an AE or SAE, as well as the method of recording, evaluating, and assessing causality of AE and SAE and the procedures for completing and transmitting AE, SAE, and other reportable safety event reports can be found in Appendix 3.

Progression of the cancer under study is not considered an AE unless it results in hospitalization or death as described in Section 8.4.6 and Appendix 3.

Adverse events, SAEs, and other reportable safety events will be reported by the participant (or, when appropriate, by a caregiver, surrogate, or the participant's legally authorized representative).

The investigator and any designees are responsible for detecting, documenting, and reporting events that meet the definition of an AE or SAE as well as other reportable safety events. Investigators remain responsible for following up AE, SAEs, and other reportable safety events for outcome according to Section 8.4.3.

The investigator, who is a qualified physician, will assess events that meet the definition of an AE or SAE as well as other reportable safety events with respect to seriousness, intensity/toxicity and causality.

8.4.1 Time Period and Frequency for Collecting AE, SAE, and Other Reportable Safety Event Information

All AEs, SAEs, and other reportable safety events that occur after the consent form is signed but before treatment allocation/randomization must be reported by the investigator if the participant is receiving placebo run-in or other run-in treatment, if the event cause the participant to be excluded from the study, or is the result of a protocol-specified intervention, including but not limited to washout or discontinuation of usual therapy, diet, or a procedure.

- All AEs from the time of treatment allocation/randomization through 30 days following cessation of study intervention must be reported by the investigator.

- All AEs meeting serious criteria, from the time of treatment allocation/randomization through 90 days following cessation of study intervention or 30 days following cessation of study intervention if the participant initiates new anticancer therapy, whichever is earlier, must be reported by the investigator.

- All pregnancies and exposure during breastfeeding, from the time of treatment allocation/randomization through 120 days following cessation of study intervention, or 30 days following cessation of study intervention if the participant initiates new anticancer therapy must be reported by the investigator.

- Additionally, any SAE brought to the attention of an investigator at any time outside of the time period specified above must be reported immediately to the Sponsor if the event is considered drug-related.

Investigators are not obligated to actively seek AE or SAE or other reportable safety events in former study participants. However, if the investigator learns of any SAE, including a death, at any time after a participant has been discharged from the study, and he/she considers the event to be reasonably related to the study intervention or study participation, the investigator must promptly notify the Sponsor.

All initial and follow-up AEs, SAEs, and other reportable safety events will be recorded and reported to the Sponsor or designee within the time frames as indicated in Table 9.

Table 9 Reporting Time Periods and Time Frames for Adverse Events and Other Reportable Safety Events

Type of Event	Reporting Time Period: Consent to Randomization/ Allocation	Reporting Time Period: Randomization/ Allocation through Protocol-Specified Follow-up Period	Reporting Time Period: After the Protocol Specified Follow-up Period	Timeframe to Report Event and Follow-up Information to Sponsor:
Non-Serious Adverse Event (NSAE)	Report if:<br>- due to protocol-specified intervention<br>- causes exclusion<br>- participant is receiving placebo run-in or other run-in treatment	Report all	Not required	Per data entry guidelines
Serious Adverse Event (SAE) including Cancer and Overdose	Report if:<br>- due to protocol-specified intervention<br>- causes exclusion<br>- participant is receiving placebo run-in or other run-in treatment	Report all	Report if:<br>drug-/vaccine-related.<br>(Follow ongoing to outcome)	Within 24 hours of learning of event
Pregnancy/Lactation Exposure	Report if:<br>- due to intervention<br>- causes exclusion	Report all	Previously reported – Follow to completion/termination; report outcome	Within 24 hours of learning of event
Event of Clinical Interest (require regulatory reporting)	Report if:<br>- due to intervention<br>- causes exclusion	Report<br>- Potential drug-induced liver injury (DILI)<br>- Require regulatory reporting	Not required	Within 24 hours of learning of event
Event of Clinical Interest (Do not require regulatory reporting)	Report if:<br>- due to intervention<br>- causes exclusion	Report<br>- non-DILI ECIs and those not requiring regulatory reporting	Not required	Within 5 calendar days of learning of event

8.4.2 Method of Detecting AEs, SAEs, and Other Reportable Safety Events

Care will be taken not to introduce bias when detecting AE and/or SAE and other reportable safety events. Open-ended and nonleading verbal questioning of the participant is the preferred method to inquire about AE occurrence.

8.4.3 Follow-up of AE, SAE, and Other Reportable Safety Event Information

After the initial AE/SAE report, the investigator is required to proactively follow each participant at subsequent visits/contacts. All AE, SAE, and other reportable safety events including pregnancy and exposure during breastfeeding, events of clinical interest (ECI), cancer, and overdose will be followed until resolution, stabilization, until the event is otherwise explained, or the participant is lost to follow-up (as defined in Section 7.3). In addition, the investigator will make every attempt to follow all nonserious AEs that occur in randomized participants for outcome. Further information on follow-up procedures is given in Appendix 3.

8.4.4 Regulatory Reporting Requirements for SAE

Prompt notification (within 24 hours) by the investigator to the Sponsor of SAE is essential so that legal obligations and ethical responsibilities towards the safety of participants and the safety of a study intervention under clinical investigation are met.

The Sponsor has a legal responsibility to notify both the local regulatory authority and other regulatory agencies about the safety of a study intervention under clinical investigation. All AEs will be reported to regulatory authorities, IRB/IECs, and investigators in accordance with all applicable global laws and regulations (ie, per ICH Topic E6 (R2) Guidelines for Good Clinical Practice [GCP]).

Investigator safety reports must be prepared for suspected unexpected serious adverse reactions (SUSARs) according to local regulatory requirements and Sponsor policy and forwarded to investigators as necessary.

An investigator who receives an investigator safety report describing an SAE or other specific safety information (eg, summary or listing of SAE) from the Sponsor will file it along with the IB and will notify the IRB/IEC, if appropriate according to local requirements.

8.4.5 Pregnancy and Exposure During Breastfeeding

Although pregnancy and infant exposure during breastfeeding are not considered AEs, any pregnancy or infant exposure during breastfeeding in a participant (spontaneously reported to the investigator or their designee), including the pregnancy of a male participant’s female partner, that occurs during the study are reportable to the Sponsor.

All reported pregnancies must be followed to the completion/termination of the pregnancy. Pregnancy outcomes of spontaneous abortion, missed abortion, benign hydatidiform mole, blighted ovum, fetal death, intrauterine death, miscarriage, and stillbirth must be reported as

serious events (Important Medical Events). If the pregnancy continues to term, the outcome (health of infant) must also be reported.

8.4.6 Disease-related Events and/or Disease-related Outcomes Not Qualifying as AEs or SAEs

Progression of the cancer under study is not considered a reportable event unless it results in hospitalization or death.

8.4.7 Events of Clinical Interest (ECIs)

Selected nonserious and SAEs are also known as ECIs and must be reported to the Sponsor.

Events of clinical interest for this study include:

1. An overdose of Sponsor's product, as defined in Section 8.5, that is not associated with clinical symptoms or abnormal laboratory results.

2. An elevated AST or ALT lab value that is greater than or equal to 3X the upper limit of normal and an elevated total bilirubin lab value that is greater than or equal to 2X the upper limit of normal and, at the same time, an alkaline phosphatase lab value that is less than 2X the upper limit of normal, as determined by way of protocol-specified laboratory testing or unscheduled laboratory testing.*

   *Note: These criteria are based upon available regulatory guidance documents. The purpose of the criteria is to specify a threshold of abnormal hepatic tests that must trigger an additional evaluation for an underlying etiology. The study site guidance for assessment and follow up of these criteria can be found in the Investigator Study File Binder (or equivalent).

8.4.8 Medical Device Incidents (Including Malfunctions)

Not applicable.

8.5 Treatment of Overdose

For this study, an overdose of pembrolizumab will be defined as any dose of 1000 mg or greater.

No specific information is available on the treatment of overdose of pembrolizumab. In the event of overdose, the participant should be observed closely for signs of toxicity. Appropriate supportive treatment should be provided if clinically indicated.

8.6 Pharmacokinetics

To evaluate pembrolizumab immunogenicity and pembrolizumab exposure after MK-3475 SC administration (Cohort A), and Q6W dosing (Cohort B), sample collections for analysis

of ADAs and PK are currently planned as shown in the SoA (Section 1.3). Blood samples will be obtained to measure PK of serum pembrolizumab. The pembrolizumab serum concentration at planned visits and times will be summarized.

8.6.1 Blood Collection for Plasma for Pembrolizumab Pharmacokinetics and Immunogenicity

Sample collection, storage, and shipment instructions for plasma samples will be provided in the procedures/laboratory manual.

8.6.1.1 Blood Collection for Pharmacokinetics

Sample collection, storage, and shipment instructions for serum samples will be provided in the procedure/laboratory manual. Pharmacokinetic samples should be drawn according to the PK collection schedule for all participants.

8.6.1.2 Blood Collection for Anti-pembrolizumab Antibodies

Sample collection, storage, and shipment instructions for serum samples will be provided in the procedure manual. Anti-pembrolizumab antibody samples should be drawn according to the ADA collection schedule for all participants (Section 1.3).

8.7 Pharmacodynamics

Pharmacodynamic parameters will not be evaluated in this study.

8.8 Future Biomedical Research Sample Collection

Future biomedical research samples will not be collected in this study.

8.9 Planned Genetic Analysis Sample Collection

Planned genetic analysis samples will not be evaluated in this study.

8.10 Biomarkers

Biomarkers are not evaluated in this study.

8.10.1 RNA Transcriptome Research

Not applicable.

8.10.2 RNA Expression Research of a Subset of RNA Species

Not applicable.

8.10.3 Proteome Research

Not applicable.

8.10.4 Metabolomic Research

Not applicable.

8.11 Visit Requirements

Visit requirements are outlined in Section 1.3. Specific procedure-related details are provided in Section 8.

8.11.1 Screening

Written consent must be obtained prior to performing any protocol-specific procedure. Results of a test performed prior to the participant signing consent as part of routine clinical management are acceptable in lieu of a screening test if performed within the specified time frame. Screening procedures are to be completed within 28 days prior to the first dose of study intervention except for the following:

- Laboratory tests are to be performed within 72 hours prior to the first dose of study intervention. An exception is hepatitis and thyroid testing which may be done up to 28 days prior to the first dose of study intervention.
- Evaluation of ECOG is to be performed within 7 days prior to the first dose of study intervention.
- For women of reproductive potential, a urine or serum pregnancy test will be performed within 72 hours prior to the first dose of study intervention. If urine pregnancy results cannot be confirmed as negative, a serum pregnancy test will be required (performed by the local study site laboratory).

Participants may be rescreened after initially failing to meet the inclusion/exclusion criteria. Results from assessments during the initial screening period are acceptable in lieu of a repeat screening test if performed within the specified time frame and the corresponding inclusion/exclusion criteria is met. Participants who are rescreened will retain their original screening number.

8.11.2 Treatment Period

Visit requirements are outlined in the SoA (Section 1.3). Specific procedure-related details are provided in Section 8.1.

8.11.3 Discontinued Participants Continuing to be Monitored in the Study

The End of Treatment visit should occur at the time study treatment is discontinued for any reason. Visit requirements are outlined in the SoA (Section 1.3).

If the End of Treatment visit occurs 30 days from the last dose of study treatment, at the time of the mandatory Safety follow-up visit, the End of Treatment visit procedures and any additional safety follow up procedures should be performed.

8.11.3.1 Follow-up Visits

Cohort B

Participants who discontinue study treatment for a reason other than disease progression will move into the Follow-Up Phase and should be assessed every 12 weeks ([84] days ± 7 days) to monitor disease status. Every effort should be made to collect information regarding disease status until the start of new anti-cancer therapy, disease progression, death, end of study.

8.11.3.2 Safety Follow-up Visit

Participants will be required to return to the clinic for the Safety follow-up visit approximately 30 days after the last dose of study treatment or before initiating a new anticancer therapy, whichever occurs first. If the Safety follow-up visit occurs less than 30 days after the last dose of study treatment, a subsequent follow up phone call should be made 30 calendar days after the last dose of study treatment to determine if any AEs occurred since the last study visit.

After treatment discontinuation, participants will be monitored for AEs for 30 calendar days and SAEs for 90 calendar days after the last administration of the investigational product (30 calendar days if the participant initiates new anticancer therapy). Participants with an ongoing AE at the time of treatment discontinuation will be followed until resolution, stabilization, the event is otherwise explained, or the participant is lost to follow-up.

If the End of Treatment visit occurs 30 days from the last dose of study treatment, at the time of the mandatory Safety follow-up visit, the End of Treatment visit procedures and any additional safety follow up procedures should be performed.

8.11.3.3 Survival Follow-up

Cohort A

Survival follow-up will not be collected for participants in Cohort A.

Cohort B

Participants who experience confirmed disease progression or start a new anticancer therapy, will move into the Survival follow-Up Phase and should be contacted by telephone approximately every 12 weeks to assess for survival status until death, withdrawal of consent, or the end of the trial, whichever occurs first.

8.11.4 Survival Status

Cohort A

Collection of survival status will not be required for Cohort A.

Cohort B

To ensure current and complete survival data is available at the time of database locks, updated survival status may be requested during the course of the study by the Sponsor. For example, updated survival status may be requested prior to but not limited to an interim and/or final analysis. Upon Sponsor notification, all participants who do not/will not have a scheduled study visit or study contact during the Sponsor defined time period will be contacted for their survival status (excluding participants that have a previously recorded death event in the collection tool).

9 STATISTICAL ANALYSIS PLAN

9.1 Statistical Analysis Plan Summary

This section outlines the statistical analysis strategies and procedures for the primary, secondary, and exploratory analyses of the study.

If, after the study has begun, changes are made to primary and/or secondary objectives, or the statistical methods related to those objectives, then the protocol will be amended (consistent with ICH Guideline E9). Post hoc exploratory analyses will be clearly identified in the Clinical Study Report (CSR).

Full details are in the Statistical Analysis Plan, Section 9.2 through Section 9.12.

**Study Design Overview**	Phase 1, relative bioavailability, cross-over study of pembrolizumab administered via IV infusion or SC injection in participants with advanced melanoma (Cohort A) and of IV pembrolizumab Q6W dosing (Cohort B).
**Treatment Assignment**	In Cohort A, participants will be randomized to 1 of 6 treatment arms and will receive 1 IV infusion and 2 SC injections over the first 3 cycles, followed by IV infusion of pembrolizumab for up to 35 cycles.<br>In Cohort B, participants will receive IV infusion of pembrolizumab Q6W for up to 18 cycles.<br>Enrollment into Cohort A takes priority over Cohort B.
**Analysis Populations**	**Cohort A:**<br>PK (Primary): Per-Protocol (PP)<br>Safety (Secondary): All-Participants-as-Treated (APaT)<br>**Cohort B:**<br>Efficacy (Primary): Full Analysis Set (FAS)<br>PK (Secondary): Per-Protocol (PP)<br>Safety (Secondary): All-Participants-as-Treated (APaT)
**Primary Endpoint(s)**	**Cohort A:** Pharmacokinetic parameters of pembrolizumab, including area under the curve (AUC), maximum concentration (C_{max}), bioavailability (F), absorption rate (Ka), time of maximum concentration (T_{max}), clearance (CL), and central volume of distribution (Vc)<br>**Cohort B:** ORR per RECIST 1.1. by BICR
**Secondary Endpoints**	**Cohort A:**<br>• Circulating antidrug antibody levels<br>• Adverse events (AEs)<br>• Discontinuing study treatment due to an AE<br>• Responses to the injection site signs and symptoms questionnaire<br>**Cohort B:**<br>• DOR per RECIST 1.1 by BICR<br>• PFS per RECIST 1.1 by BICR

	• OS<br>• Adverse events (AEs)<br>• Pharmacokinetics: Pharmacokinetic parameters of pembrolizumab, including AUC, C_{max}, and C_{min} after administration of 400 mg Q6W pembrolizumab
**Exploratory**	**Cohort B**<br>• Circulating antidrug antibody levels
**Statistical Methods for Pharmacokinetic Analyses**	**Cohort A:** The relative bioavailability of pembrolizumab via SC injection will be estimated using AUC comparing with that via IV infusion. The log-transformed AUC of pembrolizumab will be analyzed with a linear mixed effects model with fixed effects terms for treatment and cycle. Point estimates for Geometric Mean Ratio (GMR, SC [redacted] versus IV, SC [redacted] versus IV) and their 90% confidence intervals (CIs) will also be calculated.
**Statistical Methods for Safety Analyses**	Summary statistics will be provided for the safety endpoints as appropriate.
**Statistical Methods for Efficacy Analyses**	**Cohort B:** Estimation of ORR; Estimation of DOR, PFS and OS using the Kaplan-Meier method.
**Interim Analyses**	No interim analysis is planned. Two PK analyses will be conducted during the study. The first PK analysis will occur when at least 30 participants have completed the first 3 cycles of this study in Cohort A. The second PK analysis will occur after Cycle 5 in Cohort B.
**Multiplicity**	No multiplicity adjustment is planned for this Phase 1 research design.
**Sample Size and Power**	There is no hypothesis testing. For Cohort A, assuming the true GMR of AUC between SC injection and IV infusion is 1.0, with 30 completers the 90% CI of the GMR will be (0.90, 1.11). For Cohort B, assuming the observed ORR is what is expected with pembrolizumab 200 mg Q3W for 35 cycles (~35%), 100 subjects will assure a 95% CI for the true ORR of approximately (25%, 45%).
Abbreviations: BICR=blinded independent central review; DOR=duration of response; ORR=objective response rate; OS=overall survival; PFS=progression-free survival; RECIST 1.1=Response Evaluation Criteria in Solid Tumors 1.1	

9.2 Responsibility for Analyses/In-house Blinding

The statistical analyses of the data obtained from this study will be the responsibility of the Clinical Biostatistics department of the Sponsor.

The study is open-label; participants, investigators, and Sponsor personnel will be aware of participant treatment assignment after each participant is enrolled and treatment is assigned. Allocation to treatment in Cohort A will be randomized. There will be direct treatment assignment for Cohort B.

9.3 Hypotheses/Estimation

Objectives of the study are outlined in Section 3.0. No hypotheses are being tested in this study.

9.4 Analysis Endpoints

9.4.1 Pharmacokinetics Endpoints (Cohorts A and B)

Pharmacokinetic endpoints are PK parameters of pembrolizumab after IV (200 mg Q3W) and SC administration including area under the curve (AUC), maximum concentration (C_{max}), bioavailability (F), absorption rate (Ka), time of maximum concentration (T_{max}), clearance (CL), and central volume of distribution (Vc). PK endpoints also include AUC, C_{max}, and C_{min} after IV infusion 400 mg Q6W.

In addition, the development of circulating anti-pembrolizumab antibodies (if applicable) will be evaluated following IV 200 mg Q3W and SC administration, as well as IV 400 mg Q6W pembrolizumab administration.

9.4.2 Safety Endpoints (Cohorts A and B)

Safety and tolerability will be assessed by clinical review of all relevant parameters including AEs, laboratory tests, and vital signs, as well as the injection site signs and symptoms questionnaire.

A description of safety measures is provided in Section 8.3 and Section 8.4.

9.4.3 Efficacy Endpoints (Cohort B)

9.4.3.1 Primary

Objective Response Rate (ORR) per RECIST 1.1 assessed by BICR

Objective Response is defined as a best overall response of CR or PR. The details of the ORR analysis plan can be found in Section 9.6.1.1.

9.4.3.2 Secondary

Duration of Response (DOR) per RECIST 1.1 assessed by BICR

For participants who demonstrate CR or PR, DOR is defined as the time from first documented evidence of CR or PR until disease progression or death due to any cause, whichever occurs first. See Section 9.6.1.4 for definition of censoring.

Progression-free Survival (PFS) per RECIST 1.1 assessed by BICR

PFS is defined as the time from the first day of study treatment to the first documented disease progression or death due to any cause, whichever occurs first. See Section 9.6.1.2 for definition of censoring.

Overall Survival (OS)

OS is defined as the time from the first day of study treatment to death due to any cause. Participants without documented death at the time of the final analysis will be censored at the date of the last follow-up.

9.5 Analysis Populations

9.5.1 Pharmacokinetic Analysis Population (Cohorts A and B)

The Per-Protocol (PP) population will be used for the analysis of PK data in this study. The PP population consists of the subset of participants who complied with the protocol sufficiently to ensure that their data will be likely to exhibit the effects of treatment, according to the underlying scientific model. Compliance includes such considerations as exposure to treatment, availability of measurements, and the absence of major protocol violations. Any participants or data values excluded from the analyses will be identified, along with the reasons for exclusion, in the CSR. At the end of the study, all participants who were compliant with the study procedures and have available data from at least 1 treatment will be included in the PP analysis dataset.

9.5.2 Safety Analysis Population (Cohorts A and B)

The All-Participants-as-Treated (APaT) population will be used for the analysis of safety data in this study, including injection site signs and symptoms. The APaT population consists of all participants who received at least 1 dose of study treatment. In case of treatment administration errors, participants will be analyzed according to the treatment they actually received.

At least 1 laboratory or vital sign measurement obtained subsequent to at least 1 dose of study treatment is required for inclusion in the analysis of each specific parameter. To assess change from baseline, a baseline measurement is also required.

9.5.3 Efficacy Analysis Population (Cohort B)

The APaT population will serve as the primary population for the analysis of efficacy data in this study.

9.6 Statistical Methods

9.6.1 Statistical Methods for Pharmacokinetic and Antidrug Antibodies Data (Cohorts A and B)

Cohort A

Primary/final PK analysis will be conducted when at least 30 participants have completed the first 3 cycles of this study. Along with the safety data, the PK analysis will be used for programmatic decision making purposes. The study will not be discontinued regardless of the outcome of the PK analysis.

The relative bioavailability of pembrolizumab via SC injection will be estimated using AUC comparing with that via IV infusion. The log-transformed AUC of pembrolizumab will be analyzed with a linear mixed effects model with fixed effects terms for treatment and cycle. An unstructured covariance matrix will be used to allow for unequal treatment variances and to model the correlation between different treatment measurements within the same subject via the REPEATED statement in SAS PROC MIXED. Kenward and Roger's method will be used to calculate the denominator degrees of freedom for the fixed effects (DDFM=KR). If the model fails to converge, a simpler covariances structure, such as heterogeneous-compound symmetry, will be used.

If the concentration of pembrolizumab observed in the pre-dose sample of Treatment Cycle 2 or Cycle 3 is <5% of the C_{max} observed in the same cycle, this level of carry-over can be considered negligible and no correction is needed. However, if the concentration observed in the pre-dose samples is ≥5% of the C_{max} observed in the same cycle, all concentrations in that cycle will be adjusted (assuming linear PK) for the impact of the carry-over as specified by the equation below. If carry-over adjustment is necessary in any given cycle, the adjustment will be made for all subjects with quantifiable predose concentrations before

non-compartmental PK analysis for AUC and statistical model fitting. In addition, a reduced model may be explored to fit the Treatment Cycle 1 data only. In this model treatment will be included as a factor. The key covariates will also be considered.

Cadj = Corig – CPD * e-(Ke)(t)

Where:

Cadj = carry-over-adjusted concentration

Corig = observed concentration

CPD = original predose concentration

Ke = slope of terminal phase (elimination constant lambda z in Phoenix) of the previous cycle

t = time of Corig compared to predose

Back-transformed Geometric Mean of AUC as well as its 95% CI will be calculated by treatment. Point estimates for Geometric Mean Ratio (GMR, SC [redacted] versus IV, SC [redacted] versus IV) and their 90% confidence intervals (CIs) will also be calculated. Other PK parameters including Ka, C_{max}, CL, and Vc will be analyzed in a similar fashion.

Antidrug antibodies data will be summarized by treatment and visit. Relationship between the presence/absence of positivity for ADA and PK of pembrolizumab will be explored.

Cohorts A and B

Individual values will be listed for each PK parameter by treatment, and the following (nonmodel-based) descriptive statistics will be provided: N (number of subjects with nonmissing data), arithmetic mean, standard deviation, arithmetic percent coefficient of variation (CV) (calculated as 100 x standard deviation/arithmetic mean), median, minimum, maximum, geometric mean, and geometric percent CV (calculated as 100 x sqrt(exp(s^2) - 1), where s^2 is the observed variance on the natural log-scale).

9.6.2 Statistical Methods for Efficacy Analyses (Cohort B)

This section describes the statistical methods that address the primary and secondary objectives.

9.6.2.1 Objective Response Rate (ORR)

ORR will be calculated as the ratio of the number of participants reported to have achieved a confirmed CR or PR verified by BICR, divided by the number of participants included in APaT population. Participants in the APaT analysis population without ORR assessments will be counted as non-responders.

A 95% exact binomial CI (based on method Clopper and Pearson,1934) will be calculated for the true ORR.

9.6.2.2 Progression-free Survival (PFS)

The non-parametric Kaplan-Meier method will be used to estimate the PFS distribution. 95% CIs for the median PFS and PFS point estimates at various follow-up times from first day of study treatment will be calculated.

Since disease progression is assessed periodically, PD can occur any time in the time interval between the last assessment where PD was not documented and the assessment when PD is documented. The true date of PD will be approximated by the date of the first assessment at which PD is objectively documented based on RECIST 1.1 by BICR. Death is always considered as a PFS event. Participants who do not experience a PFS event will be censored at the last disease assessment.

For the analysis of PFS, if the events (PD or death) are immediately after more than one missed disease assessment, the data are censored at the last disease assessment prior to missing visits. Also, data after new anticancer therapy are censored at the last disease assessment prior to the initiation of new anticancer therapy. The censoring rule is summarized in Table 10. If a participant meets multiple criteria for censoring, the censoring criterion that occurs earliest will be applied.

Table 10 Censoring Rules for Analysis of Progression-free Survival

Situation	Date of Progression or Censoring
PD or death documented after ≤1 missed disease assessment, and before new anticancer therapy, if any	Progressed at date of documented PD or death
PD or death documented immediately after ≥2 consecutive missed disease assessments or after new anticancer therapy, if any	Censored at last disease assessment prior to the earlier date of ≥2 consecutive missed disease assessment and new anticancer therapy, if any
No PD and no death; and new anticancer treatment is not initiated	Censored at last disease assessment
No PD and no death; new anticancer treatment is initiated	Censored at last disease assessment before new anticancer treatment
Abbreviations: PD=progressive disease; PFS=progression-free survival	

9.6.2.3 Overall Survival (OS)

The non-parametric Kaplan-Meier method will be used to estimate the OS distribution. 95% CIs for the median OS and OS point estimates at various follow-up times from first day of study treatment will be calculated.

9.6.2.4 Duration of Response (DOR)

DOR will be summarized descriptively using the non-parametric Kaplan-Meier method. Only the subset of participants who show a CR or PR will be included in this analysis. Censoring rules for DOR are summarized in Table 11.

Table 11 Censoring Rules for Duration of Response

Situation	Date of Progression or Censoring	Outcome
No progression nor death, no new anticancer therapy initiated	Last adequate disease assessment	Censor (non-event)
No progression nor death, new anticancer therapy initiated	Last adequate disease assessment before new anti-cancer therapy initiated	Censor (non-event)
Death or progression immediately after ≥ 2 consecutive missed disease assessments or after new anti-cancer therapy, if any	Earlier date of last adequate disease assessment prior to ≥ 2 missed adequate disease assessments and new anti-cancer therapy, if any	Censor (non-event)
Death or progression after ≤1 missed disease assessments and before new anti-cancer therapy, if any	Progressive disease or death	End of response (Event)
Note: A missed disease assessment includes any assessment that is not obtained or is considered inadequate for evaluation of response.<br>DOR = duration of response		

9.6.2.5 Analysis Strategy for Key Efficacy Endpoint

Table 12 summarizes the primary analysis approach for key efficacy endpoints.

Table 12 Analysis Strategy for Key Efficacy Endpoints

Endpoint	Statistical Method[a]	Analysis Population	Missing Data Approach
**Primary Endpoints**			
ORR per RECIST 1.1 by BICR	Exact method based on binomial distribution (Clopper-Pearson method)	APaT	Participants without assessments are considered non-responders and conservatively included in the denominator
**Key Secondary Endpoint**			
PFS per RECIST 1.1 by BICR	Summary statistics using Kaplan-Meier method	APaT	• Primary censoring rule<br>• Sensitivity analysis 1<br>• Sensitivity analysis 2<br>(More details are provided in Table 10, Censoring Rules for Primary and Sensitivity Analyses of PFS)
OS	Summary statistics using Kaplan-Meier method	APaT	Censored at the last known alive date
DOR per RECIST 1.1 by BICR	Summary statistics using Kaplan-Meier method	APaT	Non-responders are excluded from analysis. Responders are censored according to the censoring rules listed in Table 11.

[a] Statistical models are described in further detail in the text.

Abbreviations: APaT=All Participants as Treated; BICR=blinded independent central review; DOR=duration of response; ORR=objective response rate; OS=overall survival; PFS=progression-free survival; RECIST=Response Evaluation Criteria in Solid Tumors

9.6.3 Statistical Methods for Safety Analysis (Cohort A)

Safety and tolerability will be assessed by clinical review of all relevant parameters including AEs, SAEs, laboratory tests, vital signs, and physical examinations.

<u>Cycles 1 through 3</u>

The counts and frequency of AEs including injection site reaction AEs will be summarized by treatment and by sequence. In addition, AEs will be summarized based on Treatment Cycle 1 data only as well. Laboratory tests, vital signs, and other safety endpoints will be summarized as appropriate.

Descriptive summary statistics will be provided for the injection site signs and symptoms questionnaire data by treatment.

Post Cycle 3

Adverse events collected from Cycle 4 to the end of the study will reported.

9.6.4 Statistical Methods for Safety Analyses (Cohort B)

Safety and tolerability will be assessed by clinical review of all relevant parameters including adverse experiences and laboratory parameters.

The broad AE categories consisting of the percentage of participants with any AE, a drug-related AE, a serious AE, an AE which is both drug-related and serious, and who discontinued due to an AE will be summarized via point estimates with 95% CIs (Table 13).

Table 13 Analysis Strategy for Safety Parameters

Safety Endpoint	Within Group 95% CI	Descriptive Statistics
Any AE	X	X
Any Serious AE	X	X
Any Drug-related AE	X	X
Any Serious and Drug-related AE	X	X
Discontinuation due to AE	X	X
Specific AEs, SOCs, or PDLCs		X
Change from Baseline Results (Labs, Vital Signs)		X

Note: 95% CIs will be calculated using the Clopper Pearson method
X = results will be provided
Abbreviations: SOC=System Organ Class; PDLC=Pre-Defined Limit of Change

For continuous measures such as changes from baseline in laboratory parameters, summary statistics for baseline, on-treatment, and change from baseline values will be provided in table format.

9.6.5 Demographics and Baseline Characteristics (Cohort B)

The number and percentage of subjects screened, allocated, the primary reasons for screening failure, and the primary reasons for discontinuation will be displayed. Demographic variables (e.g., age, gender), baseline characteristics, primary and secondary diagnoses, and prior and concomitant therapies will be summarized either by descriptive statistics or categorical tables for all enrolled subjects.

9.7 Interim Analyses

No interim analysis is planned.

9.8 Multiplicity

No multiplicity adjustment is planned for this study.

9.9 Sample Size and Power Calculations

Cohort A

The overall sample size for this Phase 1 study is expected to be up to approximately 30 to 36 participants. Assuming that the within-subject standard deviation of pembrolizumab AUC in melanoma patients following subcutaneous administration is the same as it is following IV administration (which is 0.25 on log scale based on previous pembrolizumab studies) and assuming the true GMR of AUC between SC injection and IV infusion is 1.0, with 30 completers the 90% CI of GMR will be (0.90, 1.11).

A between-subject comparison will also be provided based on Treatment Cycle 1 data only. The between-subject standard deviation of pembrolizumab AUC in melanoma patients following IV administration is 0.30 on log scale based on previous pembrolizumab studies. Assuming the between-subject standard deviation following SC administration is 0.50 on log scale based on published SC PK data for other mAbs [Dirks, N. L. 2010], when the true GMR of AUC between SC injection and IV infusion is 1.0, with 10 completers in each treatment (30 in total), the 90% CI of GMR will be (0.72, 1.38).

Cohort B

Approximately 100 participants will be enrolled in Cohort B. The ORR for the dose and schedule to be used in Cohort B is expected to be similar to that seen with pembrolizumab, 200 mg Q3Ws 35 cycles (~ 35%). If the observed ORR for the 100 subjects was 35%, a 95% CI for the true ORR based on the method of Clopper and Pearson (1934) would be (25.7%, 45.2%).

9.10 Subgroup Analyses

Cohort A

There will be no subgroup analyses conducted for Cohort A.

Cohort B

To determine whether the response rate is consistent across various subgroups, the estimate of the response rate (with a nominal 95% CI) for the primary endpoint will be estimated within each category of the following classification variables:

- Age category (< 65 vs. ≥ 65 years)
- Sex (female vs. male)

- Race (white vs. non-white)
- Disease stage (III vs. IVM1a vs. IVM1b vs. IVM1c)
- Brain metastasis (yes vs. no)
- ECOG status (0 vs. 1)
- PD-L1 status (positive vs. negative [includes indeterminate])
- BRAF wild type versus BRAF mutant (no prior treatment) versus BRAF mutant (prior treatment)
- LDH (≤ ULN vs. > ULN but < 2X ULN vs. ≥ 2X ULN)

A Forest plot will be produced, which provides the estimated point estimates and CIs for the treatment effect across the categories of subgroups listed above.

Any specified subgroups that have less than 10 participants will be excluded from analysis.

9.11 Compliance (Medication Adherence)

Drug accountability data for study treatment will be collected during the study. Any deviation from protocol-directed administration will be reported.

9.12 Extent of Exposure

The extent of exposure will be summarized as duration of treatment in cycles.

10 SUPPORTING DOCUMENTATION AND OPERATIONAL CONSIDERATIONS

10.1 Appendix 1: Regulatory, Ethical, and Study Oversight Considerations

10.1.1 Code of Conduct for Clinical Trials

Merck Sharp and Dohme Corp., a subsidiary of Merck & Co., Inc. (MSD)

Code of Conduct for Interventional Clinical Trials

I. Introduction

A. Purpose

MSD, through its subsidiaries, conducts clinical trials worldwide to evaluate the safety and effectiveness of our products. As such, we are committed to designing, implementing, conducting, analyzing and reporting these trials in compliance with the highest ethical and scientific standards. Protection of participants in clinical trials is the overriding concern in the design of clinical trials. In all cases, MSD clinical trials will be conducted in compliance with local and/or national regulations (eg, International Council for Harmonisation Good Clinical Practice [ICH-GCP]) and in accordance with the ethical principles that have their origin in the Declaration of Helsinki.

B. Scope

Highest ethical and scientific standards shall be endorsed for all clinical interventional investigations sponsored by MSD irrespective of the party (parties) employed for their execution (eg, contract research organizations, collaborative research efforts). This Code is not intended to apply to trials that are observational in nature, or which are retrospective. Further, this Code does not apply to investigator-initiated trials, which are not under the full control of MSD.

II. Scientific Issues

A. Trial Conduct

1. Trial Design

Except for pilot or estimation trials, clinical trial protocols will be hypothesis-driven to assess safety, efficacy, and/or pharmacokinetic or pharmacodynamic indices of MSD or comparator products. Alternatively, MSD may conduct outcomes research trials, trials to assess or validate various endpoint measures, or trials to determine patient preferences, etc.

The design (ie, participant population, duration, statistical power) must be adequate to address the specific purpose of the trial. Participants must meet protocol entry criteria to be enrolled in the trial.

2. Site Selection

MSD selects investigative sites based on medical expertise, access to appropriate participants, adequacy of facilities and staff, previous performance in clinical trials, as well as budgetary considerations. Prior to trial initiation, sites are evaluated by MSD personnel to assess the ability to successfully conduct the trial.

3. Site Monitoring/Scientific Integrity

Investigative trial sites are monitored to assess compliance with the trial protocol and general principles of Good Clinical Practice (GCP). MSD reviews clinical data for accuracy, completeness, and consistency. Data are verified versus source documentation according to standard operating procedures. Per MSD policies and procedures, if fraud, scientific/research misconduct, or serious GCP-noncompliance is suspected, the issues

are investigated. When necessary, the clinical site will be closed, the responsible regulatory authorities and ethics review committees notified.

B. Publication and Authorship

Regardless of trial outcome, MSD commits to publish primary and secondary results of its registered trials of marketed products in which treatment is assigned, according to the prespecified plans for data analysis. To the extent scientifically appropriate, MSD seeks to publish the results of other analyses it conducts that are important to patients, physicians, and payers. Some early phase or pilot trials are intended to be hypothesis-generating rather than hypothesis testing, in such cases, publication of results may not be appropriate since the trial may be underpowered and the analyses complicated by statistical issues such as multiplicity.

MSD's policy on authorship is consistent with the recommendations published by the International Committee of Medical Journal Editors (ICMJE). In summary, authorship should reflect significant contribution to the design and conduct of the trial, performance or interpretation of the analysis, and/or writing of the manuscript. All named authors must be able to defend the trial results and conclusions. MSD funding of a trial will be acknowledged in publications.

III. Participant Protection

A. Ethics Committee Review (Institutional Review Board [IRB]/Independent Ethics Committee [IEC])

All clinical trials will be reviewed and approved by an IRB/IEC before being initiated at each site. Significant changes or revisions to the protocol will be approved by the ethics committee prior to implementation, except changes required urgently to protect participant safety that may be enacted in anticipation of ethics committee approval. For each site, the ethics committee and MSD will approve the participant informed consent form.

B. Safety

The guiding principle in decision-making in clinical trials is that participant welfare is of primary importance. Potential participants will be informed of the risks and benefits of, as well as alternatives to, trial participation. At a minimum, trial designs will take into account the local standard of care.

All participation in MSD clinical trials is voluntary. Participants enter the trial only after informed consent is obtained. Participants may withdraw from an MSD trial at any time, without any influence on their access to, or receipt of, medical care that may otherwise be available to them.

C. Confidentiality

MSD is committed to safeguarding participant confidentiality, to the greatest extent possible. Unless required by law, only the investigator, Sponsor (or representative), ethics committee, and/or regulatory authorities will have access to confidential medical records that might identify the participant by name.

D. Genomic Research

Genomic research will only be conducted in accordance with a protocol and informed consent authorized by an ethics committee.

IV. Financial Considerations

A. Payments to Investigators

Clinical trials are time- and labor-intensive. It is MSD's policy to compensate investigators (or the sponsoring institution) in a fair manner for the work performed in support of MSD trials. MSD does not pay incentives to enroll participants in its trials. However, when enrollment is particularly challenging, additional payments may be made to compensate for the time spent in extra recruiting efforts.

MSD does not pay for participant referrals. However, MSD may compensate referring physicians for time spent on chart review to identify potentially eligible participants.

B. Clinical Research Funding

Informed consent forms will disclose that the trial is sponsored by MSD and that the investigator or sponsoring institution is being paid or provided a grant for performing the trial. However, the local ethics committee may wish to alter the wording of the disclosure statement to be consistent with financial practices at that institution. As noted above, all publications resulting from MSD trials will indicate MSD as a source of funding.

C. Funding for Travel and Other Requests

Funding of travel by investigators and support staff (eg, to scientific meetings, investigator meetings, etc.) will be consistent with local guidelines and practices.

V. Investigator Commitment

Investigators will be expected to review MSD's Code of Conduct as an appendix to the trial protocol, and in signing the protocol, agree to support these ethical and scientific standards.

10.1.2 Financial Disclosure

Financial Disclosure requirements are outlined in the US Food and Drug Administration Regulations, Financial Disclosure by Clinical Investigators (21 CFR Part 54). It is the Sponsor's responsibility to determine, based on these regulations, whether a request for Financial Disclosure information is required. It is the investigator's/subinvestigator's responsibility to comply with any such request.

The investigator/subinvestigator(s) agree, if requested by the Sponsor in accordance with 21 CFR Part 54, to provide his/her financial interests in and/or arrangements with the Sponsor to allow for the submission of complete and accurate certification and disclosure statements. The investigator/subinvestigator(s) further agree to provide this information on a Certification/Disclosure Form, commonly known as a financial disclosure form, provided by the Sponsor. The investigator/subinvestigator(s) also consent to the transmission of this information to the Sponsor in the United States for these purposes. This may involve the transmission of information to countries that do not have laws protecting personal data.

10.1.3 Data Protection

Participants will be assigned a unique identifier by the Sponsor. Any participant records or datasets that are transferred to the Sponsor will contain the identifier only; participant names or any information that would make the participant identifiable will not be transferred.

The participant must be informed that his/her personal study-related data will be used by the Sponsor in accordance with local data protection law. The level of disclosure must also be explained to the participant.

The participant must be informed that his/her medical records may be examined by Clinical Quality Assurance auditors or other authorized personnel appointed by the Sponsor, by appropriate IRB/IEC members, and by inspectors from regulatory authorities.

10.1.3.1 Confidentiality of Data

By signing this protocol, the investigator affirms to the Sponsor that information furnished to the investigator by the Sponsor will be maintained in confidence, and such information will be divulged to the IRB, IEC, or similar or expert committee; affiliated institution and employees, only under an appropriate understanding of confidentiality with such board or committee, affiliated institution and employees. Data generated by this study will be considered confidential by the investigator, except to the extent that it is included in a publication as provided in the Publications section of this protocol.

10.1.3.2 Confidentiality of Participant Records

By signing this protocol, the investigator agrees that the Sponsor (or Sponsor representative), IRB/IEC, or regulatory authority representatives may consult and/or copy study documents to verify worksheet/CRF report form data. By signing the consent form, the participant agrees to this process. If study documents will be photocopied during the process of verifying worksheet/CRF information, the participant will be identified by unique code only; full names/initials will be masked prior to transmission to the Sponsor.

By signing this protocol, the investigator agrees to treat all participant data used and disclosed in connection with this study in accordance with all applicable privacy laws, rules and regulations.

10.1.3.3 Confidentiality of IRB/IEC Information

The Sponsor is required to record the name and address of each IRB/IEC that reviews and approves this study. The Sponsor is also required to document that each IRB/IEC meets regulatory and ICH GCP requirements by requesting and maintaining records of the names and qualifications of the IRB/IEC members and to make these records available for regulatory agency review upon request by those agencies.

10.1.4 Committees Structure

Not applicable.

10.1.5 Publication Policy

The results of this study may be published or presented at scientific meetings. The Sponsor will comply with the requirements for publication of study results. In accordance with standard editorial and ethical practice, the Sponsor will generally support publication of multicenter studies only in their entirety and not as individual site data. In this case, a coordinating investigator will be designated by mutual agreement.

If publication activity is not directed by the Sponsor, the investigator agrees to submit all manuscripts or abstracts to the Sponsor before submission. This allows the Sponsor to protect proprietary information and to provide comments.

Authorship will be determined by mutual agreement and in line with International Committee of Medical Journal Editors authorship requirements.

10.1.6 Compliance with Study Registration and Results Posting Requirements

Under the terms of the Food and Drug Administration Amendments Act (FDAAA) of 2007 and the European Medicines Agency (EMA) clinical trial Directive 2001/20/EC, the Sponsor of the study is solely responsible for determining whether the study and its results are subject to the requirements for submission to http://www.clinicaltrials.gov, www.clinicaltrialsregister.eu or other local registries. MSD, as Sponsor of this study, will review this protocol and submit the information necessary to fulfill these requirements. MSD entries are not limited to FDAAA or the EMA clinical trial directive mandated trials. Information posted will allow participants to identify potentially appropriate studies for their disease conditions and pursue participation by calling a central contact number for further information on appropriate study locations and study site contact information.

By signing this protocol, the investigator acknowledges that the statutory obligations under FDAAA, the EMA clinical trials directive or other locally mandated registries are that of the Sponsor and agrees not to submit any information about this study or its results to those registries.

10.1.7 Compliance with Law, Audit, and Debarment

By signing this protocol, the investigator agrees to conduct the study in an efficient and diligent manner and in conformance with this protocol; generally accepted standards of GCP (eg, International Conference on Harmonization of Technical Requirements for Registration of Pharmaceuticals for Human Use GCP: Consolidated Guideline and other generally accepted standards of good clinical practice); and all applicable federal, state and local laws, rules and regulations relating to the conduct of the clinical study. The Code of Conduct, a collection of goals and considerations that govern the ethical and scientific conduct of clinical investigations sponsored by MSD, is provided in this appendix under the Code of Conduct for Clinical Studies.

The investigator agrees not to seek reimbursement from participants, their insurance providers, or from government programs for procedures included as part of the study reimbursed to the investigator by the Sponsor.

The investigator will promptly inform the Sponsor of any regulatory authority inspection conducted for this study.

The investigator agrees to provide the Sponsor with relevant information from inspection observations/findings to allow the Sponsor to assist in responding to any citations resulting from regulatory authority inspection and will provide the Sponsor with a copy of the proposed response for consultation before submission to the regulatory authority.

Persons debarred from conducting or working on clinical studies by any court or regulatory authority will not be allowed to conduct or work on this Sponsor's studies. The investigator will immediately disclose in writing to the Sponsor if any person who is involved in conducting the study is debarred or if any proceeding for debarment is pending or, to the best of the investigator's knowledge, threatened.

10.1.8 Data Quality Assurance

All participant data relating to the study will be recorded on printed or electronic CRF unless transmitted to the Sponsor or designee electronically (eg, laboratory data). The investigator or qualified designee is responsible for verifying that data entries are accurate and correct by physically or electronically signing the CRF.

Detailed information regarding Data Management procedures for this protocol will be provided separately.

The investigator must maintain accurate documentation (source data) that supports the information entered in the CRF.

The investigator must permit study-related monitoring, audits, IRB/IEC review, and regulatory agency inspections and provide direct access to source data documents.

Study documentation will be promptly and fully disclosed to the Sponsor by the investigator upon request and also shall be made available at the study site upon request for inspection, copying, review, and audit at reasonable times by representatives of the Sponsor or any regulatory authorities. The investigator agrees to promptly take any reasonable steps that are requested by the Sponsor or any regulatory authorities as a result of an audit or inspection to cure deficiencies in the study documentation and worksheets/CRFs.

The Sponsor or designee is responsible for the data management of this study including quality checking of the data.

Study monitors will perform ongoing source data review and verification to confirm that data entered into the CRF by authorized site personnel are accurate, complete, and verifiable from source documents; that the safety and rights of participants are being protected; and that the study is being conducted in accordance with the currently approved protocol and any other study agreements, ICH GCP, and all applicable regulatory requirements.

Records and documents, including signed ICF, pertaining to the conduct of this study must be retained by the investigator for 15 years after study completion unless local regulations or institutional policies require a longer retention period. No records may be destroyed during the retention period without the written approval of the Sponsor. No records may be transferred to another location or party without written notification to the Sponsor.

10.1.9 Source Documents

Source documents provide evidence for the existence of the participant and substantiate the integrity of the data collected. Source documents are filed at the investigator's site.

Data reported on the CRF or entered in the eCRF that are transcribed from source documents must be consistent with the source documents or the discrepancies must be explained. The investigator may need to request previous medical records or transfer records, depending on the study. Also, current medical records must be available.

10.1.10 Study and Site Closure

The Sponsor or its designee may stop the study or study site participation in the study for medical, safety, regulatory, administrative, or other reasons consistent with applicable laws, regulations, and GCP.

In the event the Sponsor prematurely terminates a particular study site, the Sponsor will promptly notify that study site's IRB/IEC.

10.2 Appendix 2: Clinical Laboratory Tests

- The tests detailed in Table 14 will be performed by the local laboratory.

- Protocol-specific requirements for inclusion or exclusion of participants are detailed in Section 5 of the protocol.

- Additional tests may be performed at any time during the study as determined necessary by the investigator or required by local regulations.

Table 14 Protocol-required Safety Laboratory Assessments

Laboratory Assessments	Parameters			
Hematology	Platelet Count<br>RBC Count<br>Hemoglobin<br>Hematocrit	RBC Indices:<br>MCV<br>MCH<br>%Reticulocytes	WBC count with Differential:<br>Neutrophils<br>Lymphocytes<br>Monocytes<br>Eosinophils<br>Basophils	
Chemistry	Blood Urea Nitrogen (BUN)	Potassium	Aspartate Aminotransferase (AST)/ Serum Glutamic-Oxaloacetic Transaminase (SGOT)	Total bilirubin (and direct bilirubin, if total bilirubin is elevated above the upper limit of normal)
	Albumin	Bicarbonate	Chloride	Phosphorous
	Creatinine	Sodium	Alanine Aminotransferase (ALT)/ Serum Glutamic-Pyruvic Transaminase (SGPT)	Total Protein
	Glucose	Calcium	Alkaline phosphatase	TSH<br>Total T3 (or free T3)<br>Total T4 (or free T4)[a]
Routine Urinalysis	Specific gravity<br>pH, glucose, protein, blood, ketones, [bilirubin, urobilinogen, nitrite, leukocyte esterase] by dipstick<br>Microscopic examination (if blood or protein is abnormal)			
Other Screening Tests	Follicle-stimulating hormone and estradiol (as needed in women of nonchildbearing potential only)<br>[Serum or urine] [alcohol and drug screen (to include at minimum: amphetamines, barbiturates, cocaine, opiates, cannabinoids and benzodiazepines) if applicable]<br>[Serum or urine] β-human chorionic gonadotropin (β-hCG) pregnancy test (as needed for WOCBP)<br>[Serology [(HIV antibody, hepatitis B surface antigen [HBsAg], and hepatitis C virus antibody)] [or specify other tests] [if applicable]			

NOTES:
[a]T3 and T4 are preferred; if not available, free T3 and free T4 may be tested.
Abbreviations: β-hCG=β-human chorionic gonadotropin; ALT=alanine transaminase; AST=aspartate transaminase; BUN=blood urea nitrogen; HBsAg=hepatitis B surface antigen; HIV=human immunodeficiency virus; MCH=mean corpuscular hemoglobin; MCV=mean corpuscular volume; RBC=red blood cell; SGOT=serum glutamic oxaloacetic transaminase; SGPT=serum glutamic pyruvic transaminase; TSH=thyroid stimulating hormone; WBC=white blood cell; WOCBP=woman/women of childbearing potential.

The investigator (or medically qualified designee) must document their review of each laboratory safety report.

10.3 Appendix 3: Adverse Events: Definitions and Procedures for Recording, Evaluating, Follow-up, and Reporting

10.3.1 Definition of AE

AE definition

- An AE is any untoward medical occurrence in a clinical study participant, temporally associated with the use of study intervention, whether or not considered related to the study intervention.

- NOTE: An AE can therefore be any unfavorable and unintended sign (including an abnormal laboratory finding), symptom, or disease (new or exacerbated) temporally associated with the use of a study intervention.

- NOTE: For purposes of AE definition, study intervention (also referred to as Sponsor's product) includes any pharmaceutical product, biological product, vaccine, device, diagnostic agent, or protocol specified procedure whether investigational (including placebo or active comparator product) or marketed, manufactured by, licensed by, provided by, or distributed by the Sponsor for human use in this study.

Events meeting the AE definition

- Any abnormal laboratory test results (hematology, clinical chemistry, or urinalysis) or other safety assessments (eg, ECG, radiological scans, vital signs measurements), including those that worsen from baseline, or are considered clinically significant in the medical and scientific judgment of the investigator.

- Exacerbation of a chronic or intermittent pre-existing condition including either an increase in frequency and/or intensity of the condition.

- New conditions detected or diagnosed after study intervention administration even though it may have been present before the start of the study.

- Signs, symptoms, or the clinical sequelae of a suspected drug-drug interaction.

- Signs, symptoms, or the clinical sequelae of a suspected overdose of either study intervention or a concomitant medication.

- For all reports of overdose (whether accidental or intentional) with an associated AE, the AE term should reflect the clinical symptoms or abnormal test result. An overdose without any associated clinical symptoms or abnormal laboratory results is reported using the terminology "accidental or intentional overdose without adverse effect."

Worsening of signs and symptoms of malignancy during the study should be reported as an AE in the appropriate section of the CRF. Disease progression assessed by measurement of malignant lesions on radiographs or other methods should not be reported as an AE, unless the event results in hospitalization or death.

Events NOT meeting the AE definition

- Medical or surgical procedure (eg, endoscopy, appendectomy): the condition that leads to the procedure is the AE.
- Situations in which an untoward medical occurrence did not occur (social and/or convenience admission to a hospital).
- Anticipated day-to-day fluctuations of pre-existing disease(s) or condition(s) present or detected at the start of the study that do not worsen.
- Surgery planned prior to informed consent to treat a pre-existing condition that has not worsened.
- Refer to Section 8.4.7 for protocol-specific exceptions.

10.3.2 Definition of SAE

If an event is not an AE per definition above, then it cannot be an SAE even if serious conditions are met.

An SAE is defined as any untoward medical occurrence that, at any dose:

a. **Results in death**

b. **Is life-threatening**

- The term “life-threatening” in the definition of “serious” refers to an event in which the participant was at risk of death at the time of the event. It does not refer to an event, which hypothetically might have caused death, if it were more severe.

c. **Requires inpatient hospitalization or prolongation of existing hospitalization**

- Hospitalization is defined as an inpatient admission, regardless of length of stay, even if the hospitalization is a precautionary measure for continued observation. (Note: Hospitalization for an elective procedure to treat a pre-existing condition that has not worsened is not an SAE. A pre-existing condition is a clinical condition that is diagnosed prior to the use of an MSD product and is documented in the participant’s medical history.

d. **Results in persistent or significant disability/incapacity**

- The term disability means a substantial disruption of a person's ability to conduct normal life functions.

- This definition is not intended to include experiences of relatively minor medical significance such as uncomplicated headache, nausea, vomiting, diarrhea, influenza, and accidental trauma (eg, sprained ankle) that may interfere with or prevent everyday life functions but do not constitute a substantial disruption.

e. **Is a congenital anomaly/birth defect**

- In offspring of participant taking the product regardless of time to diagnosis.

f. **Other important medical events**

- Medical or scientific judgment should be exercised in deciding whether SAE reporting is appropriate in other situations such as important medical events that may not be immediately life-threatening or result in death or hospitalization but may jeopardize the participant or may require medical or surgical intervention to prevent 1 of the other outcomes listed in the above definition. These events should usually be considered serious.

  Examples of such events include invasive or malignant cancers, intensive treatment in an emergency room or at home for allergic bronchospasm, blood dyscrasias or convulsions that do not result in hospitalization, or development of drug dependency or drug abuse.

10.3.3 Additional Events Reported in the Same Manner as SAE

Additional events that require reporting in the same manner as SAE

In addition to the above criteria, AEs meeting either of the below criteria, although not serious per ICH definition, are reportable to the Sponsor in the same timeframe as SAEs to meet certain local requirements. Therefore, these events are considered serious by the Sponsor for collection purposes.

- Is a new cancer (that is not a condition of the study)

- Is associated with an overdose

10.3.4 Recording AE and SAE

AE and SAE recording

- When an AE/SAE occurs, it is the responsibility of the investigator to review all documentation (eg, hospital progress notes, laboratory, and diagnostics reports) related to the event.

- The investigator will record all relevant AE/SAE information on the AE CRFs/worksheets at each examination.

- It is not acceptable for the investigator to send photocopies of the participant's medical records to the Sponsor in lieu of completion of the AE CRF page.

- There may be instances when copies of medical records for certain cases are requested by the Sponsor. In this case, all participant identifiers, with the exception of the participant number, will be blinded on the copies of the medical records before submission to the Sponsor.

- The investigator will attempt to establish a diagnosis of the event based on signs, symptoms, and/or other clinical information. In such cases, the diagnosis (not the individual signs/symptoms) will be documented as the AE/SAE.

Assessment of intensity

- An event is defined as "serious" when it meets at least 1 of the predefined outcomes as described in the definition of an SAE, not when it is rated as severe.

Assessment of causality

Did the Sponsor's product cause the AE?

The determination of the likelihood that the Sponsor's product caused the AE will be provided by an investigator who is a qualified physician. The investigator's signed/dated initials on the source document or worksheet that supports the causality noted on the AE form, ensures that a medically qualified assessment of causality was done. This initialed document must be retained for the required regulatory time frame. The criteria below are intended as reference guidelines to assist the investigator in assessing the likelihood of a relationship between the test product and the AE based upon the available information.

The following components are to be used to assess the relationship between the Sponsor's product and the AE; the greater the correlation with the components and their respective elements (in number and/or intensity), the more likely the Sponsor's product caused the AE:

Exposure: Is there evidence that the participant was actually exposed to the Sponsor's product such as: reliable history, acceptable compliance assessment (pill count, diary, etc.), expected pharmacologic effect, or measurement of drug/metabolite in bodily specimen?

Time Course: Did the AE follow in a reasonable temporal sequence from administration of the Sponsor's product? Is the time of onset of the AE compatible with a drug-induced effect (applies to studies with investigational medicinal product)?

Likely Cause: Is the AE not reasonably explained by another etiology such as underlying disease, other drug(s)/vaccine(s), or other host or environmental factors.

Dechallenge: Was the Sponsor's product discontinued or dose/exposure/frequency reduced?

- If yes, did the AE resolve or improve?
- If yes, this is a positive dechallenge.
- If no, this is a negative dechallenge.

(Note: This criterion is not applicable if: (1) the AE resulted in death or permanent disability; (2) the AE resolved/improved despite continuation of the Sponsor's product; (3) the study is a single-dose drug study; or (4) Sponsor's product(s) is/are only used 1 time.)

Rechallenge: Was the participant re-exposed to the Sponsor's product in this study?

- If yes, did the AE recur or worsen?
- If yes, this is a positive rechallenge.
- If no, this is a negative rechallenge.

(Note: This criterion is not applicable if: (1) the initial AE resulted in death or permanent disability, or (2) the study is a single-dose drug study; or (3) Sponsor's product(s) is/are used only 1 time.)

NOTE: IF A RECHALLENGE IS PLANNED FOR AN AE THAT WAS SERIOUS AND MAY HAVE BEEN CAUSED BY THE SPONSOR'S PRODUCT, OR IF RE-EXPOSURE TO THE SPONSOR'S PRODUCT POSES ADDITIONAL POTENTIAL SIGNIFICANT RISK TO THE PARTICIPANT THEN THE RECHALLENGE MUST BE APPROVED IN ADVANCE BY THE SPONSOR CLINICAL DIRECTOR AS PER DOSE MODIFICATION GUIDELINES IN THE PROTOCOL, AND IF REQUIRED, THE INIRB/IEC.

Consistency with study intervention profile: Is the clinical/pathological presentation of the AE consistent with previous knowledge regarding the Sponsor's product or drug class pharmacology or toxicology?

The assessment of relationship will be reported on the case report forms/worksheets by an investigator who is a qualified physician according to his/her best clinical judgment, including consideration of the above elements.

Use the following scale of criteria as guidance (not all criteria must be present to be indicative of a Sponsor's product relationship).

- Yes, there is a reasonable possibility of Sponsor's product relationship:

There is evidence of exposure to the Sponsor's product. The temporal sequence of the AE onset relative to the administration of the Sponsor's product is reasonable. The AE is more likely explained by the Sponsor's product than by another cause.

- No, there is not a reasonable possibility of Sponsor's product relationship:

Participant did not receive the Sponsor's product OR temporal sequence of the AE onset relative to administration of the Sponsor's product is not reasonable OR the AE is more likely explained by another

Follow-up of AE and SAE

- The investigator is obligated to perform or arrange for the conduct of supplemental measurements and/or evaluations as medically indicated or as requested by Sponsor to elucidate the nature and/or causality of the AE or SAE as fully as possible. This may include additional laboratory tests or investigations, histopathological examinations, or consultation with other health care professionals.

- New or updated information will be recorded in the CRF.

- The investigator will submit any updated SAE data to the Sponsor within 24 hours of receipt of the information.

10.3.5 Reporting of AE, SAE, and Other Reportable Safety Events to the Sponsor

AE, SAE, and other reportable safety event reporting to Sponsor via electronic data collection tool

- The primary mechanism for reporting to the Sponsor will be the electronic data collection (EDC) tool.
  - Electronic reporting procedures can be found in the EDC data entry guidelines (or equivalent).
  - If the electronic system is unavailable for more than 24 hours, then the site will use the paper AE Reporting form.

- Reference Section 8.4.1 for reporting time requirements.

- The site will enter the SAE data into the electronic system as soon as it becomes available.

- After the study is completed at a given site, the EDC tool will be taken off-line to prevent the entry of new data or changes to existing data.

- If a site receives a report of a new SAE from a study participant or receives updated data on a previously reported SAE after the EDC tool has been taken off-line, then the site can report this information on a paper SAE form or by telephone (see next section).

- Contacts for SAE reporting can be found in the Investigator Study File Binder (or equivalent).

SAE reporting to the Sponsor via paper CRF

- If the EDC tool is not operational, facsimile transmission or secure e-mail of the SAE paper CRF is the preferred method to transmit this information to the Sponsor.

- In rare circumstances and in the absence of facsimile equipment, notification by telephone is acceptable with a copy of the SAE data collection tool sent by overnight mail or courier service.

- Initial notification via telephone does not replace the need for the investigator to complete and sign the SAE CRF pages within the designated reporting time frames.

- Contacts and instructions for SAE reporting and paper reporting procedures can be found in the Investigator Study File Binder (or equivalent).

10.4 Appendix 4: Medical Device Incidents: Definition and Procedures for Recording, Evaluating, Follow-up, and Reporting

Not Applicable.

10.5 Appendix 5: Contraceptive Guidance and Pregnancy Testing

10.5.1 Definitions

Women of Childbearing Potential (WOCBP)

A woman is considered fertile following menarche and until becoming postmenopausal unless permanently sterile.

Women in the following categories are not considered WOCBP:

- Premenarchal
- Premenopausal female with 1 of the following:
  - Documented hysterectomy
  - Documented bilateral salpingectomy
  - Documented bilateral oophorectomy

Note: Documentation can come from the site personnel's review of the participant's medical records, medical examination, or medical history interview.

- Postmenopausal female
  - A postmenopausal state is defined as no menses for 12 months without an alternative medical cause.
- A high follicle-stimulating hormone (FSH) level in the postmenopausal range may be used to confirm a postmenopausal state in women not using hormonal contraception or hormone replacement therapy (HRT). However, in the absence of 12 months of amenorrhea, confirmation with 2 FSH measurements in the postmenopausal range is required.
  - Females on HRT and whose menopausal status is in doubt will be required to use 1 of the nonhormonal highly effective contraception methods if they wish to continue their HRT during the study. Otherwise, they must discontinue HRT to allow confirmation of postmenopausal status before study enrollment.

10.5.2 Contraception Requirements

Female Participants

Female participants of childbearing potential are eligible to participate if they agree to use a highly effective method of contraception consistently and correctly as described in Table 15 during the protocol-defined time frame in Section 6.1.

Table 15 Highly Effective Contraception Methods

**Highly Effective Contraceptive Methods That Are User Dependent[a]**<br>*Failure rate of <1% per year when used consistently and correctly.*
● Combined (estrogen- and progestogen- containing) hormonal contraception[b]<br>○ Oral<br>○ Intravaginal<br>○ Transdermal<br>○ Injectable
● Progestogen-only hormonal contraception[b]<br>○ Oral<br>○ Injectable
**Highly Effective Methods That Have Low User Dependency**<br>*Failure rate of <1% per year when used consistently and correctly.*
● Progestogen- only contraceptive implant[b]<br>● Intrauterine hormone-releasing system (IUS)<br>● Intrauterine device (IUD)<br>● Bilateral tubal occlusion
● **Vasectomized partner**<br>A vasectomized partner is a highly effective contraception method provided that the partner is the sole male sexual partner of the WOCBP and the absence of sperm has been confirmed. If not, an additional highly effective method of contraception should be used.
● **Sexual abstinence**<br>Sexual abstinence is considered a highly effective method only if defined as refraining from heterosexual intercourse during the entire period of risk associated with the study treatment. The reliability of sexual abstinence needs to be evaluated in relation to the duration of the study and the preferred and usual lifestyle of the participant.
Notes:<br>Use should be consistent with local regulations regarding the use of contraceptive methods for participants of clinical studies.<br>a) Typical use failure rates are lower than perfect-use failure rates (i.e. when used consistently and correctly).<br>b) If locally required, in accordance with Clinical Trial Facilitation Group (CTFG) guidelines, acceptable hormonal contraceptives are limited to those which inhibit ovulation.

10.5.3 Pregnancy Testing

Women of childbearing potential should only be included after a negative highly sensitive urine or serum pregnancy test.

Following initiation of treatment, pregnancy testing will be performed whenever an expected menstrual cycle is missed or when pregnancy is otherwise suspected; at the time points specified in the Schedule of Activities, and as required locally.

Pregnancy testing will be performed whenever an expected menstrual cycle is missed or when pregnancy is otherwise suspected.

10.6 Appendix 6: Collection and Management of Specimens for Future Biomedical Research

Not applicable.

10.7 Appendix 7: Country-specific Requirements

Not applicable.

10.8 Appendix 8: Description of the iRECIST Process for Assessment of Disease Progression

Assessment at Screening and Prior to RECIST 1.1 Progression

Until radiographic disease progression based on RECIST 1.1, there is no distinct iRECIST assessment.

Assessment and Decision at RECIST 1.1 Progression

For participants who show evidence of radiological PD by RECIST 1.1 as determined by the investigator, the investigator will decide whether to continue a participant on study intervention until repeat imaging 4 to 8 weeks later is obtained (using iRECIST for participant management (see Table 7 and Figure 2). This decision by the investigator should be based on the participant's overall clinical condition.

Clinical stability is defined as the following:

- Absence of symptoms and signs indicating clinically significant progression of disease
- No decline in ECOG performance status
- No requirements for intensified management, including increased analgesia, radiation, or other palliative care

Any participant deemed clinically unstable should be discontinued from study intervention at site-assessed first radiologic evidence of PD, and is not required to have repeat tumor imaging for confirmation of PD by iRECIST.

If the investigator decides to continue treatment, the participant may continue to receive study intervention and the tumor assessment should be repeated 4 to 8 weeks later to confirm PD by iRECIST, per investigator assessment. Images should continue to be sent in to the iCRO for potential retrospective BICR (Cohort B).

Tumor flare may manifest as any factor causing radiographic progression per RECIST 1.1, including:

- Increase in the sum of diameters of target lesion(s) identified at baseline to ≥20% and ≥5 mm from nadir
  Note: The iRECIST publication uses the terminology "sum of measurements," but "sum of diameters" will be used in this protocol, consistent with the original RECIST 1.1 terminology.
- Unequivocal progression of nontarget lesion(s) identified at baseline

- Development of new lesion(s)

iRECIST defines new response categories, including iUPD (unconfirmed progressive disease) and iCPD (confirmed progressive disease). For purposes of iRECIST assessment, the first visit showing progression according to RECIST 1.1 will be assigned a visit (overall) response of iUPD, regardless of which factors caused the progression.

At this visit, target and nontarget lesions identified at baseline by RECIST 1.1 will be assessed as usual.

New lesions will be classified as measurable or nonmeasurable, using the same size thresholds and rules as for baseline lesion assessment in RECIST 1.1. From measurable new lesions, up to 5 lesions total (up to 2 per organ), may be selected as New Lesions – Target. The sum of diameters of these lesions will be calculated, and kept distinct from the sum of diameters for target lesions at baseline. All other new lesions will be followed qualitatively as New Lesions – Non-target.

Assessment at the Confirmatory Imaging

On the confirmatory imaging, the participant will be classified as progression confirmed (with an overall response of iCPD), or as showing persistent unconfirmed progression (with an overall response of iUPD), or as showing disease stability or response (iSD/iPR/iCR).

Confirmation of Progression

Progression is considered confirmed, and the overall response will be iCPD, if ANY of the following occurs:

- Any of the factors that were the basis for the iUPD at the previous visit show worsening
    - For target lesions, worsening is a further increase in the sum of diameters of ≥5 mm, compared to any prior iUPD time point
    - For nontarget lesions, worsening is any significant growth in lesions overall, compared to a prior iUPD time point; this does not have to meet the “unequivocal” standard of RECIST 1.1
    - For new lesions, worsening is any of these:
        - An increase in the new lesion sum of diameters by ≥5 mm from a prior iUPD time point
        - Visible growth of new nontarget lesions
        - The appearance of additional new lesions

- Any new factor appears that would have triggered PD by RECIST 1.1

Persistent iUPD

Progression is considered not confirmed, and the overall response remains iUPD, if:

- None of the progression-confirming factors identified above occurs AND
- The target lesion sum of diameters (initial target lesions) remains above the initial PD threshold (by RECIST 1.1)

Additional imaging for confirmation should be scheduled 4 to 8 weeks from the imaging on which iUPD is seen. This may correspond to the next visit in the original visit schedule. The assessment of the subsequent confirmation imaging proceeds in an identical manner, with possible outcomes of iCPD, iUPD, and iSD/iPR/iCR.

Resolution of iUPD

Progression is considered not confirmed, and the overall response becomes iSD/iPR/iCR, if:

- None of the progression-confirming factors identified above occurs, AND
- The target lesion sum of diameters (initial target lesions) is not above the initial PD threshold.

The response is classified as iSD or iPR (depending on the sum of diameters of the target lesions), or iCR if all lesions resolve.

In this case, the initial iUPD is considered to be pseudo-progression, and the level of suspicion for progression is "reset." This means that the next visit that shows radiographic progression, whenever it occurs, is again classified as iUPD by iRECIST, and the confirmation process is repeated before a response of iCPD can be assigned.

Management Following the Confirmatory Imaging

If repeat imaging does not confirm PD per iRECIST, as assessed by the investigator, and the participant continues to be clinically stable, study treatment may continue and follow the regular imaging schedule. If PD is confirmed, participants will be discontinued from study treatment.

NOTE: If a participant has confirmed radiographic progression (iCPD) as defined above, but the participant is achieving a clinically meaningful benefit, an exception to continue study intervention may be considered following consultation with the Sponsor. In this case, if study intervention is continued, tumor imaging should continue to be performed following the intervals as outlined in Section 1.3 and submitted to the iCRO (Cohort B).

Detection of Progression at Visits After Pseudo-progression Resolves

After resolution of pseudo-progression (ie, achievement of iSD/iPR/iCR), iUPD is indicated by any of the following events:

- Target lesions
  - Sum of diameters reaches the PD threshold (≥20% and ≥5 mm increase from nadir) either for the first time, or after resolution of previous pseudo-progression. The nadir is always the smallest sum of diameters seen during the entire study, either before or after an instance of pseudo-progression.
- Nontarget lesions
  - If nontarget lesions have never shown unequivocal progression, their doing so for the first time results in iUPD.
  - If nontarget lesions have shown previous unequivocal progression, and this progression has not resolved, iUPD results from any significant further growth of non-target lesions, taken as a whole.
- New lesions
  - New lesions appear for the first time
  - Additional new lesions appear
  - Previously identified new target lesions show an increase of ≥5 mm in the new lesion sum of diameters, from the nadir value of that sum
  - Previously identified non-target lesions show any significant growth

If any of the events above occur, the overall response for that visit is iUPD, and the iUPD evaluation process (see Assessment at the Confirmatory Imaging above) is repeated. Progression must be confirmed before iCPD can occur.

The decision process is identical to the iUPD confirmation process for the initial PD, with one exception: If new lesions occurred at a prior instance of iUPD, and at the confirmatory imaging the burden of new lesions has increased from its smallest value (for new target lesions, the sum of diameters is ≥5 mm increased from its nadir), then iUPD cannot resolve to iSD or iPR. It will remain iUPD until either a decrease in the new lesion burden allows resolution to iSD or iPR, or until a confirmatory factor causes iCPD.

Additional details about iRECIST are provided in the iRECIST publication [Seymour, L., et al 2017].

10.9 Appendix 9: Abbreviations

Abbreviation	Expanded Term
β-HCG	beta-human chorionic gonadotropin
ADA	antidrug antibodies
AE	adverse event
AJCC	American Joint Committee on Cancer
ALT	alanine transaminase
APaT	All Patients as Treated
AST	aspartate transaminase
AUC	area under the curve
BCG	Bacillus Calmette-Guérin
BICR	Blinded Independent Central Review
C	Cycle
C_{avg}	average concentration
CBC	complete blood count
CD	cluster of differentiation
CI	confidence interval
CL	clearance
C_{max}	maximum concentration
C_{min}	minimum concentration
CNS	central nervous system
CONSORT	Consolidated Standards of Reporting Trials
CR	complete response
CRF	case report form
CRO	Contract Research Organization
CSR	Clinical Study Report
CT	computed tomography
CTCAE	Common Terminology Criteria for Adverse Events
CTLA-4	cytotoxic T-lymphocyte-associated protein 4
CV	coefficient of variation
D	Day
DILI	drug-induced liver injury
DOR	duration of response
ECG	electrocardiogram
ECI	event of clinical interest
ECOG	Eastern Cooperative Oncology Group
eCRF	electronic case report form
EDC	electronic data collection
EMA	European Medicines Agency
E-R	exposure-response
EU	European Union
F	Bioavailability
FAS	Full Analysis Set
FDAAA	Food and Drug Administration Amendments Act
FSH	Follicle-stimulating hormone
GCP	Good Clinical Practice
GI	gastrointestinal
GMR	geometric mean ratio
HBsAg	hepatitis B surface antigen
HCV	hepatitis c virus
HIV	human immunodeficiency virus

Abbreviation	Expanded Term
HRT	hormone replacement therapy
IB	Investigator's Brochure
ICF	Informed Consent Form
ICH	International Conference on Harmonization
ICMJE	International Committee of Medical Journal Editors
iCPD	confirmed radiographic progression
iCR	iRECIST complete response
iCRO	Imaging Contract Research Organization
IEC	Independent Ethics Committee
Ig	immunoglobulin
IgG	immunoglobulin G
IMP	investigational medicinal product
INR	international normalized ratio
iPR	iRECIST confirmed partial response
irAE	immune related adverse event
IRB	Institutional Review Board
iRECIST	modified Response Evaluation Criteria in Solid Tumors
iSD	iRECIST stable disease
iUPD	iRECIST unconfirmed progressive disease
IV	intravenous
Ka	absorption rate
LDH	lactase dehydrogenase
M&S	Modelling and Simulation
mAB	monoclonal antibody
MRI	magnetic resonance imaging
NCI	National Cancer Institute
NIMP	non-investigational medicinal product
NSAE	non-serious adverse event
NSAID	non-steroidal anti-inflammatory
NSCLC	non-small cell lung carcinoma
ORR	objective response rate
OS	overall survival
OTC	over-the-counter
PBPK	physiologically-based pharmacokinetics
PD	Progressive disease
PD-1	programmed cell death 1
PD-L1	programmed cell death ligand 1
PD-L2	programmed cell death ligand 2
PFS	progression free survival
PK	pharmacokinetic
PO	oral
PP	Per Protocol
PR	partial response
pRBC	packed red blood cell
PT	partial thromboplastin
PTT	partial thromboplastin time
Q2W	every 2 weeks
Q3W	every 3 weeks
Q6W	every 6 weeks
RBC	red blood cell
RNA	ribonucleic acid

Abbreviation	Expanded Term
SAE	serious adverse event
SC	subcutaneous
SGOT	serum glutamic oxaloacetic transaminase
SGPT	serum glutamic pyruvic transaminase
SLAB	Supplemental laboratory procedures
SoA	schedule of activities
SOP	Standard Operating Procedure
SUSAR	suspected unexpected serious adverse reaction
TMDD	target-mediated drug disposition
TSH	thyroid-stimulating hormone
T_{max}	time of maximum concentration
ULN	upper limit of normal
US	United States
V	volume of distribution
Vc	central volume of distribution
WBC	white blood cell
WOCBP	woman/women of childbearing potential

11 REFERENCES

[Chemnitz, J. M., et al 2004]	Chemnitz JM, Parry RV, Nichols KE, June CH, Riley JL. SHP-1 and SHP-2 associate with immunoreceptor tyrosine-based switch motif of programmed death 1 upon primary human T cell stimulation, but only receptor ligation prevents T cell activation. J Immunol 2004;173:945-54.	[00VMPN]
[Dirks, N. L. 2010]	Dirks NL, Meibohm B. Population pharmacokinetics of therapeutic monoclonal antibodies. Clin Pharmacokinet. 2010;49(10):633-59.	[04XMZG]
[Disis, M. L. 2010]	Disis ML. Immune regulation of cancer. J Clin Oncol 2010;28(29):4531-8.	[058SQL]
[Dudley, M. E., et al 2005]	Dudley ME, Wunderlich JR, Yang JC, Sherry RM, Topalian SL, Restifo NP, et al. Adoptive cell transfer therapy following non-myeloablative but lymphodepleting chemotherapy for the treatment of patients with refractory metastatic melanoma. J Clin Oncol 2005;23(10):2346-57.	[00VMPR]
[Francisco, L. M., et al 2010]	Francisco LM, Sage PT, Sharpe AH. The PD-1 pathway in tolerance and autoimmunity. Immunol Rev 2010;236:219-42.	[058SQP]
[Greenwald, R. J., et al 2005]	Greenwald RJ, Freeman GJ, Sharpe AH. The B7 family revisited. Annu Rev Immunol 2005;23:515-48.	[00VMQL]

[Hodi, F. S., et al 2014]	Hodi FS, Ribas A, Daud A, Hamid O, Robert C, Kefford R, et al. Patterns of response in patients with advanced melanoma treated with Pembrolizumab (MK-3475) and evaluation of immune-related response criteria (irRC). J Immunother Cancer. 2014;2(Suppl 3):P103.	[0465RW]
[Hunder, N. N., et al 2008]	Hunder NN, Wallen H, Cao J, Hendricks DW, Reilly JZ, Rodmyre R, et al. Treatment of metastatic melanoma with autologous CD4+ T cells against NY-ESO-1. N Engl J Med 2008;358(25):2698-703.	[00VMPX]
[Okazaki, T., et al 2001]	Okazaki T, Maeda A, Nishimura H, Kurosaki T, Honjo T. PD-1 immunoreceptor inhibits B cell receptor-mediated signaling by recruiting src homology 2-domain-containing tyrosine phosphatase 2 to phosphotyrosine. Proc Natl Acad Sci U S A 2001;98(24):13866-71.	[00VMQ6]
[Parry, R. V., et al 2005]	Parry RV, Chemnitz JM, Frauwirth KA, Lanfranco AR, Braunstein I, Kobayashi SV, et al. CTLA-4 and PD-1 receptors inhibit T-cell activation by distinct mechanisms. Mol Cell Biol 2005;25(21):9543-53.	[00VMQ7]
[Riley, J. L. 2009]	Riley JL. PD-1 signaling in primary T cells. Immunol Rev 2009;229:114-25.	[00VMQ9]
[Seymour, L., et al 2017]	Seymour L, Bogaerts J, Perrone A, Ford R, Schwartz LH, Mandrekar S, et al. iRECIST: guidelines for response criteria for use in trials testing immunotherapeutics. Lancet Oncol. 2017 Mar;18(3):e143-52.	[04P9RV]

[Sheppard, K-A, et al 2004]	Sheppard K-A, Fitz LJ, Lee JM, Benander C, George JA, Wooters J, et al. PD-1 inhibits T-cell receptor induced phosphorylation of the ZAP70/CD3zeta signalosome and downstream signaling to PKCtheta. FEBS Lett. 2004;574:37-41.	[00VMQC]
[Wolchok, J. D., et al 2009]	Wolchok JD, Hoos A, O'Day S, Weber JS, Hamid O, LebbéC, et al. Guidelines for the evaluation of immune therapy activity in solid tumors: immune-related response criteria. Clin Cancer Res 2009;15(23):7412-20.	[00VMNZ]
[Zhang, X., et al 2004]	Zhang X, Schwartz J-CD, Guo X, Bhatia S, Cao E, Chen L, et al. Structural and functional analysis of the costimulatory receptor programmed death-1. Immunity 2004;20:337-47.	[00VMQJ]
[Zhao, L., et al 2013]	Zhao L, Ji P, Li Z, Roy P, Sahajwalla CG. The antibody drug absorption following subcutaneous or intramuscular administration and its mathematical description by coupling physiologically based absorption process with the conventional compartment pharmacokinetic model. J Clin Pharmacol. 2013;53(3):314-25.	[04XV38]
